# Recent advancements in the chemistry of Diels–Alder reaction for total synthesis of natural products: a comprehensive review (2020–2023)

**DOI:** 10.1039/d4ra07989b

**Published:** 2025-02-10

**Authors:** Anitesh Rana, Anupam Mishra, Satish K. Awasthi

**Affiliations:** a Chemical Biology Laboratory, Department of Chemistry, University of Delhi Delhi 110007 India Satishpna@gmail.com

## Abstract

Despite being discovered nearly a century ago, the Diels–Alder (DA) reaction remains a crucial tool in the total synthesis of natural products. It accommodates a broad range of building blocks with varying complexity and levels of derivatization, allowing the formation of six-membered rings with precise stereochemistry. This, in turn, simplifies the synthesis of core structures found in many natural products. In recent years, modifications to the traditional Diels–Alder reaction have expanded its scope. These modifications include the inverse electron demand Diels–Alder reaction, dehydro Diels–Alder reaction, hetero-Diels–Alder reaction, photoenolization Diels–Alder reaction, asymmetric Diels–Alder reaction, and domino Diels–Alder reaction have been employed to extend the scope of this process in the synthesis of natural products. This review discusses the application of the Diels–Alder reaction in the total synthesis of natural products from 2020 to 2023, along with select methodologies that are inspired by or can be used to synthesize natural products.

## Abstract 2

This review covers recent advancements in Diels–Alder reaction chemistry for total synthesis of Natural Products (2020–2023) and, where relevant, discusses key structural features and biological activity of bioactive natural products.

## Graphical abstract

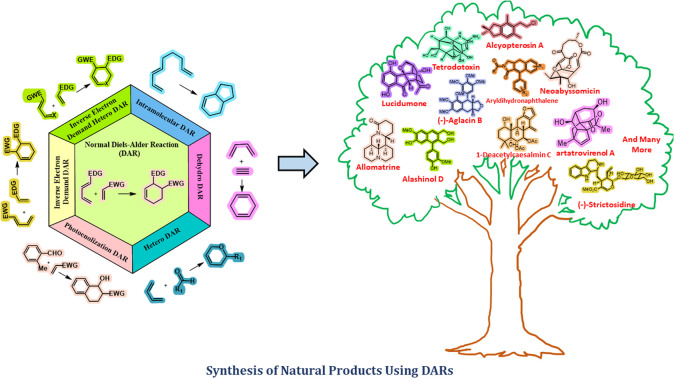

## 1. Introduction

Natural products are renowned for their remarkable structural diversity and complexity, and have played a pivotal role in advancing chemical biology and driving the discovery of new pharmacophores for drug development.^1–4^ The abundance of heteroatom substituents and stereocenters in natural products enhances their selective binding affinity for specific biological targets, often surpassing that of synthetic compounds.^5^ Additionally, the diverse functional groups in these natural products enhance their cell permeability, positioning them as promising candidates for drug discovery.^6^ Nevertheless, natural sources are unable to sustainably supply these compounds in their native or modified forms to meet rising demand. As a result, total synthesis has become a viable alternative for producing bioactive natural products and their analogues, utilizing scalable techniques such as diverted organic synthesis (DOS) and function-directed synthesis (FDS). Diels–Alder reactions are among the most efficient methods for the total synthesis of natural products, particularly due to the polycyclic structures present in many natural compounds.

**Scheme 1:**
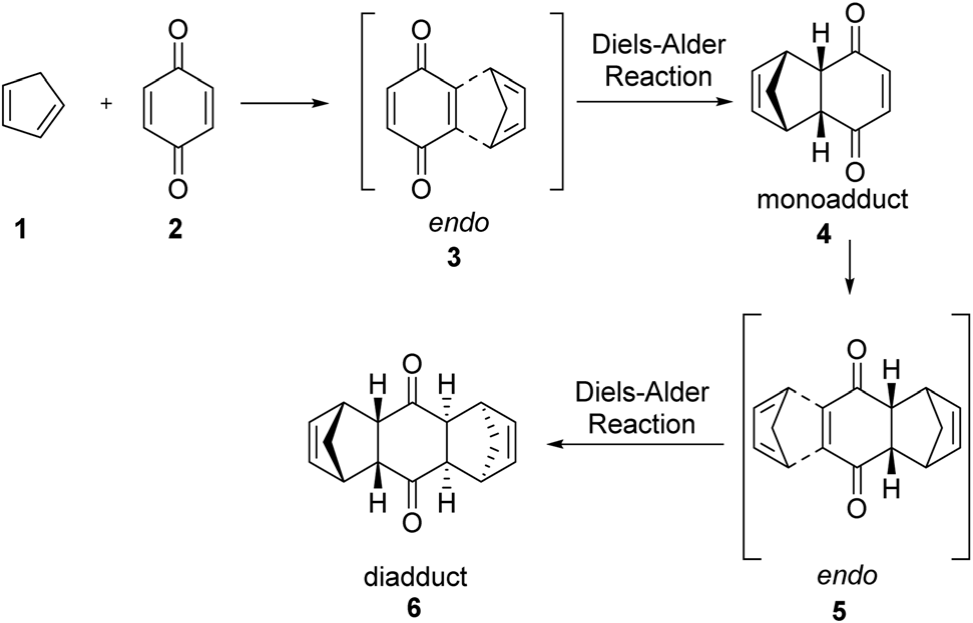
The reaction discovered by Diels and Alder in 1928.^7^

**Fig. 1:**
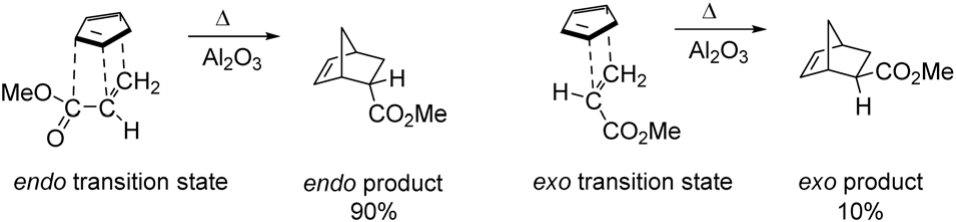
The orbital interactions between the diene and dienophile.^15^

In 1928, Otto Diels and Kurt Alder^7^ developed an extraordinary method for diene synthesis, named Diels–Alder reaction (Scheme 1). A conjugated diene 1 and a dienophile 2 are involved in this reaction, which is a through [4+2] cycloaddition reaction that forms a six-membered ring having up to four stereogenic centres. Since it follows a concerted reaction path, the Diels–Alder reaction affords high stereospecificity. The stereochemistry of the product, formed by cycloaddition reaction between electron-rich 4π and electron-poor 2π system, was explained by FMO theory.^8,9^ In case of thermally allowed Diels–Alder reaction between 1,3-butadiene derivatives and ethylene there is a primary interaction and a secondary interaction.^10–12^ The secondary interaction produced a kinetic *endo* product in major amount than the thermodynamically stable *exo* product.^13^ In 1990, Hondrogiannis *et al.*^14^ explained the *endo* selectivity by illustrating an example where *endo* product formed in 9 : 1 ratio (Fig. 1).^14^

Generally, the Diels–Alder reactions (DARs) were done under thermal conditions, but were accelerated by using Lewis acid catalysis, which was done by Yates and Eaton in 1960.^15^

**Fig. 2:**
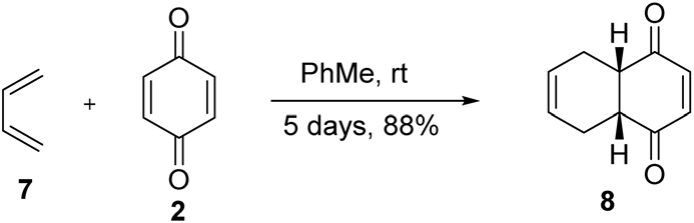
Tririya and Zanger's work in 2004.^16^

Moreover, in 2004, Tririya and Zanger^16^ reported that the Diels–Alder reaction can proceed at room temperature in quantitative yield by making the diene 7 more electron rich or the dienophile 2 more electron deficient (Fig. 2).^16^ Different kinds of substrates can participate in a Diels–Alder reaction. Due to the high versatility and huge applications of Diels–Alder reactions, the Nobel Prize in Chemistry was awarded to Otto Diels and Kurt Alder in 1950.

The use of Diels–Alder reactions in total synthesis was not taken up until Woodward *et al.* reported the total synthesis of cortisone, cholesterol in 1952 followed by reserpine in 1956, all of which involved the Diels–Alder reaction.^17,18^ These two total syntheses of natural products have opened a new avenue in total synthesis using Diels–Alder reactions (DARs) and proven effective in constructing various natural products and complex architectures.

Previous studies have emphasized the diverse applications of Diels–Alder reactions. Bhat *et al.*^19^ highlighted advancements in the inverse electron demand Diels–Alder reaction, particularly its role in the total synthesis of bioactive natural products over a thirteen-year period (2011 to 2023). Similarly, Kalesse *et al.*^20^ (2022) presented a review focusing on the applications of the Diels–Alder reaction in natural product synthesis from 2017 to 2020. Their review also explored methodologies inspired by or applicable to natural product synthesis, encompassing intermolecular, intramolecular, hetero, dehydro, inverse electron demand hetero, and aza-Diels–Alder reactions. These approaches have been instrumental in the total synthesis of various polycyclic natural products with significant biological activities, including anti-tumor, antioxidant, anti-malarial, anti-cancer, anti-microbial, and anti-inflammatory properties.^19–23^

In this review, we aim to spotlight key Diels–Alder reactions reported in the literature between 2020 and 2023 that offer new strategies and approaches for the total synthesis of natural products using normal Diels–Alder (DA) reaction, Intramolecular Diels–Alder (IMDA) reaction, Dehydro Diels–Alder (DDA) reaction, Hetero-Diels–Alder (HDA) reaction, Photoenolization Diels–Alder (PEDA) reaction, Inverse Electron Demand and Hetero-Diels–Alder (IEDDA) reaction.

In this study, we present the total synthesis of approximately 80 natural products utilizing Diels–Alder reactions, featuring key scaffolds such as *para*-hydroquinone (*p*-HQ), azulene phthalimides, tetrahydrocarbazoles, tetra and tri cyclic cores, spirocyclanes, terpinen-4-ol, fused pyridines, 6/6/5-fused tricyclic terpenoids, aryldihydronaphthalenes, spirocyclic cores, *cis*-decalins, spiroimines, arylnaphthalene lignans, tetrahydroquinolines, 6-azaindoles, tricyclic tetrahydropyrans, chromenones, tetracyclic isochromans, tetrahydrochromeno[4,3-*b*]quinolines, and *cis*-hydroindoles, which are commonly found in pharmacologically active compounds.^24^ Furthermore, where applicable, we discuss key structural features and biological activity characteristics of the bioactive natural products. This review thus summarizes recent advancements in the chemistry of Diels–Alder reactions for total synthesis from 2020 to 2023.

## 2. Normal Diels–Alder (DA) reaction

### 2.1. Mulberry Diels–Alder type adducts (MDAA) kuwanons G and H synthesis

**Scheme 2:**
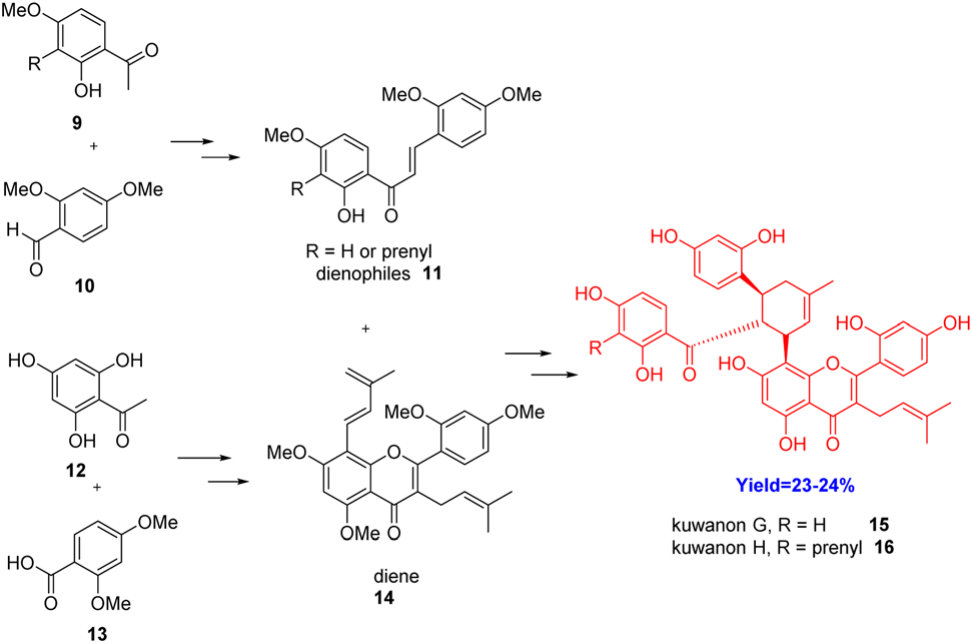
Diels–Alder reaction between 11 and 14 in the total synthesis of kuwanon G 15 and kuwanon H 16.^30^

MDAAs are a group of rare natural polyphenols found in mulberry trees, possessing promising several biological properties like anti-inflammation, anti-bacterial, anti-virus, anti-oxidation, and anti-phlogistic activities.^25–29^ Kuwanons G 15 and H 16 fall in the 4^th^ type of MDAA *i.e.* dehydroprenyl flavonoid type. Moreover, kuwanons G 15 and H 16 can be potent multitargeted agents for Alzheimer's disease. These two bioactive MDAAs were totally first synthesized by Luo *et al.*^30^ in 2021 (Scheme 2). These can be derived from simple [4+2] cycloaddition of chalcone dienophiles 11 and dehydroprenylphenol dienes 14.

### 2.2. Total synthesis of tetrodotoxin

**Scheme 3:**
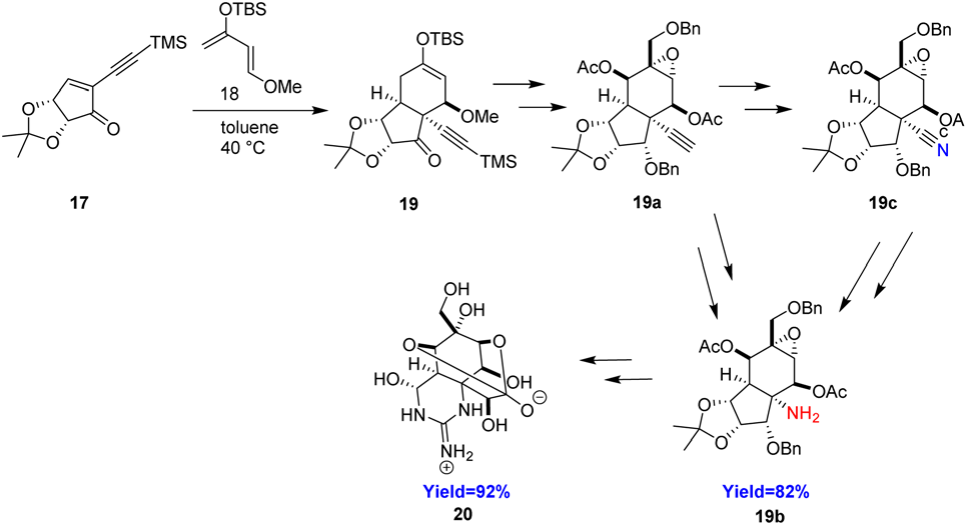
Total synthesis of tetrodotoxin 20.^31^

Tetrodotoxin (20) a well-known natural product found in puffer fish has immense biological properties. It is also known as sodium channel blocker and used to relieve the headache associated with heroin withdrawal. It contains a cyclic guanidine, hemiaminal and *ortho*-ester moieties making this molecule unique. Murakami *et al.*^31^ took 17 and siloxydiene 18 as Diels–Alder substrate for the formation of *cis*-fused 5/5/6-tricyclic compound 19 in a quantitative yield (Scheme 3). At first, the alkyne moiety was transformed to an amine group 19b through a Curtius rearrangement reaction in four steps. Murakami *et al.* also transforms alkyne moity to a nitrile group 19c by CuI, TMSN_3_ and then Hofmann rearrangement to give the amine 19b in two more steps. Subsequently, cyclic guanidine moiety was constructed followed by cyclic hemiacetal group. The resulted compound was further converted to the target molecule tetrodotoxin 20 in several steps.^31^

### 2.3. Synthesis of *para*-hydroquinone (*p*-HQ) motif and total synthesis of (±)-indanostatin

**Scheme 4:**
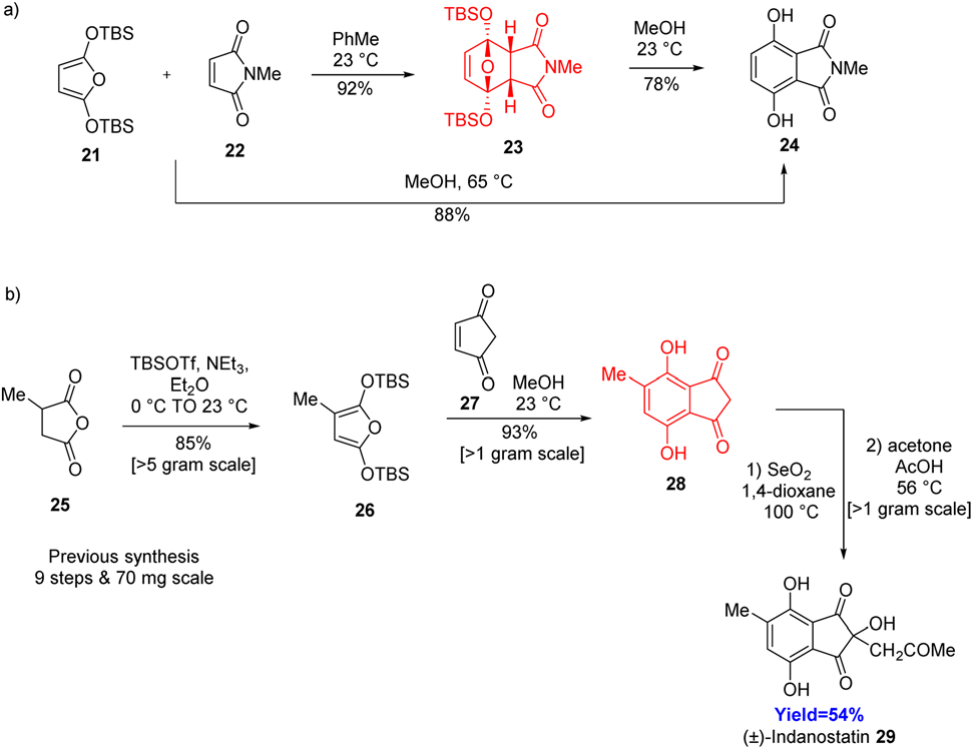
(a) Diels–Alder reaction of furan 21 and *N*-methylmaleimide (NMM) 22 in two ways to form *p*-HQs. (b) Gram scale synthesis of (±)-indanostatin 29.^32^

Many natural products, potential drug molecules composed of *p*-hydroquinone (*p*-HQ) 17 core structure. Among them, indanostatin, a natural product found in *Streptomyces* sp. which exhibits neuroprotective activity.^32^ Dissanayake *et al.*^32^ have taken 2,5-difunctionalized furans as bisketene equivalent 21, which form *p*-HQs *via* D–A/ring-opening/tautomerization sequence (Scheme 4a).^32^ The ring opening and tautomerization step can be done by simply stirring the compound in MeOH. Moreover, by performing the Diels–Alder reaction in MeOH, Dissanayake and the co-workers formed *p*-HQ in a one pot Diels–Alder/ring opening/tautomerization cascade. Same methodology was also applied in natural product synthesis, where methylfuran was opted as diene 26, 1,3-cyclopentenedione 27 as dienophile and produced (±)-indanostatin 29 in only three steps (Scheme 4b).^32^

### 2.4. Total synthesis of canataxpropellane

Canataxpropellane, a taxane skeleton, belongs to the taxane diterpene family which were isolated from plants of the genus *Taxus*. Anti-cancer^33^ drugs such as Taxol and Taxotere also belong to this family.

**Scheme 5:**
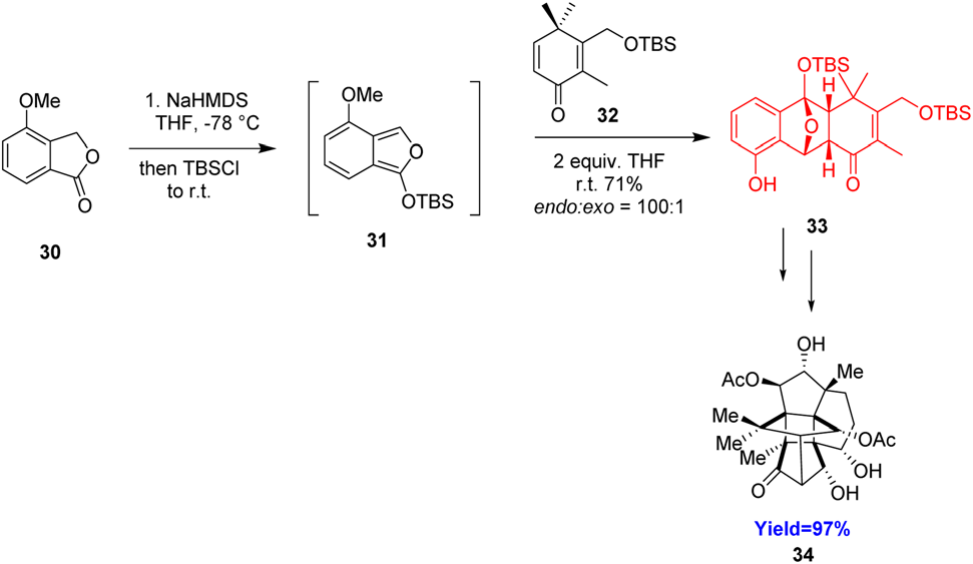
Racemic synthesis of canataxpropellane 34.^34^

**Scheme 6:**
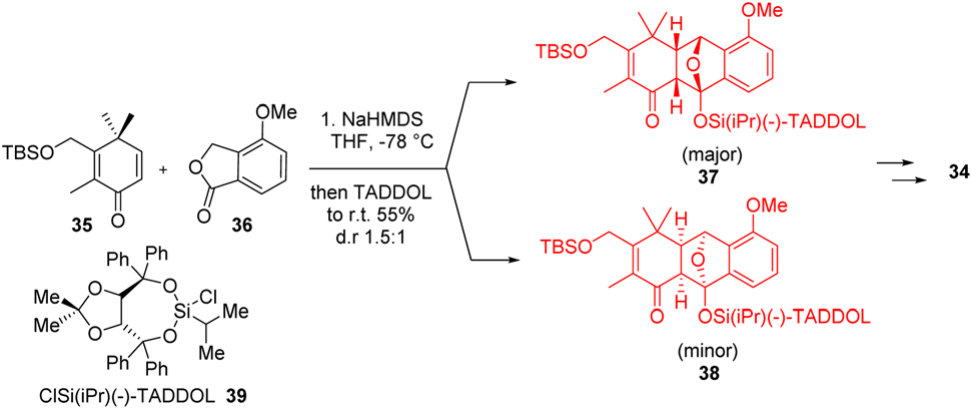
Enantiomeric synthesis of canataxpropellane 34.^34^

Canataxpropellane, a very complex natural product having heptacyclic carbon framework with two propellanes[3.3.2]propellane and [4.4.2]propellane simultaneously. Schneider *et al.*^34^ completed the total synthesis of (−)-canataxpropellane 34 in 26 steps from simple and easily accessible compounds. The starting material lactone 30 formed the diene 31 which underwent Diels–Alder reaction with dienophile 32 and showed excellent diastereoselectivity (*endo* : *exo* = 100 : 1) in racemic synthesis (Scheme 5).^34^ In enantioselective synthesis (Scheme 6)^34^ of this compound, a chiral silyl group TADDOL catalyst 39 was used where the diastereoselectivity (*endo* : *exo* = 1.5 : 1) was moderate. But the advantage is that the products can be readily separated by column chromatography. The Diels–Alder reaction and the *ortho*-alkene–arene photocycloaddition sequence formed the core structure of canataxpropellane which was further converted to the target molecule canataxpropellane in several steps.^34^

### 2.5. Total synthesis of kingianin F

**Scheme 7:**
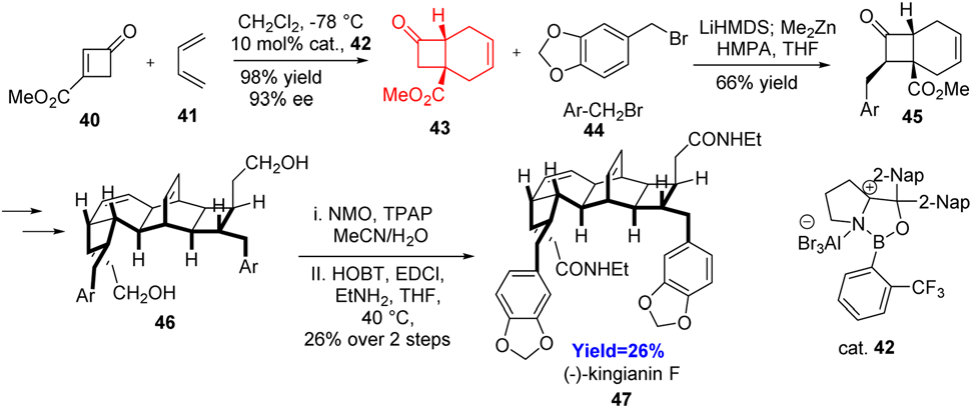
Total synthesis of kingianin F (47).^35^

Yan and co-workers^35^ in 2021 developed a method where cyclobutenone acts as a reactive dienophile and further used this method in the total synthesis of kingianin F 47. Kingianins A–N, having significant binding affinity for the protein Bcl-X_L_,^36^ belongs to a family of bicyclo[4.2.0]octadiene dimers. These bicyclo[4.2.0]octane motifs are present in several natural products. Yan *et al.* synthesised bicyclo[4.2.0]octane motifs 43 using Diels–Alder reaction between highly reactive cyclobutenone having better stability and diene 41 (Scheme 7). Their work performed best when they have used 3-(methoxycarbonyl)cyclobutenone 40 in the presence of chiral oxaborolidinium ions (COBI) 42. The formation of bicyclo[4.2.0]octane scaffold is the key step in the total synthesis of kingianin F 47. After forming this scaffold, kingianin F was generated in 12 steps only.^35^

### 2.6. Total syntheses of massarinolin A, purpurolides B, D, E; 2,3-deoxypurpurolide C and structural revision of massarinolin A

**Scheme 8:**
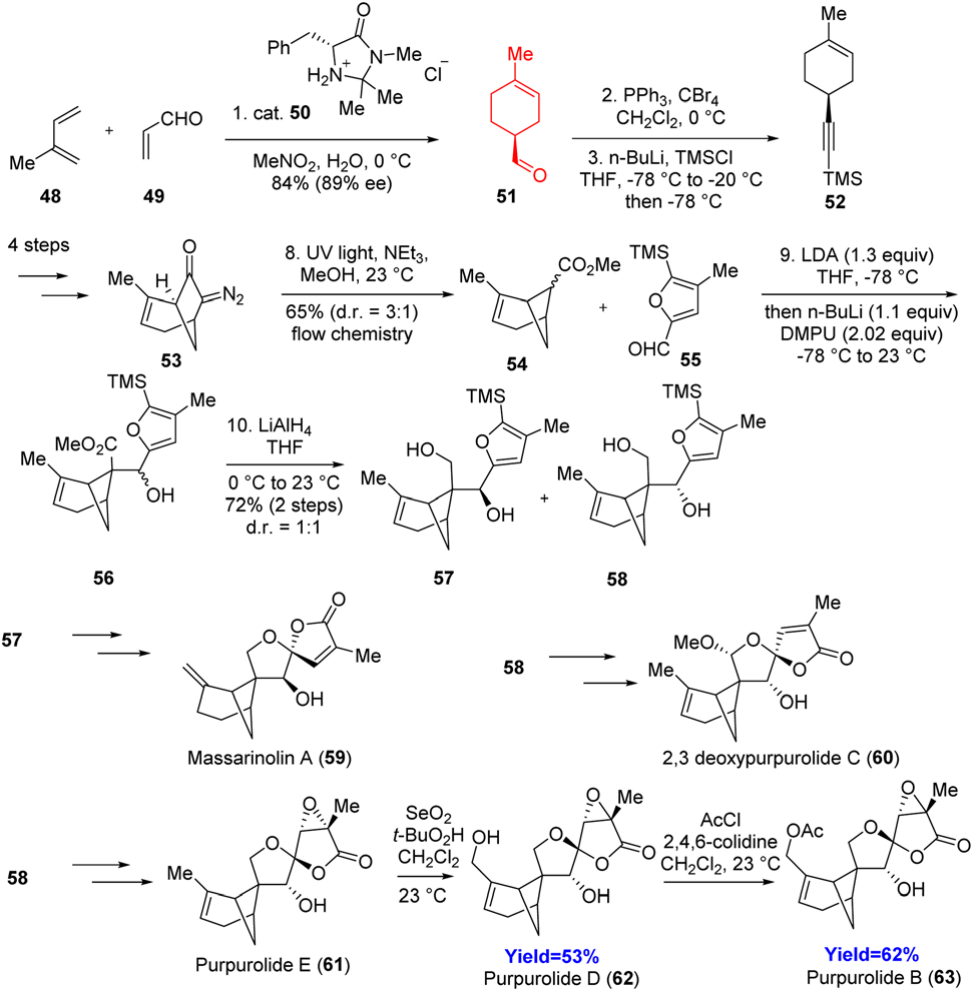
Total Syntheses of massarinolin A, purpurolides B, D, E; 2,3-deoxypurpurolide C.^37^

Massarinolin A, purpurolides belong to the bioactive bergamotane sesquiterpenes. These compounds have a complex ring system consisting of a bicyclo[3.1.1]heptane, oxaspiro[3.4]octane, and dioxaspiro[4.4]nonane (oxaspirolactone). Massarinolin A shows activity against Gram-positive bacteria whereas purpurolides D, E, and F showed significant inhibitory activity against anti-obesity drug target pancreatic lipase. Wang and co-workers^37^ started with a Diels–Alder reaction with chiral amine catalyst, and the adduct 51 underwent Corey–Fuchs homologation to form 52 (Scheme 8).^37^ Flow photochemical Wolff rearrangement of 53 had been used to produce the key intermediate 54. Aldol reaction with 1.3 equiv. of LDA followed by reduction produced the precursors 57 and 58 of these natural products. Photochemical oxidative furan cyclization with tetraphenylporphyrin as catalyst formed the oxaspirolactone moiety in separable mixture form. For the purpurolides, similar procedure has been done and in both the cases mixture has been produced where the *trans*-product is thermodynamically more stable. That *trans*-product was converted to the massarinolin A 59, and purpurolides D, E and B 61–63. Consequently, Wang *et al.* demonstrated that massarinolin A is also a *trans* compound.^37^

### 2.7. Unified total syntheses of diterpenoids rhamnofolane, tigliane, and daphnane

**Scheme 9:**
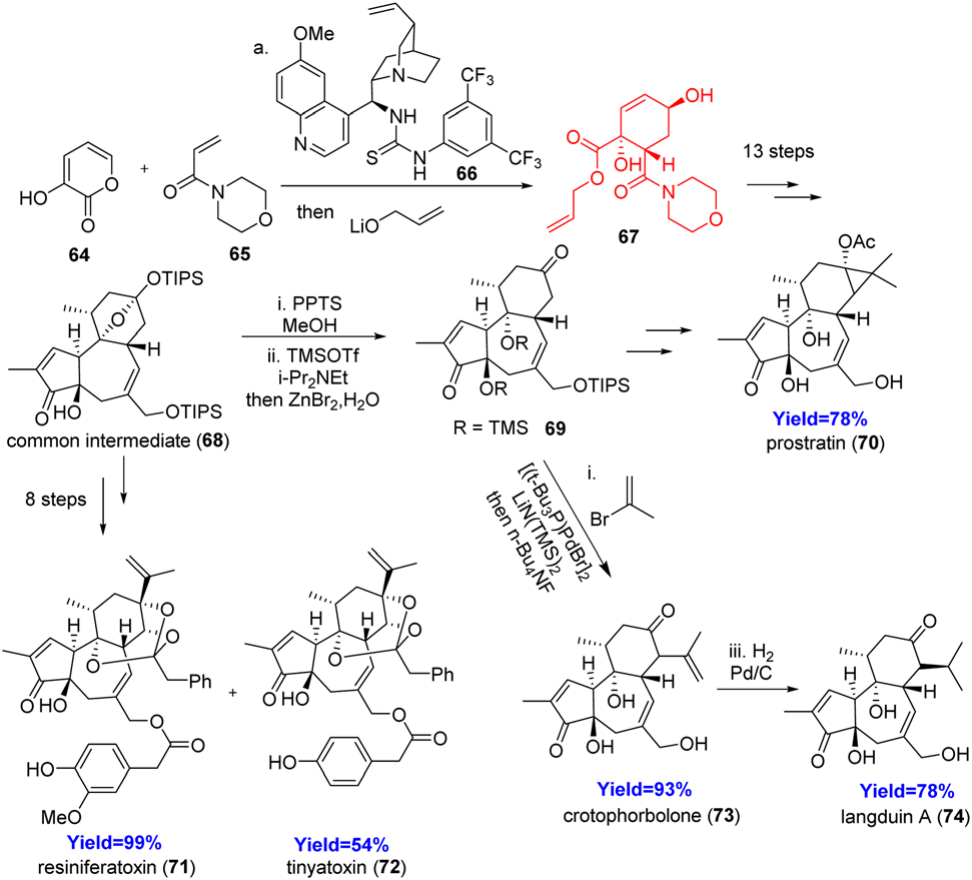
Total syntheses of rhamnofolane, tigliane, and daphnane diterpenoids.^39^

Rhamnofolane, tigliane and daphnane are three families of diterpenoids which have closely related biosynthetic origins. Various biological activities like anti-viral, anti-cancer, analgesic, immunomodulatory, neuro-trophic, and tumor promotional activities are observed by these families of diterpenoids.^38^ They have same 5/7/6-*trans* fused ring system only differing in the substitutions at the C13, C14 positions. Hirose and the co-workers^39^ synthesised crotophorbolone/langduin A, prostratin, and resiniferatoxin/tinyatoxin which belong to the rhamnofolane, tigliane, and daphnane families, respectively. They have prepared a common intermediate which diverged into the targets. At first an asymmetric Diels–Alder reaction was done between 64 and 65 using a quinine derived catalyst 66 to prepare the six membered ring (Scheme 9). The resulting adduct 67 converted to the common intermediate 68 in thirteen steps. After forming the common intermediate 68, the C13 cyclic acetal was converted to C13 ketone in two steps for the functionalization of C14 position. The formed product 69 could be converted to prostratin 70 and crotophorbolone 73 which on hydrogenation produced langduin A 74. Resiniferatoxin 71 and tinyatoxin 72 was synthesized from the common intermediate 68.^39^

### 2.8. Synthesis of spirocyclic core of 13-desmethyl spirolide C

**Scheme 10:**
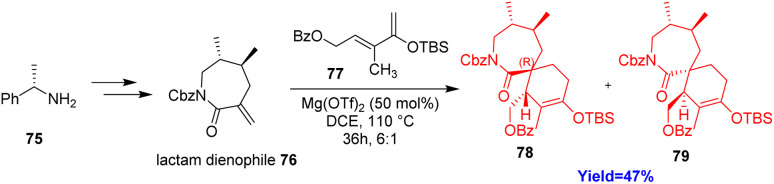
Synthesis of spirocyclic core of 13-desmethyl spirolide C.^40^

13-Desmethyl spirolide C having [7,6]spirocyclic imine moiety has the potential to work against Alzheimer's disease. Earl and co-workers^40^ developed an economical, robust way to synthesize spirocyclic core through a key aza-Claisen rearrangement and *exo*-selective Diels–Alder reaction. At first aza-Claisen precursor amide was prepared from (S)-(−)-α-methylbenzylamine 75 and then the amide was converted to enantiopure lactam dienophile 76 (Scheme 10). The lactam dienophile reacted with the diene 77 (containing benzoyl protected side chain) and produced the spirocyclic fragment 78, 79 with complete *exo*-selectivity.^40^

### 2.9. Synthesis of azulene cross-conjugated phthalimides

**Scheme 11:**
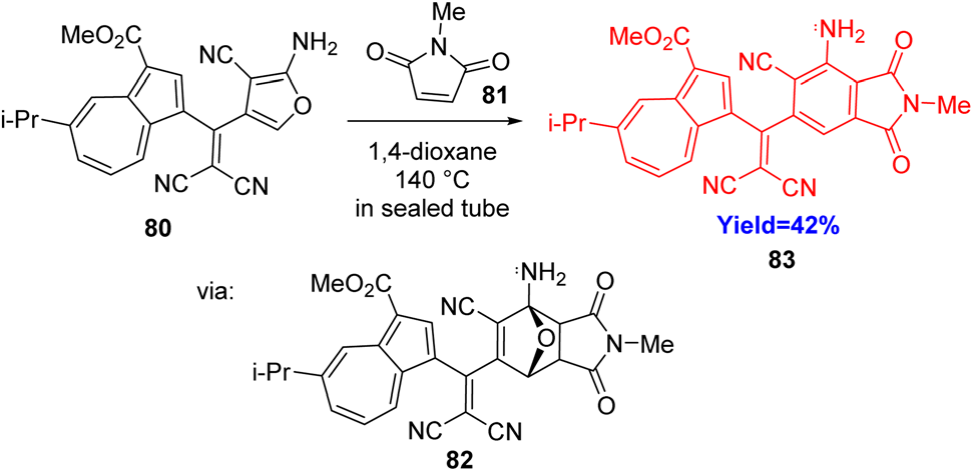
Synthesis of azulene cross-conjugated phthalimides.^41^

Phthalimides and their derivatives are largely found in bioactive compounds and can also be used as organic electronics and organic semiconductors. Shoji *et al.*^41^ coupled azulene-2-aminofurans 81 and maleimide derivatives 82*via* an *endo*-selective Diels–Alder reaction. At first, [4+2] cycloaddition took place to form the bridged hemi-aminals 82, and then electron donation of the germinal amino group helped to cleave the bridged C–O bond (Scheme 11). After the ring opening isomerization, tautomerization and rearomatization the azulene cross-conjugated phthalimide 83 have been produced.^41^

### 2.10. Total synthesis of senepodine F

**Scheme 12:**
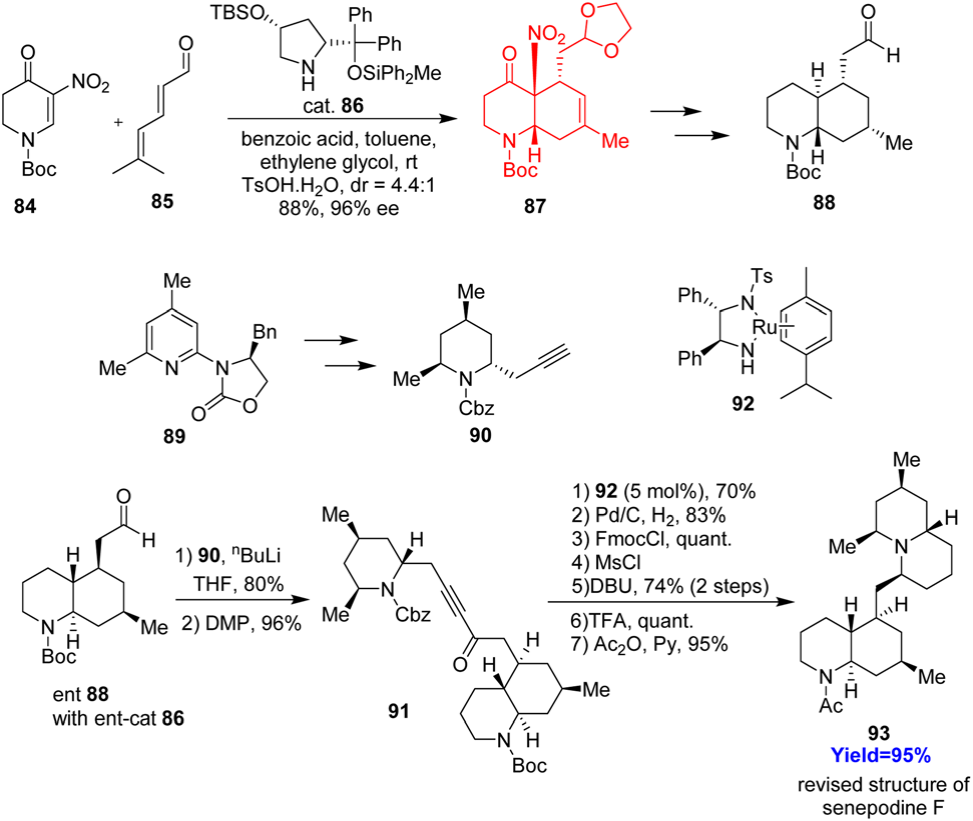
Total synthesis of senepodine F (93).^42^

Senepodine F, containing a decahydroquinoline ring (AB) and a quinolizidine ring (CD) connected by a methylene tether, isolated from *lycopodium* plants. Some *lycopodium* alkaloids are known for acetylcholinesterase inhibition. This is the first time ever senepodine F has been synthesized which is done by Nakashima *et al.*^42^ The decahydroquinoline ring (AB) was constructed *via* asymmetric Diels–Alder reaction between 5-nitro-2,3-dihydropyridone derivative 84 and a α,β,γ,δ-unsaturated aldehyde 85 followed by denitration/isomerization (Scheme 12). After preparing the C ring, from 2-oxazolidinone substituted pyridine derivative 89, it was coupled with AB ring 88. Noyori asymmetric reduction and intramolecular S_N_^2^ cyclization formed the D ring and at last senepodine F 93.^42^

### 2.11. Total syntheses of rhodomollins A and B

**Scheme 13:**
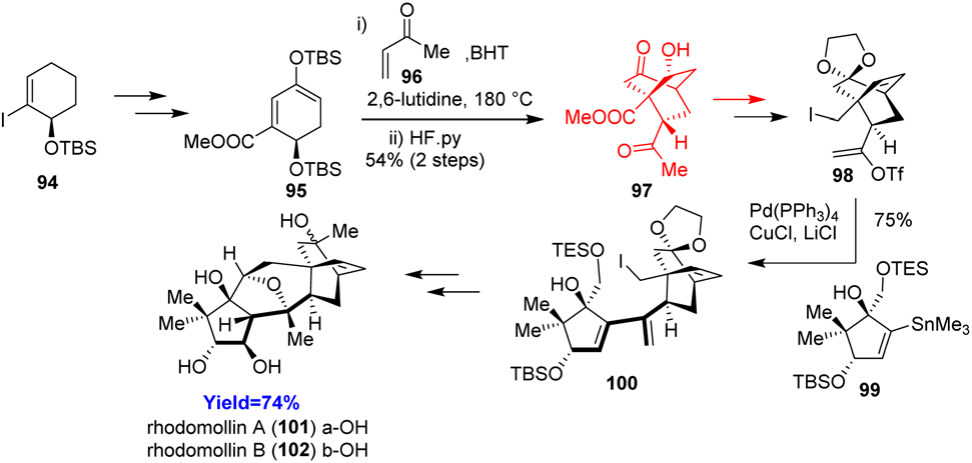
Total syntheses of rhodomollins A (101) and B (102).^43^

Rhodomollins A and B have a complex structure having five rings (ABCDE) connected with each other. Zhao *et al.* have developed first ever total syntheses of rhodomollins A and B, two graynoids having oxa-bicyclo[3.2.1] core (BC ring). According to their retrosynthetic analysis, Zhao and co-workers^43^ commenced the total synthesis by constructing A and DE ring separately. Diels–Alder reaction was needed during the preparation of DE ring between a diene 95 and methyl vinyl ketone 96 in the presence of 2,6-lutidine as buffer and BHT as an anti-oxidant (Scheme 13). The A and DE ring was merged by Stille coupling and the coupled product converted to rhodomollins 101, 102 in more than 10 steps through lithium–halogen exchange/Williamson ether synthesis and Payne–Meinwald rearrangement.^43^

### 2.12. Synthesis of tricyclic core of cyclocalopin A

**Scheme 14:**
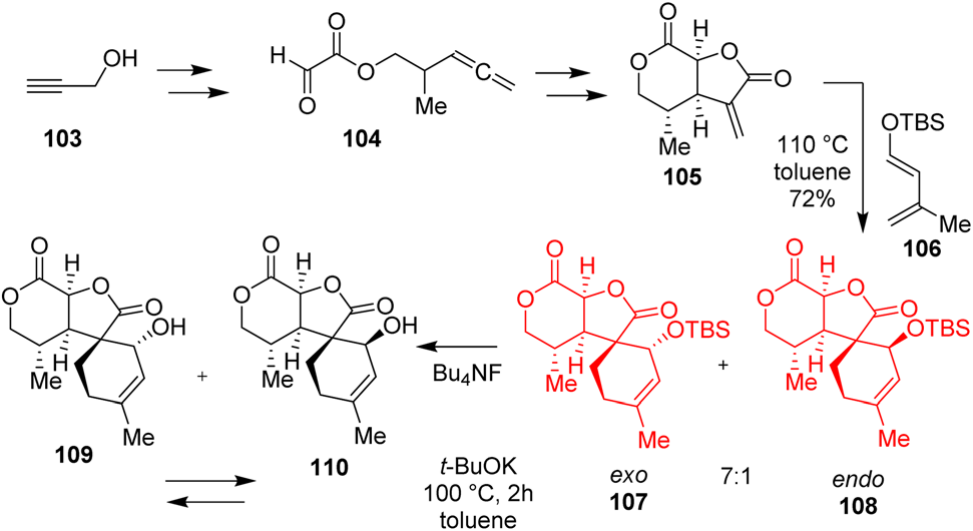
Synthesis of tricyclic core of cyclocalopin A.^45^

Several mushrooms such as *Boletus calopus*, *Caloboletus radicans* are the source of cyclocalopin family. Cyclocalopin A, comprised of a fused and spiro-ring, showed free radical scavenging activity as well as anti-cancer activities.^44^ Yu *et al.*^45^ based on their retrosynthesis began to prepare allenyl glyoxylate 104 from 103 through Fischer esterification (Scheme 14). The allenyl glyoxylate 104 on two [2+2+1] cyclocarbonylation constructed the dienophile α-methylene bis-γ,δ-lactone 105. The Diels–Alder reaction, performed at 110 °C for 15 h in toluene, produced the major component 107 with *exo*-selectivity and minor component 108 with *endo*-selectivity. In the equilibrium-separation process the *exo*-product 109 was interconvertible to the *endo* one 110 and the tricyclic core of cyclocalopin A was formed.^45^

### 2.13. Synthesis of tetrahydrocarbazole motif *via* vinylindole

**Scheme 15:**
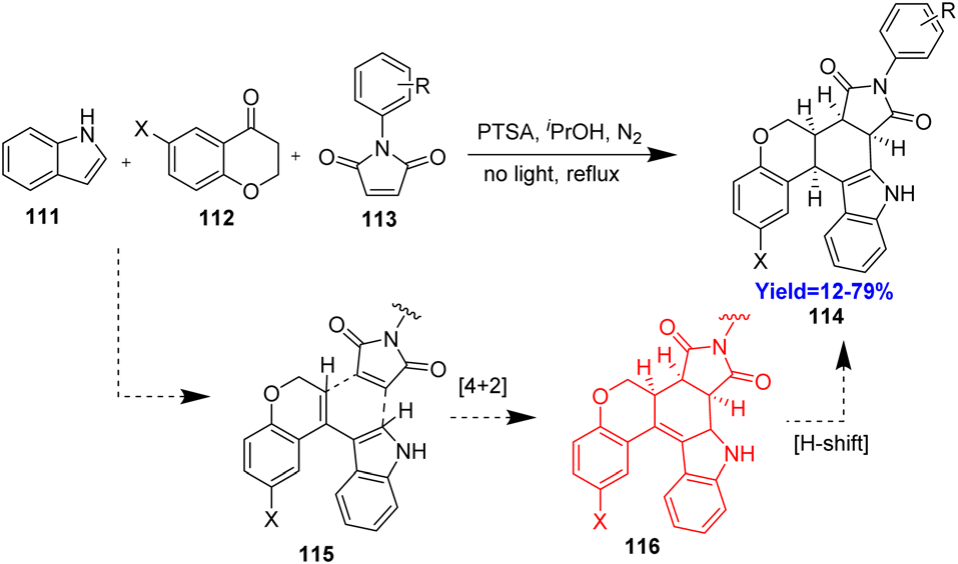
Synthesis of tetrahydrocarbazole motif (114) *via* vinylindole (115).^47^

Tetrahydrocarbazole motifs are largely found in natural products and bioactive molecules.^46^ Moreover, they are found to be a precursor or an intermediate in the formation of various complex natural products. Noland and Abzhabarov^47^ had developed a one pot three component reaction for the synthesis of tetrahydrocarbazole (Scheme 15). They have taken indole 111, 1-tetralone 112, *N*-phenylmaleimide 113 in a pot where the indole 111 and 1-tetralone 112 reacted to form a vinylindole 115*in situ*. Then vinylindole 115 and *N*-phenylmaleimide 113 underwent normal Diels–Alder reaction to generate the tetrahydrocarbazole motif 114.^47^

### 2.14. Total synthesis of euonymine and euonyminol octaacetate

**Scheme 16:**
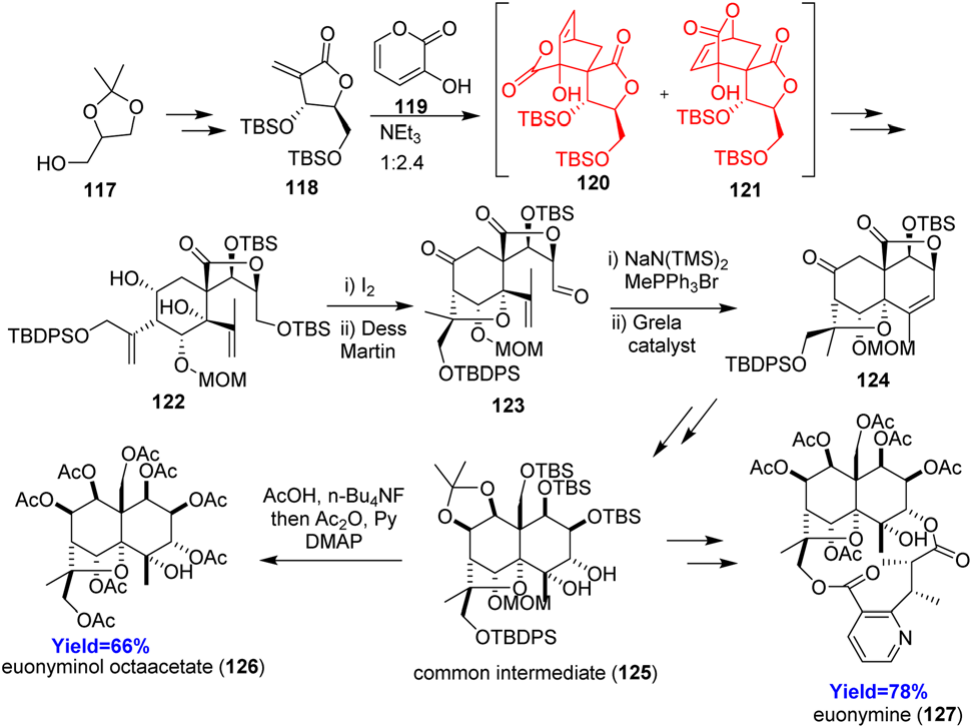
Total synthesis of euonymine (127) and euonyminol octaacetate (126).^48^

Euonymine and euonyminol octaacetate, members of dihydro-β-agarofuran family, shows anti-HIV and P-glycoprotein inhibitory effects.^48^ Wang and co-workers^48^ constructed euonymine and euonyminol octaacetate in 29 and 24 steps respectively from (*R*)-glycerol acetonide 117 (Scheme 16). These two compounds have ABC ring, where the B ring was prepared through Morita–Baylis–Hillman reaction followed by Diels–Alder reaction. The Diels–Alder adducts 120, 121 converted to 122, which upon iodo etherification produced the C-ring 123 and the A-ring 124 was formed by ring-closing olefin metathesis. After forming the core ABC ring, the common intermediate 125 was formed which can form euonyminol octaacetate 126 in 1 step and euonymine 127 in several steps.^48^

### 2.15. Synthesis of tetracyclic core of calyciphylline N

**Scheme 17:**
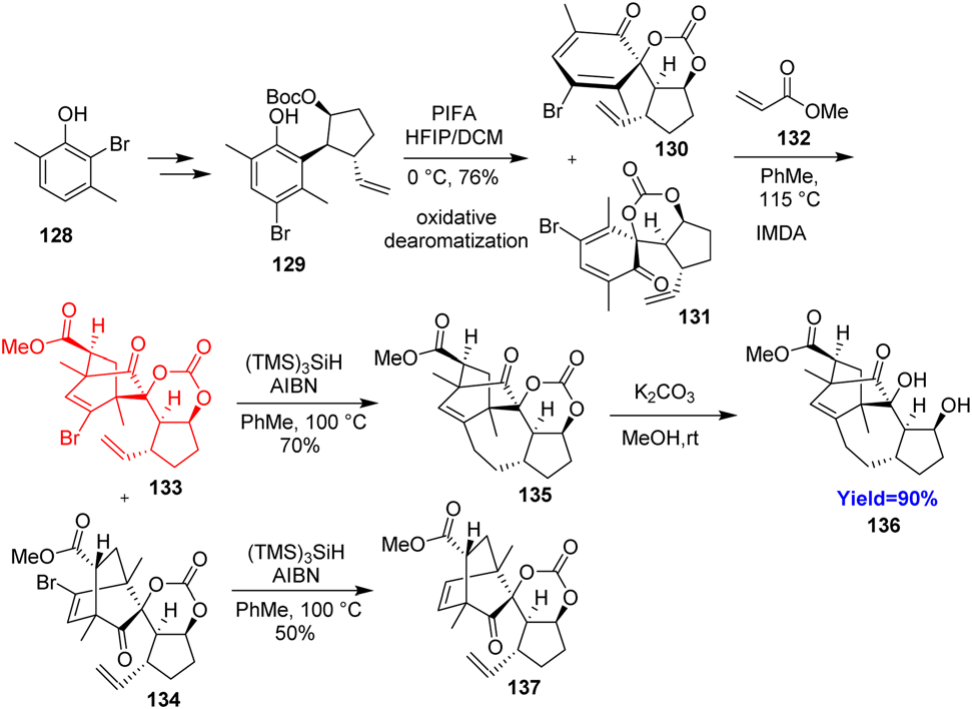
Synthesis of tetracyclic core of calyciphylline N (136).^49^

Calyciphylline N, belongs to the daphniphyllum alkaloids, possess a unique bicyclo[2.2.2]octane core, dihydropyrrole, and a tricyclic dodecahydrocyclopenta[*cd*]azulene. Yumeng Lv and co-workers^49^ started from *p*-xylenol to 128 which converted to the key intermediate 129 (Scheme 17). The bicyclo[2.2.2]octane motif present in 133 and 134 was prepared by the usual oxidative dearomatization/intermolecular Diels–Alder reaction cascade. At last intramolecular radical cyclization of 133 constructed the [6.6.7.5] tetracyclic core of calyciphylline N 136*via*135 while 134 produced uncyclized 137.^49^

### 2.16. Total synthesis of some spirocyclane containing natural products

**Scheme 18:**
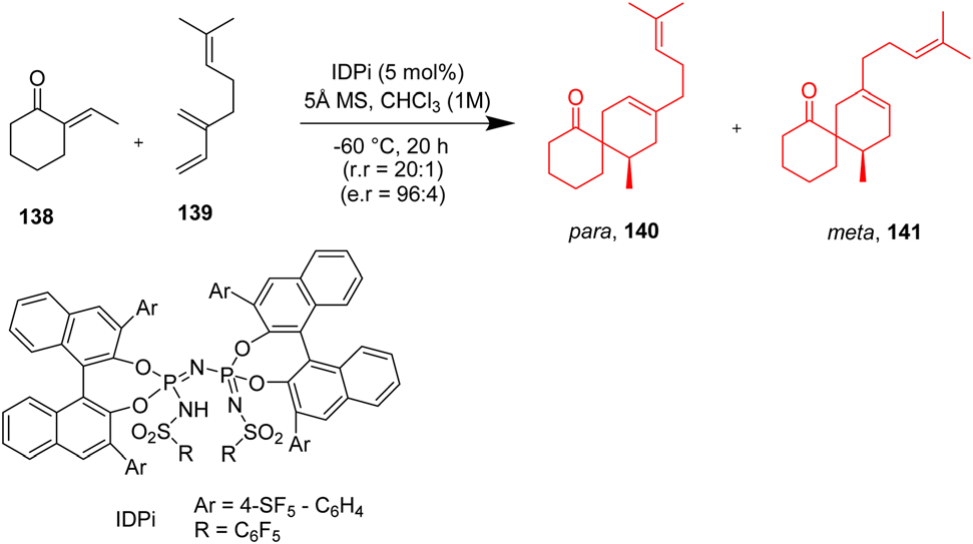
Enantioselective intermolecular Diels–Alder reaction to form spirocyclanes (140, 141).^50^

Enantioselective synthesis of spirocyclanes maintaining the step-economic and atom economic route remains an interesting problem to the chemists. The skeletons of spiro[4.5]decane and spiro[5.5]undecane skeletons have attracted the chemists, since several biologically active sesquiterpenoids comprise these. Catalytic enantioselective spirocyclization Diels–Alder reactions between *exo*-enones and dienes are unprecedented. Strongly acidic and confined imidodiphosphorimidate (IDPi) catalyst is used to catalyse these reactions. It was proposed by Santanu Ghosh^50^ and his coworkers that these acids may also be used to protonate such *exo*-enone. Thus Ghosh *et al.*^50^ have showed an effective intermolecular Diels–Alder reaction of diversely substituted *exo*-enones 138 with various dienes 139 that is enantioselective and catalyzed by Brønsted acid, which aids in the synthesis of enantiopure spirocarbocyclic scaffolds 140, 141 of bioactive sesquiterpene natural products (Scheme 18).^50^ Excellent yield, enantio and regioselectivities are obtained because the regio and stereochemical outcomes are controlled by the high acidity and confined chiral microenvironment of the IDPi catalyst.

**Scheme 19:**
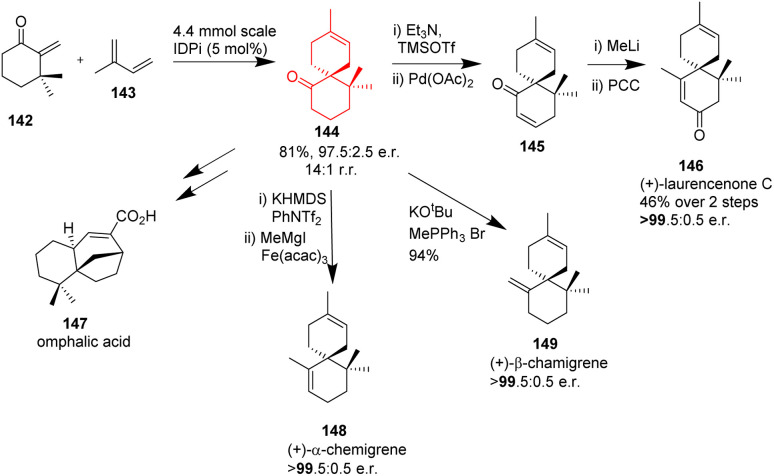
Total syntheses of various spirocycle containing natural products.^50^

These reactions were applicable in forming various natural products like (+)-β-chamigrene 149, (+)-α-chamigrene 148, omphalic acid 147, and (+)-laurencenone C 146 from a common intermediate 144 (Scheme 19).^50^

### 2.17. Total synthesis of atrachinenin A and B

**Scheme 20:**
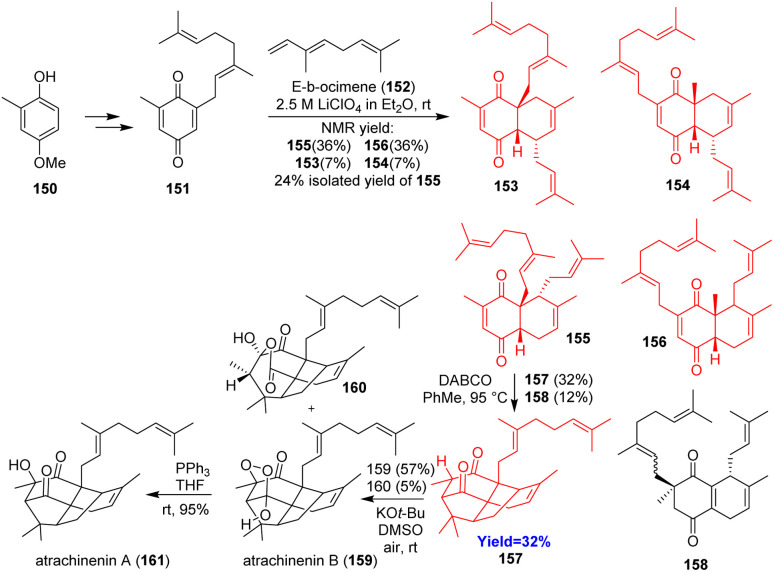
Total synthesis of atrachinenin A (161) and B (159).^51^

Atrachinenin A and B were isolated from the rhizomes of Chinese medicinal plant *Atractylodes chinesis*. French *et al.*^51^ commenced the total synthesis with geranylation of 4-methoxy-2-methylphenol 150 followed by oxidation and it led to quinone 151 (Scheme 20). The quinone 151 underwent Diels–Alder reaction with *E*-β-ocimene 152 to give four possible *endo* products. The desired product 155 was purified and produced 157 on [3+2] cycloaddition. 157 on aerobic oxidation in DMSO produced atrachinenin B 159 which on reduction gave atrachinenin A 161.^51^

### 2.18. Synthesis of terpinen-4-ol

**Scheme 21:**
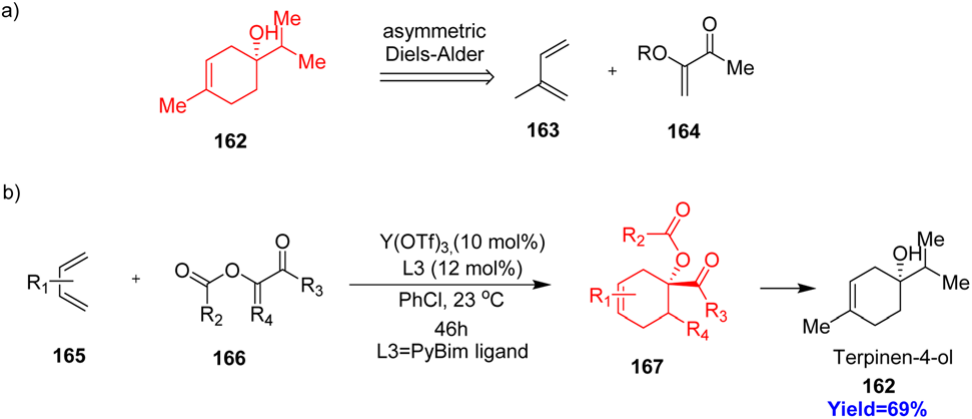
(a) Retrosynthetic disconnection of terpinen-4-ol (162), (b) Y-catalysed asymmetric DAR to construct (162).^52^

Naturally occurring monocyclic cyclohexene containing monoterpenes are important chiral building blocks for the synthesis of bioactive compounds, medicines, and agrochemicals. In the synthesis of commercial herbicide cinmethylin, a key intermediate terpinen-4-ol 162, also a naturally occurring monoterpene. Retrosynthetic disconnection of 162 showed that it can be made from isoprene unit 163 (Scheme 21a).^52^ Mendoza *et al.*^52^ in 2022, reported Y-catalyzed asymmetric DAR of α-acyloxy enones 166 with diene 165 to form the terpinen-4-ol 162 (Scheme 21b). Generally these reactions have good enantio, diastereo, as well as regioselectivity.^52^

### 2.19. Total synthesis of cephanolides A–D

**Scheme 22:**
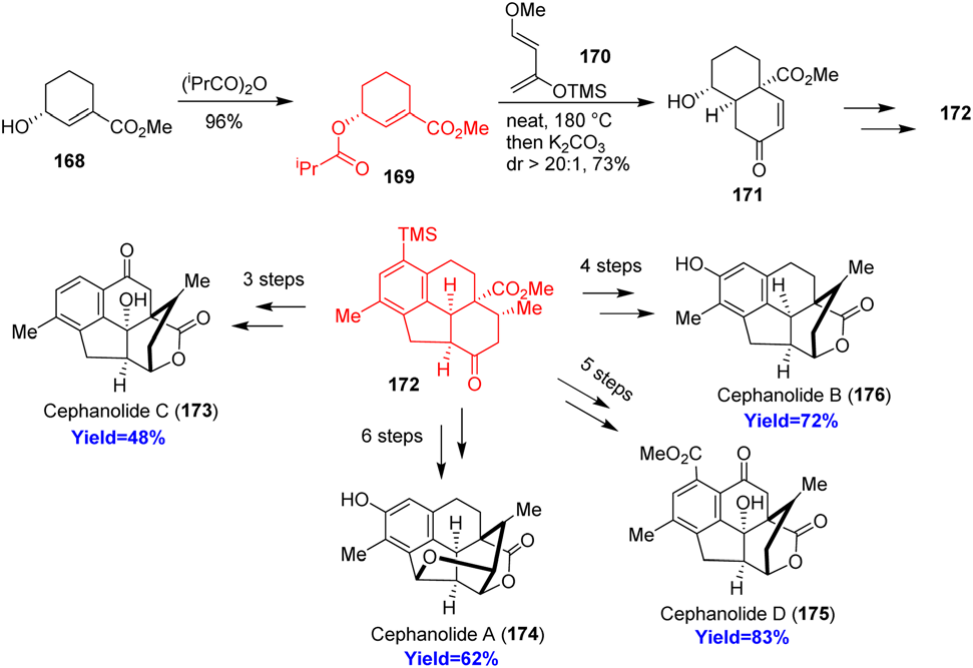
Total synthesis of cephanolides A–D.^53^

Cephalotane-type diterpenoids cephanolides A–D, isolated from *Cephalotaxus sinesis*, have 6/6/6/5 tetracyclic core with a bridged δ-lactone. According to the retrosynthetic analysis, Qing *et al.*^53^ began the synthesis with alcohol 168 which on acylation formed the dienophile 169 (Scheme 22). This dienophile 169 and Danishefsky's diene 170 on Diels–Alder reaction produced 171 with high diastereoselectivity (dr = 20 : 1). The cycloadduct 171 was converted to common intermediate 172 through a palladium catalyzed [2+2+2] cycloaddition. The common intermediate (172) synthesized cephanolide A 174 in 6 steps and other cephanolides 173, 175, 176 in 4–5 steps.^53^

### 2.20. Total synthesis of trichloranoid C and trishizukaol A

**Scheme 23:**
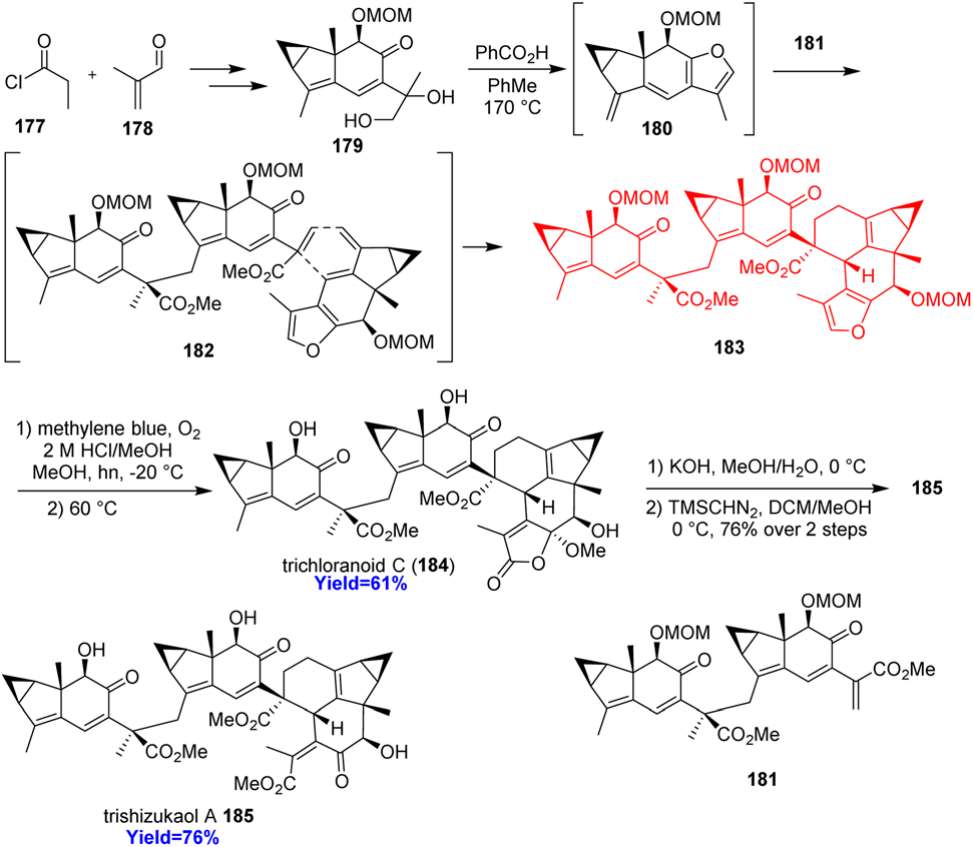
Total synthesis of trichloranoid C (184) and trishizukaol A (185).^55^

Trichloranoid C and trishizukaol A are two oligomeric lindenane sesquiterpenoids from *Chloranthus* genus having several biological properties.^54^ Wang *et al.*^55^ commenced the total synthesis by assembling propionyl chloride 177 and methacrolein 178 (Scheme 23). The substrates were converted to 179 in ten steps which produced the diene 180 in presence of benzoic acid in toluene. The Diels–Alder adduct 183, produced by diene 180 and dienophile 181, underwent dearomatization of furan and formed lactone sequentially. Deprotection of that lactone produced trichloranoid C 184 which in two steps formed trishizukaol A 185.^55^

### 2.21. Total synthesis of four kopsane alkaloids

**Scheme 24:**
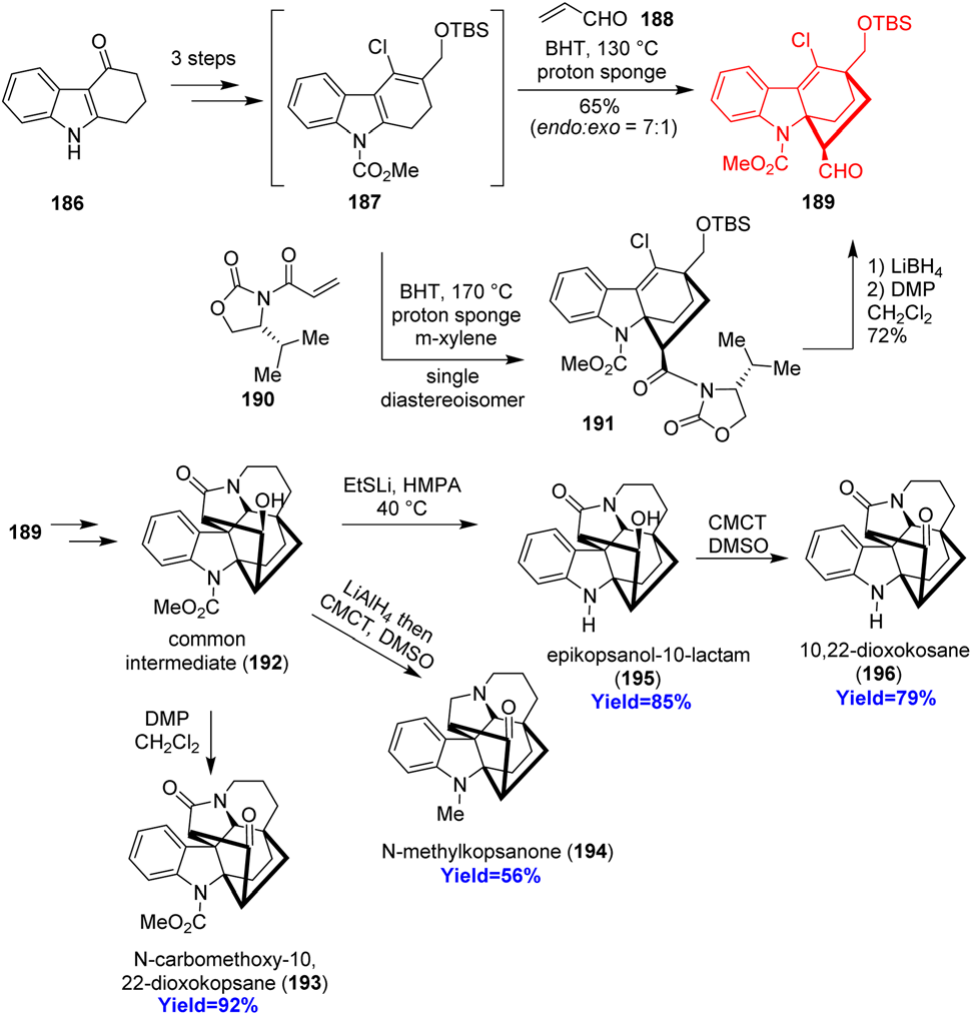
Total synthesis of four kopsane alkaloids (193, 194, 195, 196).^56^

Kopsane-type alkaloids are indole alkaloids having a complex heptacyclic skeleton. Qin and co-workers^56^ reported the total synthesis of 22-dioxokopsane, 10,22-dioxokopsane, epikopsanol-10-lactam, *N*-carbomethoxy-10 and *N*-methylkopsanone. According to the retrosynthetic analysis, commercially available tetrahydrocarbazolone 186 was used to prepare the Diels–Alder substrate 187*via* Vilsmeier–Haack reaction (Scheme 24). The diene 187 along with acrolein dienophile 188 underwent Diels–Alder reaction to construct the bicyclo[2.2.2]octane motif 189. Besides, the cycloadduct 189 has been synthesized *via* an enantioselective way also. The common intermediate 192 having the heptacyclic skeleton was prepared in total 16 steps and was converted to the targeted natural products.^56^

### 2.22. Total syntheses of sarbracholide and shizukaol B

**Scheme 25:**
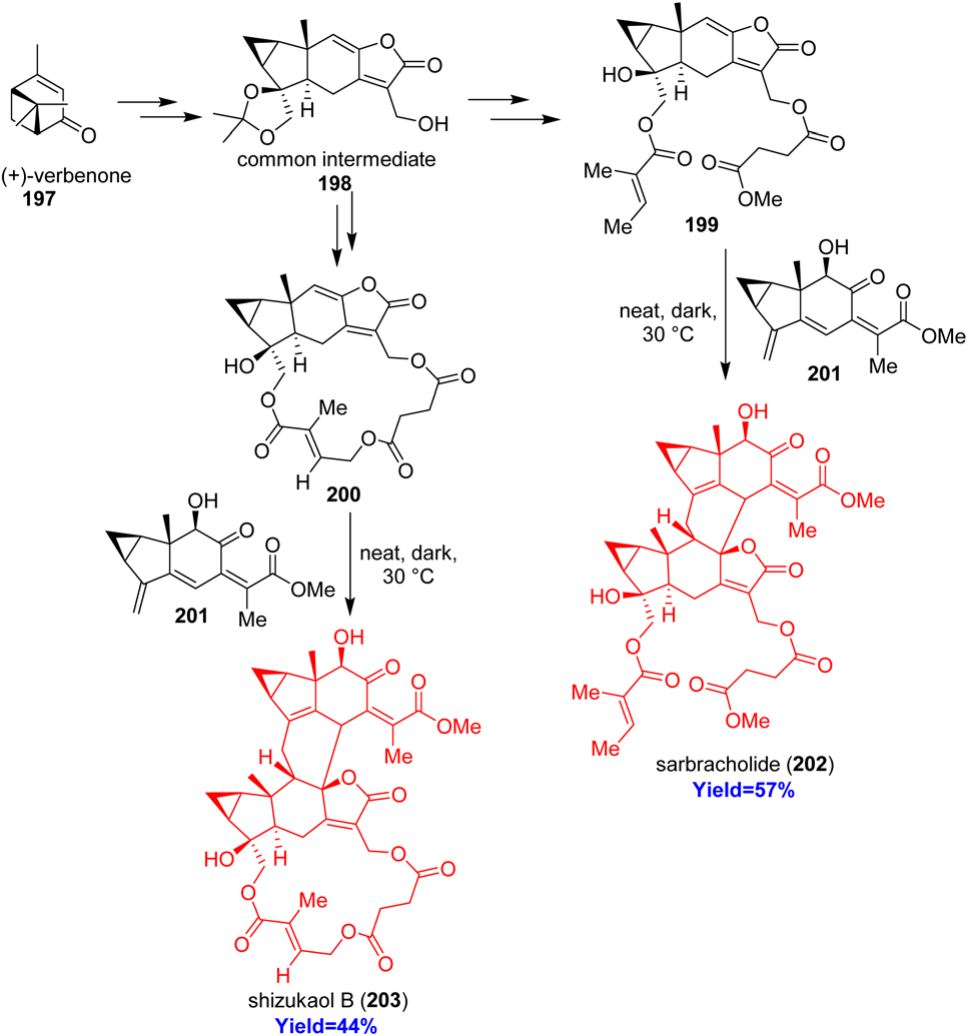
Total syntheses of sarbracholide (202) and shizukaol B (203).^57^

Lindenane sesquiterpenoid oligomers are generally dimers or trimers coupled head-to-head or head-to-back from lindenane monomer. Huang and co-workers^57^ have choosed most abundant [4+2] head to back type oligomers sarbracholide and shizukaol B for their total synthesis because of their excellent biological activities. Sarbracholide is the most potent anti-malarial agent (1000 times more than artemisinin) while shizukaol B showed excellent anti-malarial and anti-HIV-1 activities.^58–61^ Total synthesis started with (+)-verbenone 197 and formed the common intermediate 198 which can form the dienophiles 199, 200 of sarbracholide and shizukaol B respectively (Scheme 25). Then the dienophile was reacted with the common diene 201 of natural lindenane oligomers to form the desired sarbracholide 202 and shizukaol B 203.^57^

### 2.23. Total synthesis of artatrovirenol A

**Scheme 26:**
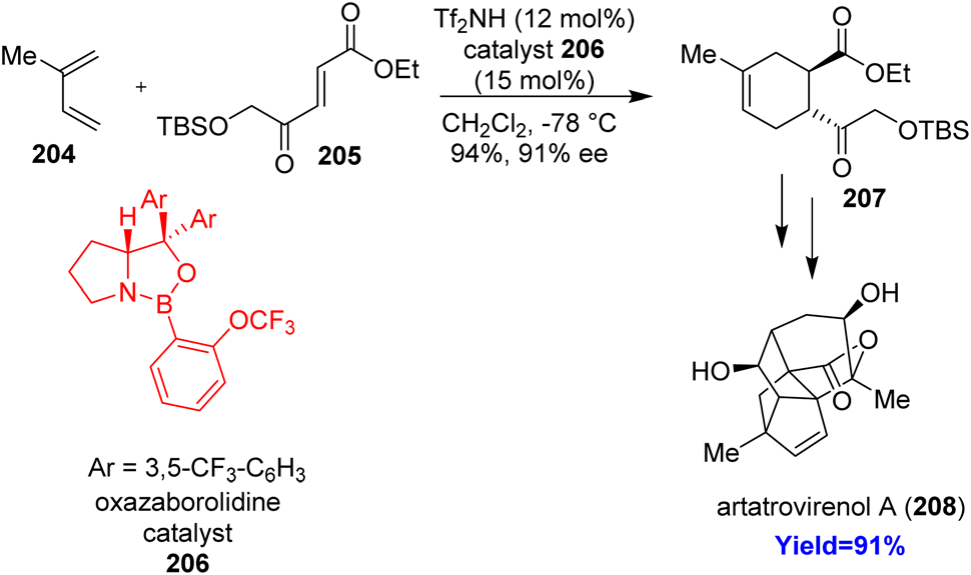
Total synthesis of artatrovirenol A (208).^63^

Sesquiterpenoid artatrovirenol A, found in traditional Chinese medicinal plant *Artemisia*, isolated from *Artemisia atroviren*. Artatrovirenol A showed its potential for preparing anti-hepatoma drug and exhibited cytotoxicity against human hepatoma cell lines.^62^ Lavernhe *et al.*^63^ started the total synthesis with a Diels–Alder reaction between diene isoprene 204 and dienophile 205 in the presence of oxazaborolidine catalyst 206 (Scheme 26). The cycloadduct 207 formed with excellent regio, diastereo and enantioselectivity transformed to the final product artatrovirenol A 208 through several steps.^63^

### 2.24. Synthesis of spiroimine fragment of portimines A and B

**Scheme 27:**
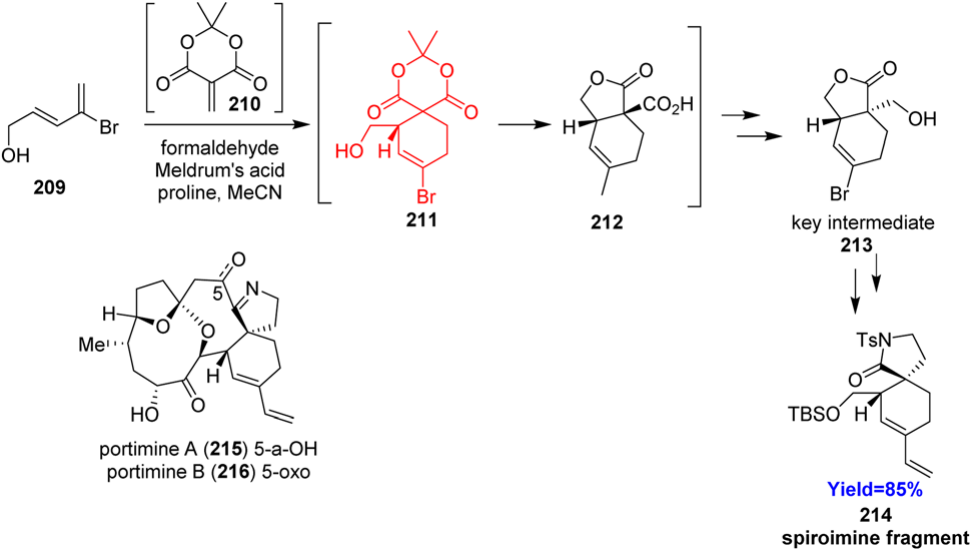
Synthesis of spiroimine fragment 214 of portimines A (215) and B (216).^66^

Though spiroimines are toxins produced by dinoflagellates, they have huge biological activities like anti-HIV, anti-cancer and anti-fouling activities.^64,65^ Portimines A and B belong to the rare [5,6]-spiroimine system. However, there are few examples of portimines total synthesis, Ding *et al.*^66^ decided to develop portimine fragment spirocyclic lactam, which could be the common intermediate for the portimines (Scheme 27). Synthesis began with a Diels–Alder reaction between bromodiene 209 and a dienophile 210 produced *in situ*. The cycloadduct 211 again cyclized *in situ* and formed lactone 212 which upon reduction, lactonization produced the key intermediate 213. The key intermediate 213 produced the desired spirocyclic lactams 214 in few steps.^66^

### 2.25. Total synthesis of sculponin U

**Scheme 28:**
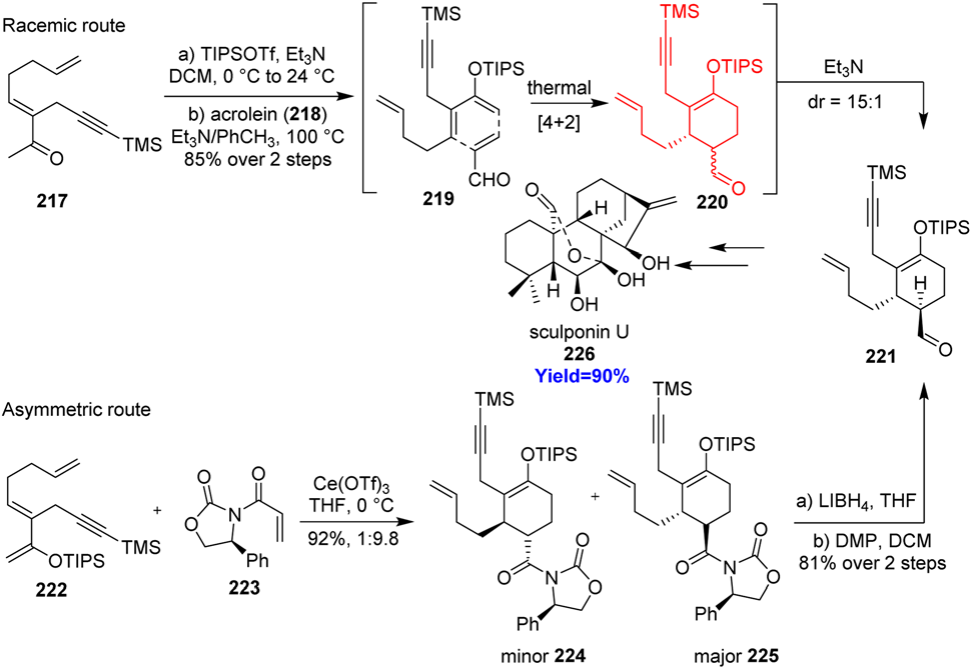
Total synthesis of sculponin U (226).^68^

Sculponin U, an *ent*-kaurane-type diterpenoid, isolated from *Isodon sculponeatus*. This family of diterpenoids showed anti-tumor, anti-bacterial properties.^67^ Cao *et al.*^68^ started the total synthesis with a Diels–Alder reaction between a silyl enolate diene and acrolein dienophile 218 forming the middle six-membered ring of sculponin U 226 (Scheme 28). Epimeric mixture of cycloadduct 220 was formed *in situ* which in presence of triethylamine produced a thermodynamically stable compound 221 with 15 : 1 dr. Finally, racemic synthesis of sculponin U 226 was done from compound 221. Cao and co-workers also showed the asymmetric way to synthesize sculponin U by preparing enantiopure form of compound 221 through Diels–Alder reaction.^68^

### 2.26. Total synthesis of neocaesalpin A and AA

**Scheme 29:**
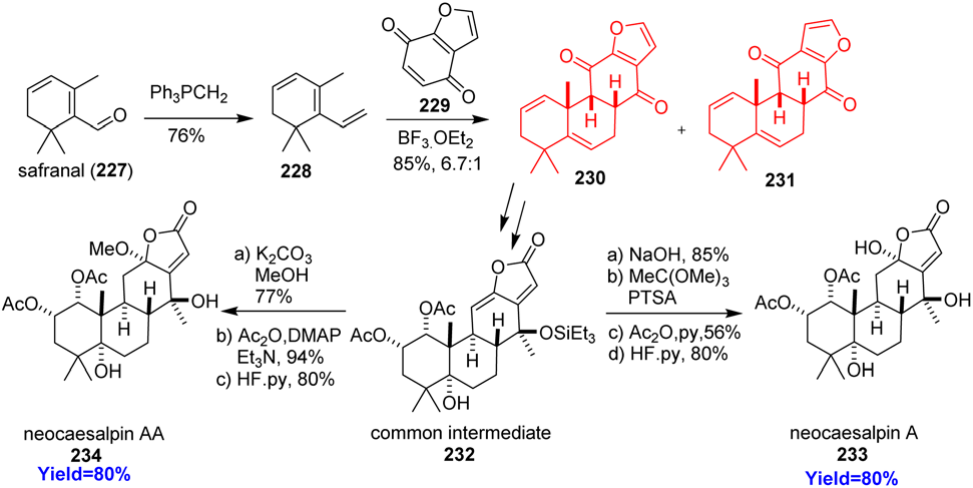
Total synthesis of neocaesalpin A (233), AA (234).^71^

Neocaesalpin A, AA, K all belongs to the cassane-type diterpenoids having anti-malarial, anti-viral, anti-inflammatory, anti-tumor, anti-oxidant properties.^69,70^ The first cassane-type diterpenoid neocaesalpin A isolated from *Caesalpinia bonduc*. Papidocha *et al.*^71^ commenced the total synthesis with safranal 227 which produced the Diels–Alder precursor triene 228 (Scheme 29). The triene 228 and furanoquinone 229 produced the desired cycloadduct 230 with its regioisomer 231 in 6.7 : 1 ratio. The cycloadduct 230 converted to the intermediate 232 which formed neocaesalpin A 233 in four steps and neocaesalpin AA 234 in 3 steps. At last, Papidocha and co-workers mentioned that neocaesalpin AA and neocaesalpin K might be the same natural product.^71^

## 3. Intramolecular Diels–Alder (IMDA) reaction

### 3.1. Synthesis of fused pyridine motif and total synthesis of lycopodium alkaloids

**Scheme 30:**
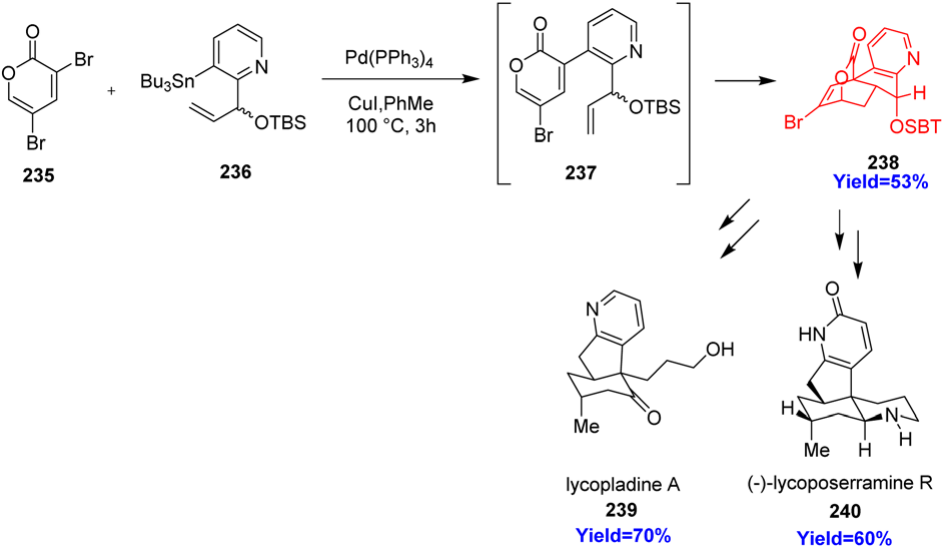
Stille coupling between (235) and (236) accompanied with IMDA reaction produced (238) which further can be coverted to the lycopladine A (239) and (−)-lycoposerramine R (240).^72^

Pyrindane moiety (pyridine unit fused to a cyclopentane ring) has gained recent attention for its occurrence in bioactive natural products and drugs like rasagiline, ipidacrine, carinatine A, (+)-lycopladine A, pyridinohopane, cimicifugadine and (−)-lycoposerramine. K. H. Sim *et al.*^72^ have reported the total synthesis of lycopladine A and lycoposerramine R by constructing the fused pyrindane moiety. Lycopladine A and lycoposerramine R, a member of *Lycopodium* alkaloid family, showed biological activities and they are anticipated to treat Alzheimer's disease to improve memory loss.^73^ Lycoposerramine R consists of four asymmetric centers, 5/6 *cis* fused ring and a pyridone ring whereas lycopladine A possesses a pyridyl-fused hydrindanone core. K. H. Sim *et al.*^72^ used 3,5-dibromo-2-pyrone 235 as substrate which undergoes Stille coupling with 236 to produce 2-pyrone having a chiral branched allylic silyl ether group 237 (Scheme 30). The product 237 underwent intramolecular Diels–Alder(IMDA) reaction with very high π-facial- and endoselectivities in a tandem fashion. The resulting cycloadduct 238 was transformed into the (+)-lycopladine A 239 and (−)-lycoposerramine R 240 in several steps.^72^

### 3.2. Total synthesis of the (±)-cephanolides A–D

**Scheme 31:**
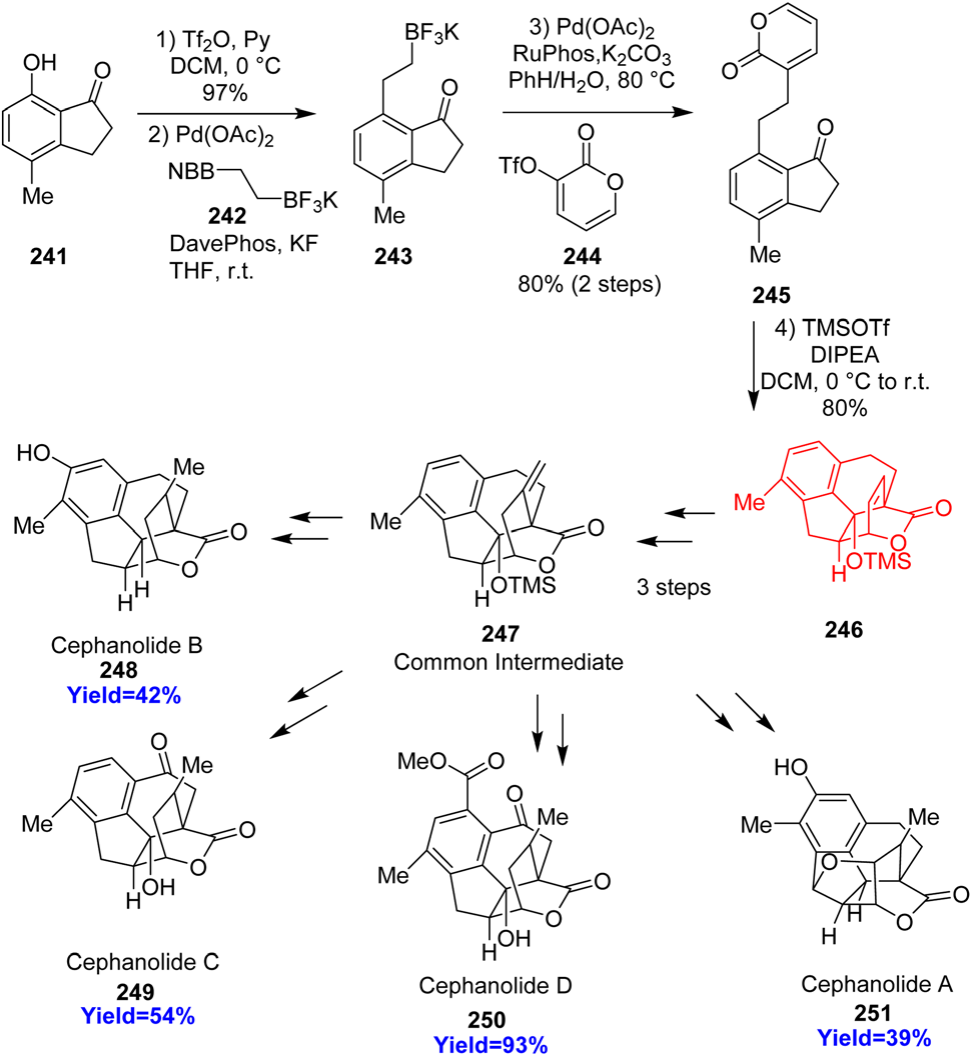
Total synthesis of cephanolide congeners (248–251).^77^

Cephanolides A–D belong to the cephalotaxus norditerpenoids family, which have shown a broad range of activity like anti-viral, anti-tumor, anti-neoplastic properties and can also control plant growth inhibition.^74–76^ These substances cytotoxic activity against human cancer cell lines relies upon the ring A. Haider *et al.*^77^ commenced the total synthesis with 7-hydroxy-4-methylindanone 241, which underwent Suzuki cross coupling reacting with BF_3_K-ethylene-9BBN 242 (Scheme 31). The formed product 243 reacted with pyrone triflate 244 and 245 underwent intramolecular Diels–Alder cycloaddition. The *endo* Diels–Alder adduct 246 was transformed to the common intermediate 247. Finding a common, versatile intermediate 247 that could be used for the preparation of all the cephanolide congeners like cephanolide A 251, cephanolide B 248, cephanolide C 249, and cephanolide D 250 was the goal.^77^

### 3.3. Total synthesis of (−)-scabrolide A

**Scheme 32:**
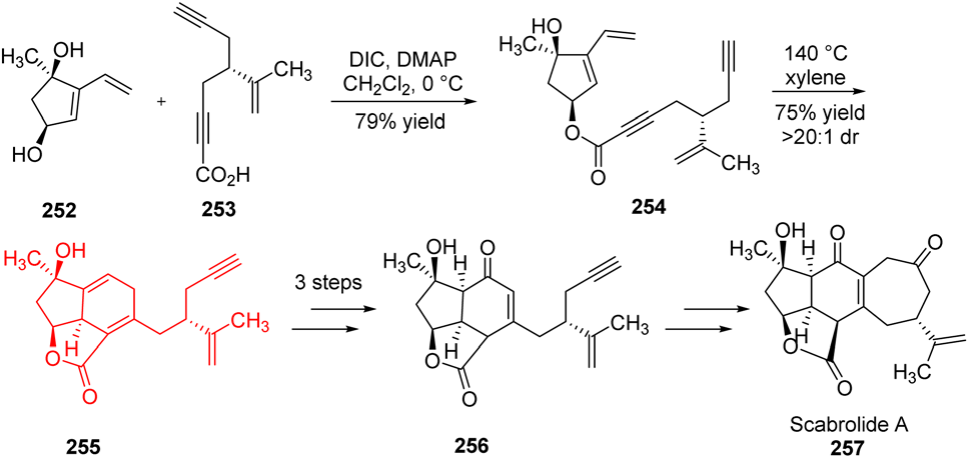
Total synthesis of scabrolide A (257).^78^

Scabrolide A belongs to the family of polycyclic furanobutenolide derived norcembranoid diterpenoids and shows anti-inflammatory properties. Hafeman and co-workers^78^ reported the first ever total synthesis of any member of polycyclic furanobutenolide derived norcembranoid diterpenoid family. They have developed a retrosynthetic route of scabrolide A, and commenced its total synthesis. Using it they have prepared two chiral compound dihydroxyvinylcyclopentene 252 and ynoic acid 253 as substrates which underwent esterification to introduce all the carbon atoms of the natural product (Scheme 32). Compound 254 formed 255 through intramolecular Diels–Alder reaction which in turn formed the key intermediate enone 256 in 3 steps. The enone underwent several transformations to form scabrolide A 257.^78^

### 3.4. Total synthesis of brevianamide A

**Scheme 33:**
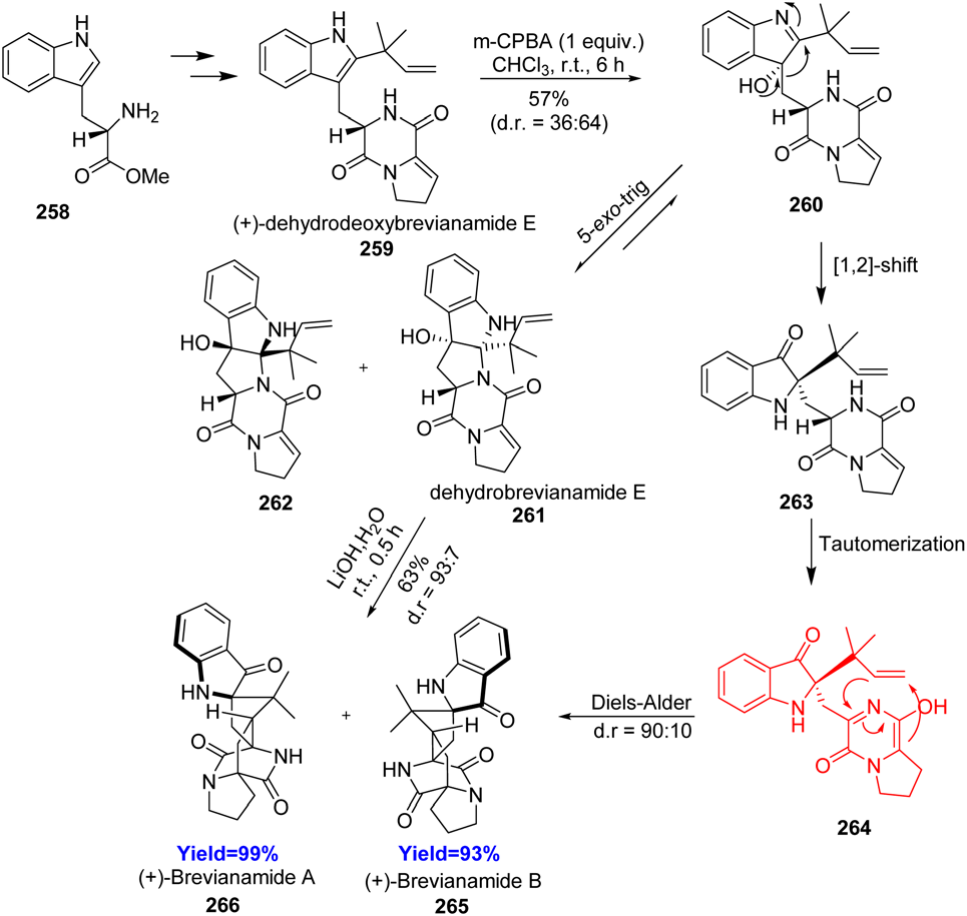
Total synthesis of brevianamide (265 and 266).^79^

Brevianamides A was isolated from the fungus *Penicillium brevicompactum*, and one of the first known bicycle[2.2.2]diazaoctane alkaloids. Insecticidal brevianamides fall in the group of dioxopiperazine type structures of bicyclo[2.2.2]diazaoctane alkaloids. Godfrey *et al.*^79^ reported the chemical synthesis of (+)-brevianamide A in seven steps from commercially available l-tryptophan methyl ester 258 (Scheme 33). This starting material formed the biosynthetic precursor (+)-dehydrodeoxybrevianamide E 259. At first, diastereoselective epoxidation of the indole moiety of 259 followed by intramolecular epoxide-opening, which produced mixture of diastereomeric dehydrobrevianamide E 261 and 262. The mixture gave (+)-brevianamide A 266 and (+)-brevianamide B 265 through a complex cyclization-cascade when exposed to LiOH in water for 30 minutes. The cascade involved retro-5-*exo*-trig/[1,2]-alkyl shift/Diels–Alder reaction where the retro-5-*exo*-trig cyclization opened up the pyrrolidine ring for [1,2]-alkyl shift. The alkyl shift placed the diene and the dienophile in close proximity though the dienophile was formed in the next step *via* tautomerization. At last, intramolecular diastereoselective [4+2] cycloaddition of 264 led to target molecule.^79^

### 3.5. Total synthesis of pepluanol A

**Scheme 34:**
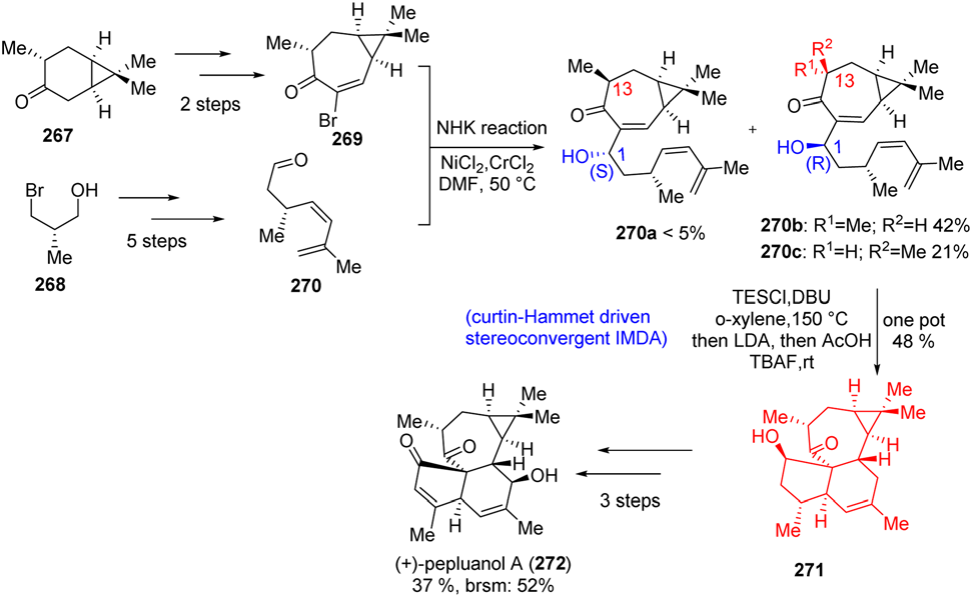
Total synthesis of pepluanol A (272).^43^

Pepluanol A, belongs to the family of *Euphorbia* diterpenes and isolated from *Euphorbia* plants, which behave as medicinal plants. These diterpenes had anti-viral, anti-tumor, cytotoxic and multidrug-resistance-reversing (MDR) properties.^80–82^ Yuan *et al.*^83^ had produced bromocycloheptenone 269 and an aldehyde 270 from commercially available compounds (Scheme 34). These two compounds formed the Diels–Alder precursor 270 through Nozaki–Hiyama–Kishi (NHK) reaction. The NHK reaction produced three different diasteroisomers (270a, b, c) differing in the absolute configuration of C_1_ and epimerization of methyl group at C_13_ position. After so many experiments, the experimental results suggest that Curtin–Hammett like situation arise in the Diels–Alder reaction. The C_13_ α-epimer 270b failed to undergo the DAR contrast to the C_13_ β-isomer 270c. From DFT calculation they have shown that the DA adduct formed from C_13_ β-isomer is less stable than the DA adduct formed from C_13_ α-isomer. After the DAR, the inversion of C_13_ stereocenter from β to α had been done using strong base LDA followed by quenching with acetic acid. Oxidation of the C_1_–OH of 271 followed by Saegusa–Ito oxidation and allylic oxidation led to the natural product (+)-pepluanol 272.^83^

### 3.6. Total synthesis of talatisamin

**Scheme 35:**
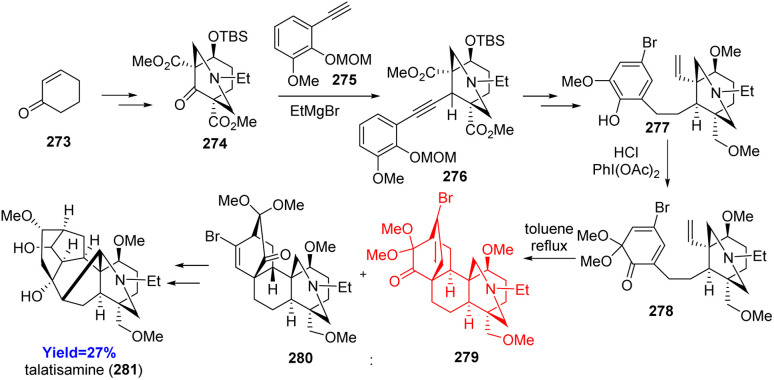
Total synthesis of talatisamin (281).^84^

Talatisamin, a member of C19 diterpenoid family, isolated from *Aconitum* species displays anti-arrhythmic activities and K^+^ channel inhibitory.^84^ Talatisamine composed of six fused rings (ABCDEF) with 12 contiguous stereocenters. Kamakura and coworkers^84^ have synthesized talatisamin in 33 steps from cyclohexenone 273. Cyclohexenone 273 was converted to azabicycle 274 (AE ring) and coupled with aromatic D ring 275 to form 276 (Scheme 35). It went oxidative dearomatization of D ring followed by intramolecular Diels–Alder reaction of 278 to form 279 which can be transformed to talatisamin 281 in 9 steps.^84^

### 3.7. Total synthesis of pepluanol

**Scheme 36:**
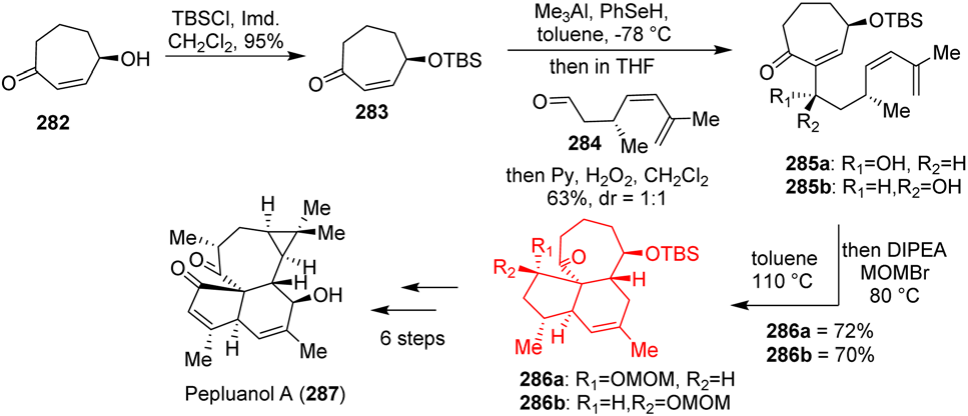
Total synthesis of pepluanol (287).^85^

Yuan *et al.*^85^ have developed *Euphorbia* diterpenoid pepluanol in only 11 steps from 1,3 cycloheptadiene. These natural products could be used as herbal folk medicine because of their anti-tumor, cytotoxic, anti-viral, anti-inflammatory properties.^80–82^ The total synthesis started with 282 to form 283 which on Morita–Baylis–Hillman reaction with an aldehyde 284 produced the Diels–Alder precursor 285 (Scheme 36). The intramolecular Diels–Alder reaction was conducted overnight at 110 °C in *o*-xylene successfully. The cycloadducts 286 were converted to pepluanol 287 in 6 steps.^85^

### 3.8. Total synthesis of havellockate

**Scheme 37:**
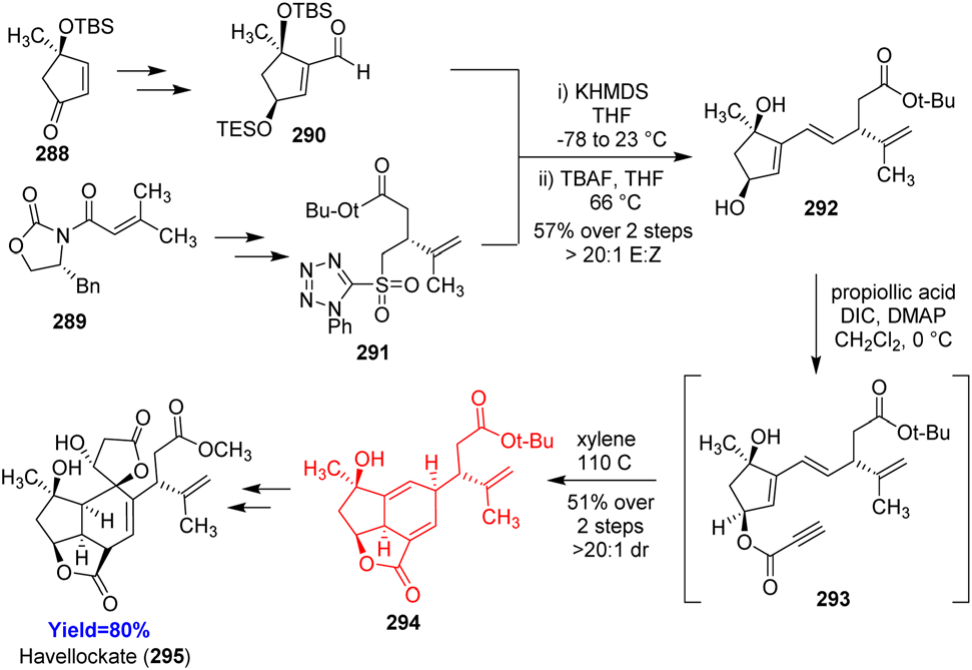
Total synthesis of havellockate (295).^86^

Havellockate, a furanobutenolide-derived cembranoid and isolated from *Sinularia granosa*, has a unique spiro-fused β-hydroxybutanolide ring. Hafeman and co-workers^86^ began to prepare aldehyde 290 and sulfone 291 from enone 288 and acyl oxazolidinone 289 respectively (Scheme 37). Julia-Kocienski olefination product 292 of aldehyde 290 and sulfone 291 produced the Diels–Alder precursor 293 on Steglich esterification. The [5-5-6] framework of havellockate was constructed through the intramolecular Diels–Alder reaction of 293 and converted to the targeted compound havellockate 295.^86^

### 3.9. Total synthesis of daphgraciline

**Scheme 38:**
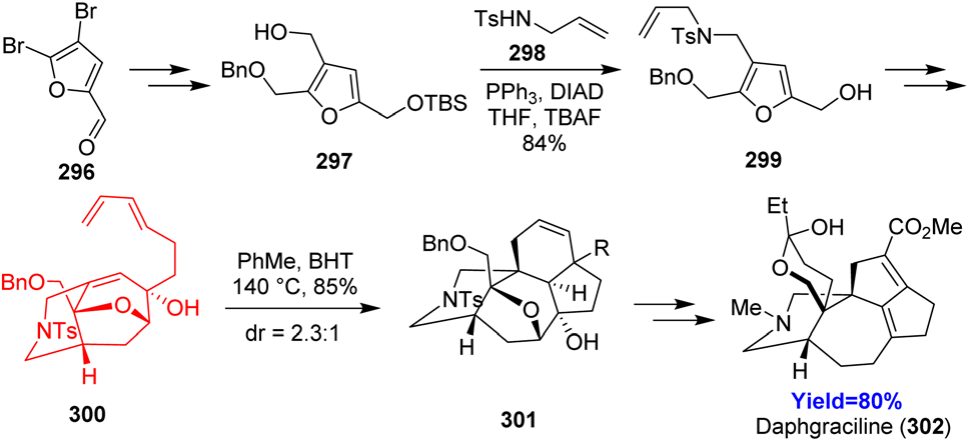
Total synthesis of daphgraciline (302).^87^

Li *et al.*^87^ reported the synthesis of daphgraciline, a *Daphniphyllum* yuzurine-type alkaloid comprising of [6-7-5-5-6] pentacyclic core, unique azabicyclo[4.3.1] system and a spiro tetrahydropyran ring. Based on the retrosynthesis, Mitsunobu reaction was performed between 297 (prepared from commercially available 296) and 298 (Scheme 38). The Mitsunobu product 299 was converted to the Diels–Alder substrate 300 through a [5+2] cycloaddition forming the azabicyclo[4.3.1] system. The desired mixture of diastereomers 301 was obtained in the intramolecular Diels–Alder reaction which was converted to daphgraciline (302).^87^

### 3.10. Total synthesis of erectones A, B and hyperelodione D

**Scheme 39:**
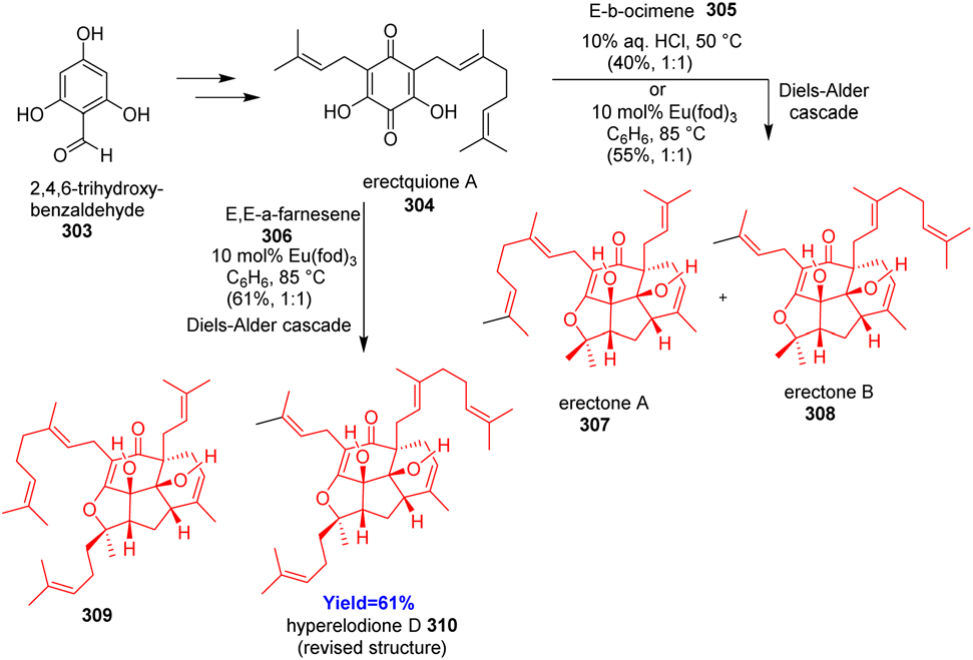
Total synthesis of erectones A (307), B (308) and hyperelodione D (310).^88^

Franov and co-workers^88^ reported bioinspired total synthesis of erectones A, B and hyperelodione D which belong to the polycyclic polyprenylated acylphloroglucinols family of natural products. Moreover, the structure of hyperelodione D was revised since the NMR spectra of the proposed structure and natural structure not matched. The synthesis commenced with the preparation of common intermediate erectquione A 304 from 2,4,6-trihydroxybenzaldehyde 303 (Scheme 39). Erectquione A 304 then formed erectones A 307, B 308 and hyperelodione D 310*via* Diels–Alder cacade with *E*-β-ocimene 305 and *E*,*E*-α-farnesene 306 respectively.^88^

### 3.11. Bioinspired total synthesis of ophiorrhine A

**Scheme 40:**
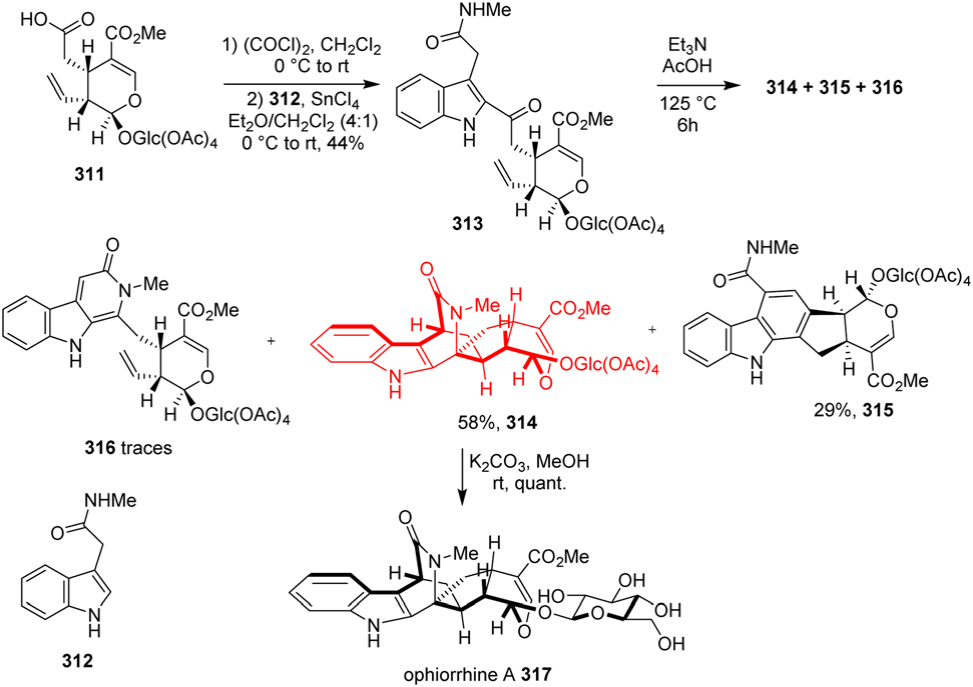
Bioinspired total synthesis of ophiorrhine A (317).^89^

Indole alkaloids ophiorrhine A, isolated from *Ophiorrhiza japonica*, exhibits immunosuppressive activity. Cao *et al.*^89^ synthesized this natural product from the acid of secologanin aglycon ethyl ether 311 which converted to its acid chloride form (Scheme 40). Friedel–Crafts type reaction with amide 312 formed 2-(2-acyl-3-indolyl)-acetamide 313 and it was aimed to convert to the key indolopyridone 316. Rather 316 was found in traces amount because transient 316 underwent intramolecular Diels–Alder cycloaddition to construct spirocyclic azabicyclo[2.2.2]octanone 314. On methanolysis of acetates of glucose moiety produced ophiorrhine A 317.^89^

### 3.12. Total synthesis of yonarolide

**Scheme 41:**
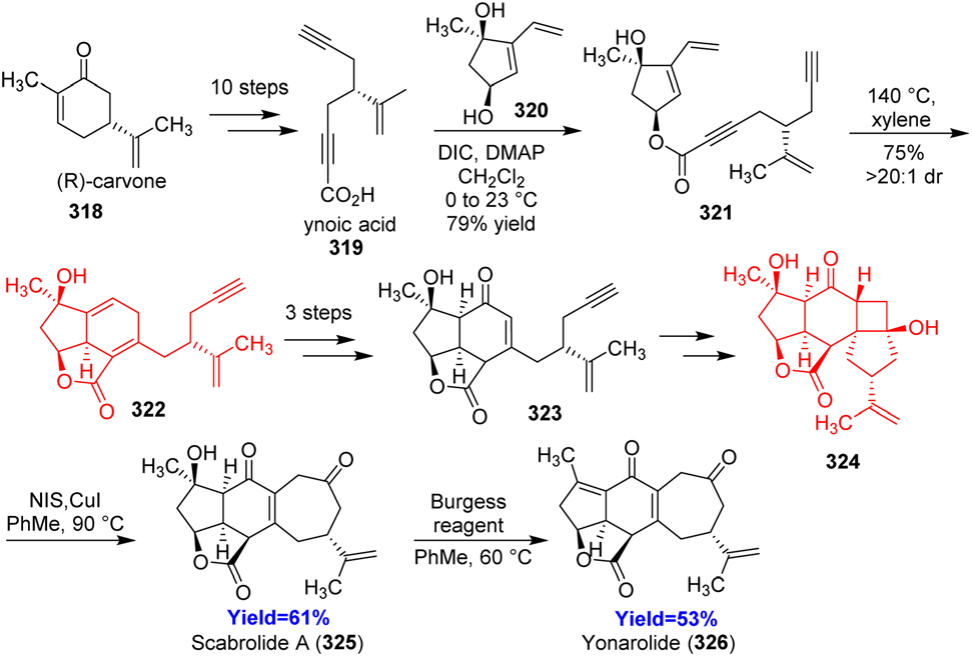
Total synthesis of scabrolide A (325) and yonarolide (326).^90^

Scabrolide A and yonarolide, belong to the polycyclic furanobutenolide derived norcembranoids, were isolated from soft corals *Sinularia*. Hafeman and co-workers^90^ have showed the challenges they faced during the synthesis and how they overcame those challenges. (*R*)-Carvone 318, the starting material of this synthesis, was converted to ynoic acid 319 which then underwent esterification with diol 320 to form Diels–Alder substrate 321 (Scheme 41). Intramolecular Diels–Alder adduct 322 on epoxidation/reductive epoxide opening formed a diol which was oxidized selectively to produce 323. The major steps involved during the last several transformations were [2+2] photocycloaddition, Tamao–Fleming oxidation and Grieco elimination to reach scabrolide A 325 and yonarolide 326.^90^

### 3.13. Total synthesis of vilmoraconitine

**Scheme 42:**
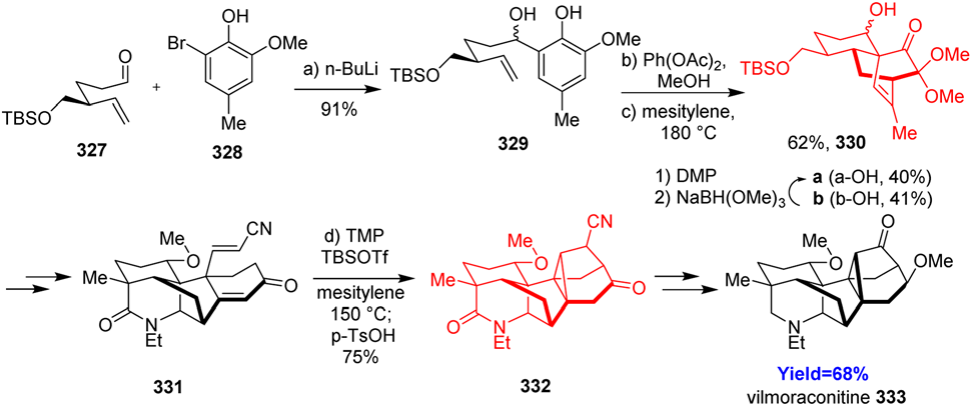
Total synthesis of vilmoraconitine (333).^94^

Vilmoraconitine, a C_19_-diterpenoid alkaloid, isolated from medicinal plant *Aconitum*. These natural products exhibit analgesic, anti-inflammatory and cardioactive effects.^91–93^ Jiujian Ji and co-workers^94^ commenced the total synthesis with aldehyde 327 and bromide 328 according to their retrosynthetic analysis (Scheme 42). The phenol adduct 329 underwent oxidative dearomatization followed by Diels–Alder when treated with Ph(OAc)_2_ in methanol. Though a pair of separable diastereoisomers 330a, b has been produced, the undesired one 330b was recycled to the desired one 330a effectively. Another Diels–Alder reaction was required in the last stage of this total synthesis to form 332 from 331.^94^

### 3.14. Total synthesis of lancilactone C

**Scheme 43:**
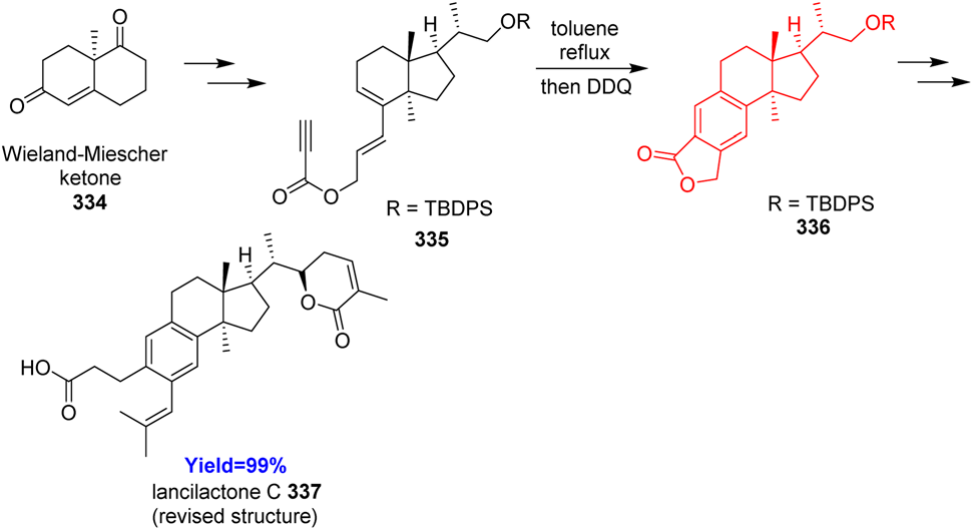
Total synthesis of lancilactone C (337).^96^

Lancilactone C, found in the stems and roots of *Kadsura lancilimba*, a Chinese folk medicine used for the anti-HIV activity.^95^ Kuroiwa *et al.*^96^ developed the total synthesis of lancilactone C for the very first time and commenced with Wieland–Miescher ketone 334. After forming the proposed structure of lancilactone C and comparing with the NMR data, the proposed structure was revised. The total synthesis of the revised structure also started with Wieland–Miescher ketone 334 and converted to the Diels–Alder precursor 335 which underwent intramolecular Diels–Alder reaction (Scheme 43). The Diels–Alder adduct 1,4-diene on oxidation with DDQ produced benzene ring in 336.^96^

### 3.15. Total synthesis of zygadenine

**Scheme 44:**
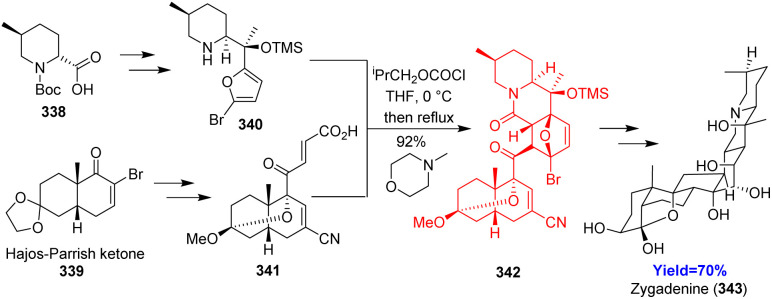
Total synthesis of zygadenine (343).^97^

Zygadenine natural products belong to the cevanine subgroup of *Veratrum* genus of plants having medicinal properties. They can be used for the treatment of injuries, pain, hypertension *etc.* Guo and co-workers^97^ began their synthesis according to their retrosynthetic analysis. Substrates acid 338 and Hajos-Parrish ketone 339 formed the diene 340 and the dienophile 341 part respectively which upon coupling produced the Diels–Alder precursor (Scheme 44). Since the precursor had both the diene and dienophile counterpart it underwent intramolecular Diels–Alder reaction. The adduct 342 was converted to the target molecule zygadenine 343 in more than 20 steps.^97^

### 3.16. Total syntheses of cinchona alkaloids

**Scheme 45:**
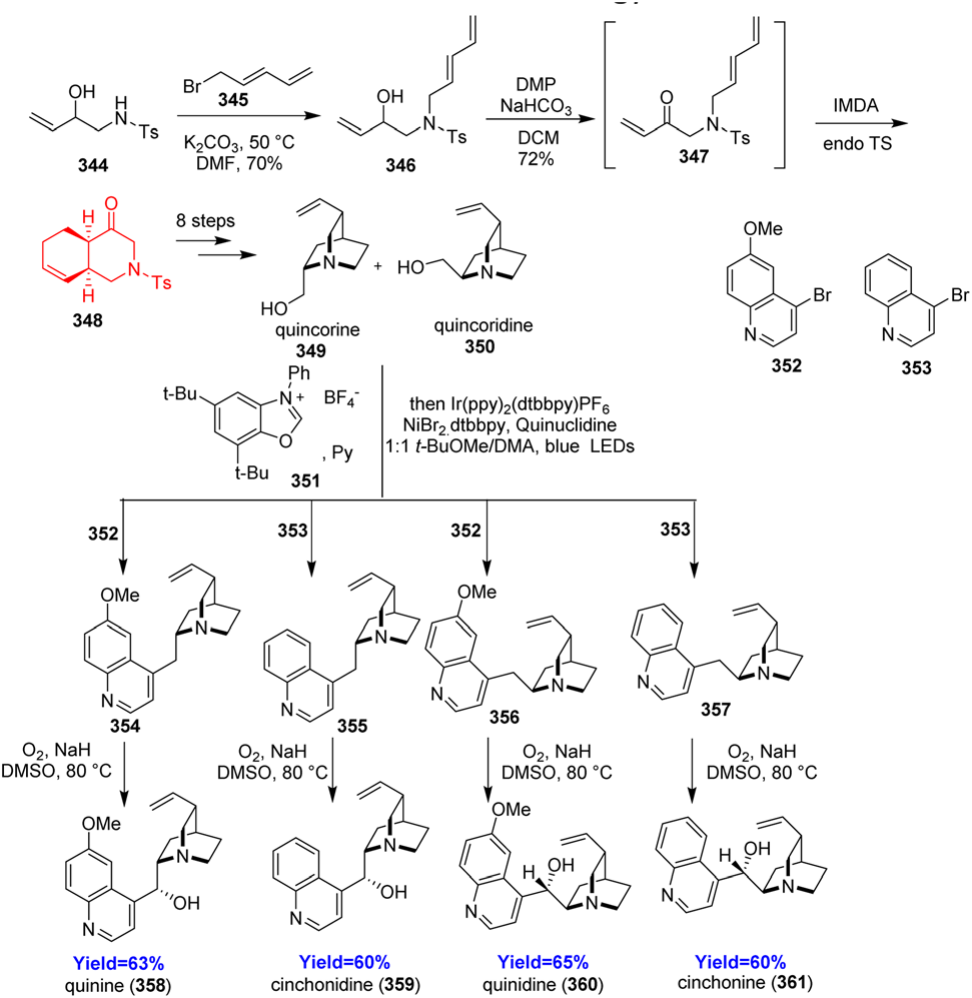
Total syntheses of cinchona alkaloids (358–361).^99^

Quinine, quinidine, cinchonine, cinchonidine being the members of cinchona alkaloids are famous for their anti-malarial activities.^98^ Though there are several synthetic routes to cinchona alakaloids, preparing quincorine and quincoridine (precursors of cinchona alkaloids) remains difficult and lengthy. Lei Li and co-workers^99^ hence *de novo* synthesized these two alcohols through a key intramolecular Diels–Alder reaction. Two known compounds 344 and 345 initiated the synthesis and the formed product 346 was oxidized before intramolecular cycloaddition (Scheme 45). Then the cycloadduct 348 was converted to quincorine 349 and quincoridine 350 in 8 steps. Next, the cinchona alkaloids 358–361 have been produced using metallaphotoredox-catalyzed deoxygenative arylation based on Macmillan's recent methodology.^99^

### 3.17. Total syntheses of tetramic acid bearing *cis*-decalin natural products

**Scheme 46:**
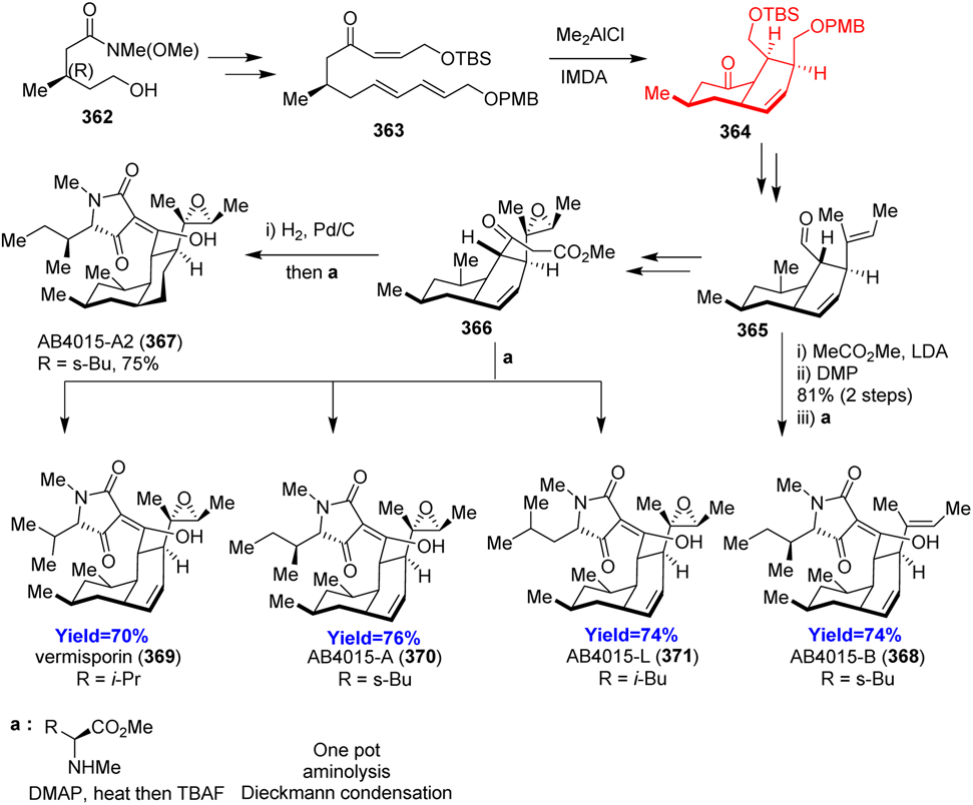
Total syntheses of tetramic acid bearing *cis*-decalin natural products.^100^

Dong *et al.*^100^ developed an alternative strategy for the synthesis of AB4015-A2 and four antibiotics vermisporin, PF1052/AB4015-A, AB4015-B, AB4015-L. These natural products work excellently against Gram-positive bacteria and anaerobic bacteria and being isolated from fungi. Dong and co-workers followed their retrosynthetic analysis and commenced with Weinreb amide 362 as starting material (Scheme 46). The amide on Mitsunobu reaction, oxidation followed by Julia olefination produced triene intramolecular Diels–Alder precursor 363. All these anti-biotics 367–371 were formed through a one pot aminolysis/Dieckmann condensation cascade in the presence of l-amino acid derivatives.^100^

### 3.18. Synthesis of 6/6/5-fused tricyclic terpenoid scaffold

**Scheme 47:**
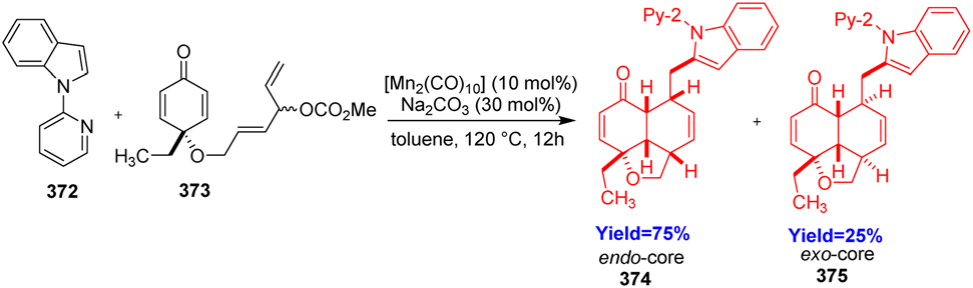
Synthesis of 6/6/5-fused tricyclic terpenoid scaffold.^101^

6/6/5-Fused tricyclic scaffold is a central core of many complex natural products like nagilactones, azadirachtin, endiandric acid A and marrubin. Parammal and co-workers^101^ opened a new way to construct this type of scaffold through manganese catalyzed C–H activation. 2-Pyridinyl indole 372 and cyclohexadienone 373 were taken as substrate in the presence of [Mn_2_(CO)_10_] catalyst and Na_2_Co_3_ additive in toluene (Scheme 47). C–H dienylation of 2-pyridinyl indole produced an intermediate which underwent intramolecular Diels–Alder reaction to give the 6/6/5-fused tricyclic scaffold 374, 375.^101^

### 3.19. Total syntheses of cassane furanoditerpenoids

**Scheme 48:**
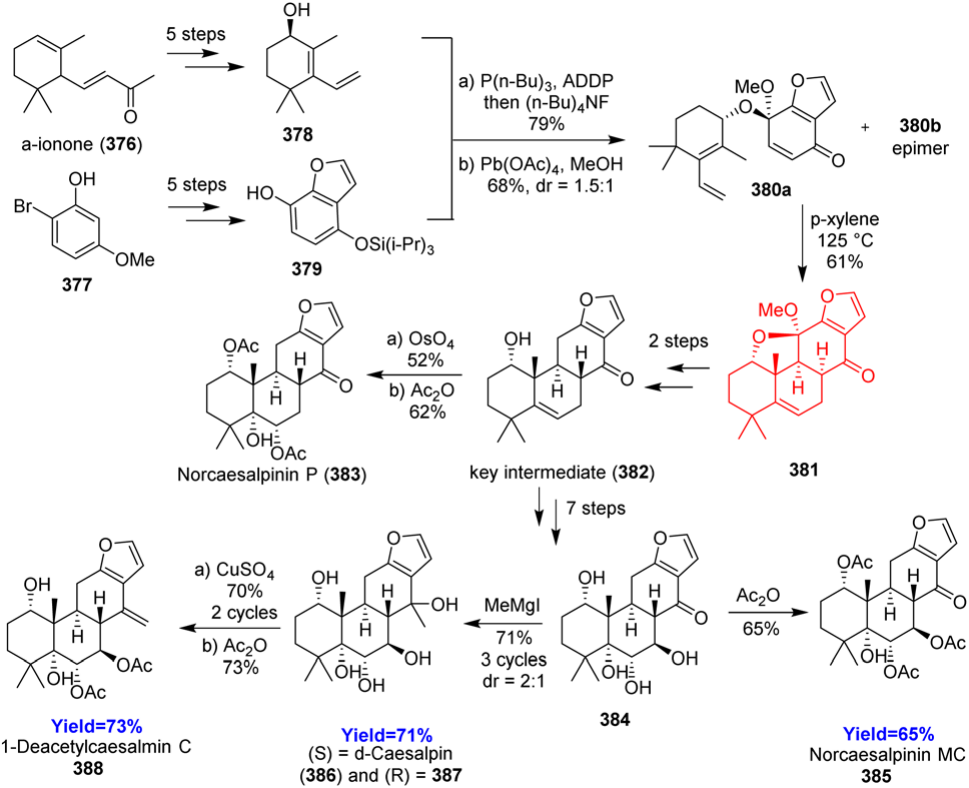
Total syntheses of cassane furanoditerpenoids.^102^

Bulthaupt and co-workers^102^ reported first ever total syntheses of norcaesalpinin MC, δ-caesalpin, 1-deacetylcaesalmin C, and norcaesalpinin P. Based on their retrosynthetic path, synthesis commenced with the preparation of the (*R*)-diene 378 from α-ionone 376 and phenol 379 from 377 (Scheme 48). After preparing, both 378, 379 were coupled using Tsunoda's modification of Mitsunobu reaction followed by desilylation and oxidative dearomatization to produce the Diels–Alder precursor 380a, b as diastereomeric mixture. The mixture was heated in *p*-xylene to form single diastereomeric Diels–Alder adduct 381 which then led to the key intermediate 382 and further modifications produced the above-mentioned natural products.^102^

### 3.20. Total synthesis of abyssomicin 2 and neoabyssomicin B

**Scheme 49:**
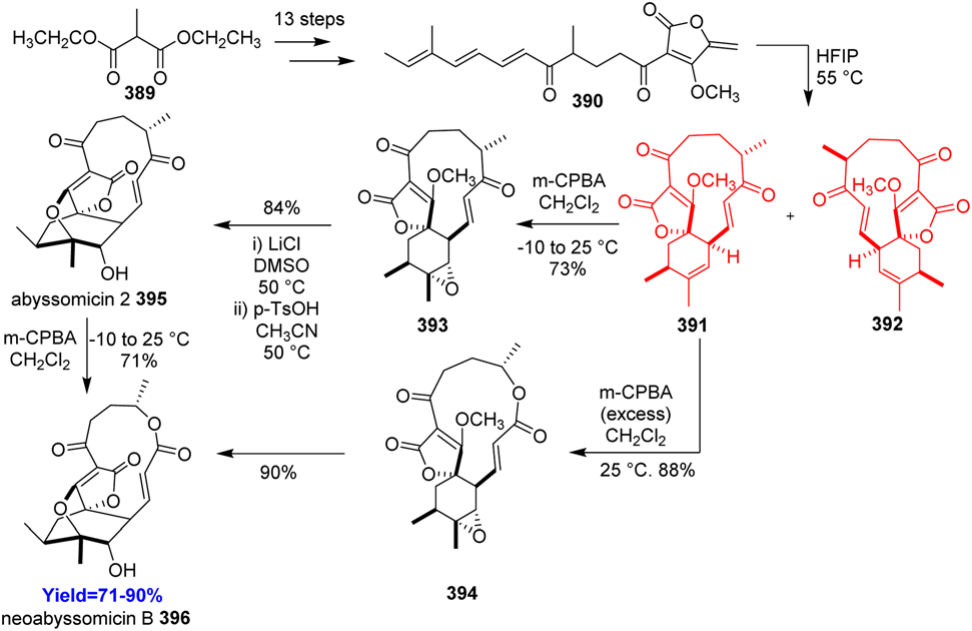
Total synthesis of abyssomicin 2 (395) and neoabyssomicin B (396).^58^

Abyssomicin 2, belongs to the subclass of class I spirotetronate polyketides, showed its anti-microbial activity against Gram-positive bacteria.^103^ Since, a bio-inspired approach *via* an intramolecular Diels–Alder reaction was successful for the construction of key intermediate of abyssomicin C, Canko *et al.*^104^ used that approach too here. Synthesis started with commercially available diethyl 2-methylmalonate 389 and aimed to form the Diels–Alder precursor diketone 390 (Scheme 49). It took 13 steps to form the diketone 390 which then underwent intramolecular Diels–Alder reaction in the HFIP solvent. Both abyssomicin 2 395 and neoabyssomicin B 396 was formed from one of the Diels–Alder adduct isomer in 2 to 3 steps.^104^

### 3.21. Total synthesis of atropurpuran

**Scheme 50:**
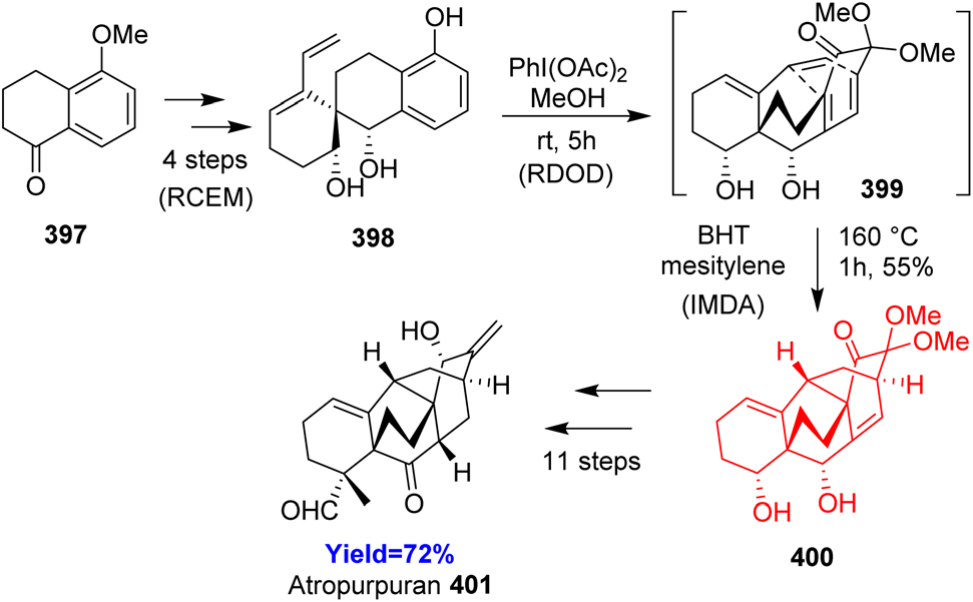
Total synthesis of atropurpuran (401).^105^

Atropurpuran, belongs to the arcutane-type diterpenoid, isolated from roots of *Aconitum hemsleyanum*. One of its structural feature is that it contains two contiguous bicyclo[2.2.2]octane motifs, which is usually synthesized by oxidative dearomatization-intramolecular Diels–Alder reaction. Jing Xu and co-workers^105^ decided to begin the synthesis from readily available 5-methoxytetralone 397 which produced 398 through ring closing enyne metatheis (RCEM) (Scheme 50). 398 underwent double-oxidative dearomatization (RDOD) to form dienone 399 the Diels–Alder precursor. The diol 400 formed from intramolecular Diels–Alder reaction of dienone 399 converted to the target molecule atropurpuran 401 in few steps.^105^

## 4. Dehydro Diels–Alder (DDA) reaction

### 4.1. Total synthesis of aspidosperma alkaloids using didehydro Diels–Alder reaction

**Scheme 51:**
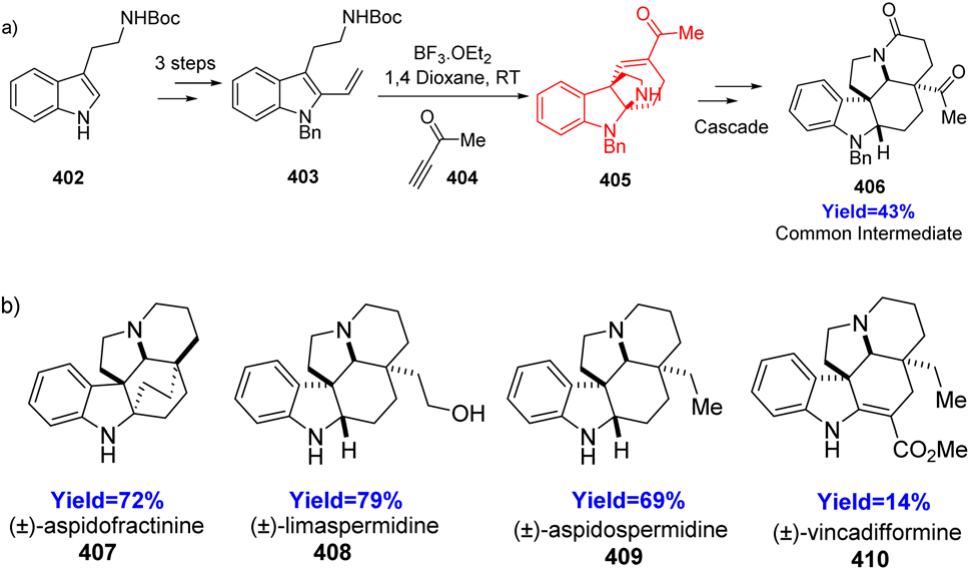
(a) Construction of the common intermediate 406.^106^ (b) Aspidosperma alkaloids formed from 406.^106^

A class of monoterpenoid indole alkaloids are called aspidosperma alkaloids. Techniques based on a common intermediate are likely the most effective strategies used in total synthesis. For the synthesis of aspidosperma natural products, Cain *et al.*^106^ reported a common intermediate method through a didehydro Diels–Alder reaction between 403 and 404 in which *N*-Boc tryptamine 402 is the starting material for cascade reactions leading to the common intermediate 406 (Scheme 51a).^106^

Additionally, they showed how useful this cascade technique was in the synthesis of that common intermediate 406 into (±)-aspidospermidine 409, (±)-aspidofractinine 407, (±)-limaspermidine 408, and (±)-vincadifformine 410 (Scheme 51b).

### 4.2. Intramolecular DDA reaction in aryldihydronaphthalene synthesis

**Scheme 52:**
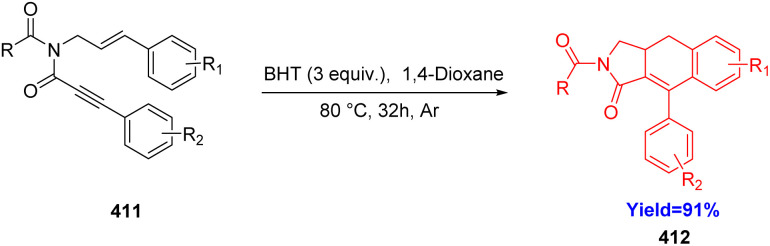
Synthesis of aryldihydronaphthalene.^107^

Although numerous methods are available for synthesizing aryldehydronaphthalene skeletons, [4+2] annulation reactions and didehydro Diels–Alder (DDDA) reactions of styrene–ynes are commonly acknowledged as particularly promising and effective strategies for their formation. These skeletons are essential components in various natural products and functional materials and can also serve as valuable precursors for synthesizing aryltetrahydronephthalenes and arylnaphthalenes. Chen *et al.*^107^ in 2022, solved various previous drawbacks and performed intramolecular DDDA reaction of styrene-ynes 411 under metal and catalyst free conditions with high chemoselectivity to produce aryldihydronaphthalene 412 (Scheme 52).^107^

Chen and co-workers^107^ have used 2,6-di-*tert*-butyl-4-methylphenol (BHT) as the antioxidant in 1,4-dioxane solvent. Further, with the increase of the reaction time to 32 h, the product yield also increased to 91% yield. In absence of BHT, but in presence of air or argon atmosphere the reaction yield was 88% and 89% yield respectively. Hence it is clear that addition of BHT is essential for the increase of yield of the product.

### 4.3. Photo-dehydro-DA reaction in total synthesis of arylnaphthalene lignans (ANLs)

Lignans, a large class of natural products distributed in different type of plants, belong to the large family of phenylpropanoids and consist of two C6 C3 units. Consequently, most lignans exhibit a C18 skeletons. Arylnaphthalene lignans (ANL) are wide spread secondary metabolites and exhibit a variety of biological properties like cytotoxicity, anti-microbial and anti-viral activity.^108^

Though various methods of total syntheses of ANLs are available, the PDDA reactions are the most convenient and straightforward way to synthesize arylnaphthalenes. Wessig and his co-workers^109^ in 2022, reported the PDDA reaction (*i.e.* the light-induced [4+2] cycloaddition between two arylacetylenes), as a key step in total syntheses of ANLs. They have approached through the intramolecular systems of 3-(hydroxyaryl)propiolic esters where the aryl propiolates were tethered by a linker unit to synthesize ANLs *via* a PDDA reaction. Suberic acid, chosen as a tethering unit because it can be easily introduced and removed, has suitable length to facilitate the reaction and has no desirable influence on photo radiation.

**Scheme 53:**
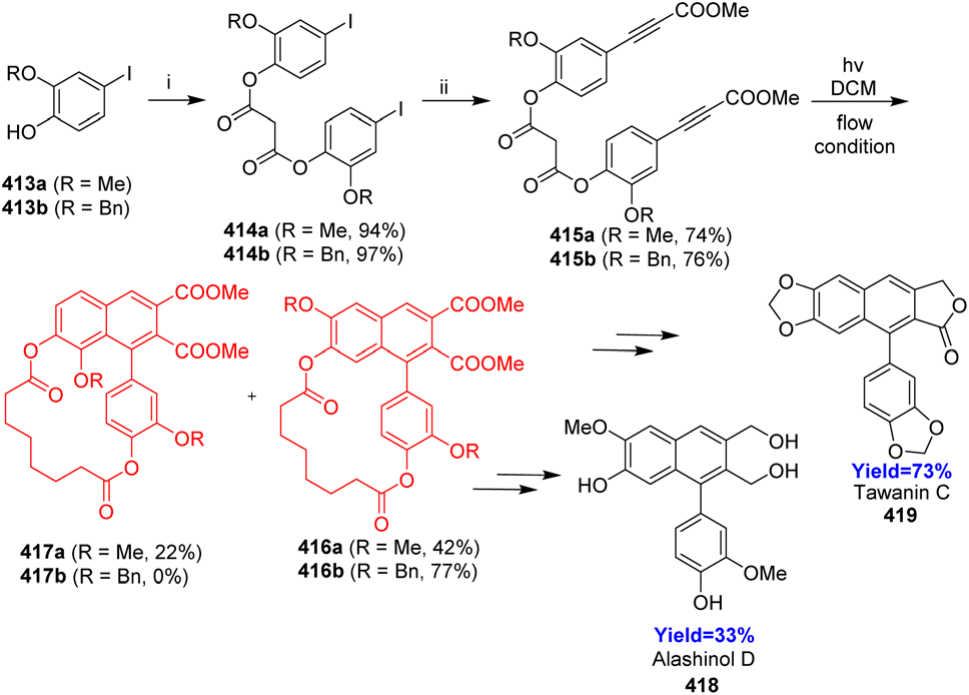
Total synthesis of alashinol D 418, taiwanin C 419 (i) suberic acid, DIC, DMAP, DCM; (ii) methyl propiolate, Pd(PPh_3_)_2_Cl_2_, CuI, K_2_CO_3_, THF.^109^

In the total synthesis of lignans like alashinol D 418, taiwanin C 419, they have taken 4-iodocatechols 413a, b having protected at 2 position formed iodophenyl suberates 414a, b through steglich esterification with suberic acid (Scheme 53). On Sonogashira coupling, methylpropiolates 415a, b were formed, which underwent PDDA reaction under flow condition resulting the formation of 416a and 417a in 2 : 1 ratio but when Bn group was used 416b formed exclusively. Further, 416a, b were used for the synthesis of ANLs like alashinol D 418, taiwanin C 419.^109^

## 5. Hetero-Diels–Alder (HDA) reaction

### 5.1. Aza Diels–Alder reaction in total synthesis of matrine alkaloids

**Scheme 54:**
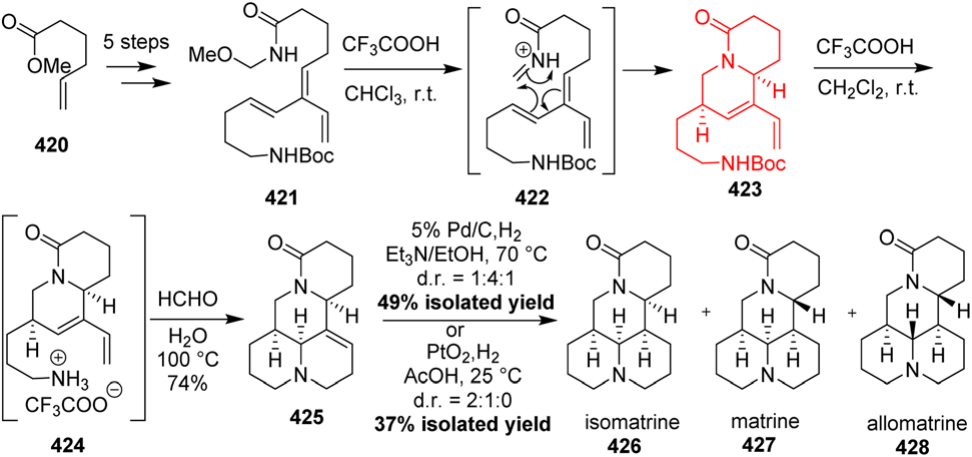
Aza Diels–Alder reaction in total synthesis of matrine alkaloids (426–428).^113^

Plants of the Sophora family are used in traditional medicine in Asia, South America and Australasia. Most of the known biologically active ingredients belong to the matrine alkaloid family. Matrines have anti-cancer effects by inhibiting the proliferation and inducing apoptosis of various cancer cell lines, have been used for the clinical treatment of hepatitis B and has antiviral activity.^110–112^ Starting material methyl hex-5-enoate 420 was converted to the Diels–Alder substrate 421 in 5 steps (Scheme 54). The first hetero intramolecular Diels–Alder reaction was observed in 422 to produce 423 which paved the way for the second one through 424. The tetracyclic core in 425 was thus generated using two intramolecular HDA reaction reported by Magann *et al.*^113^ On hydrogenation mixture of three matrine natural products 426–428.^113^

### 5.2. Oxa–Diels–Alder reaction in tetracyclic isochroman synthesis

Tetracyclic isochromans, one type of polyketide oligomers, are distributed in wide range of bioactive molecules and natural products. But, a general and stereoselective approach for the formation of the structure remains challenging due to their inherent instability and complex stereochemistry of polyketide.

**Scheme 55:**
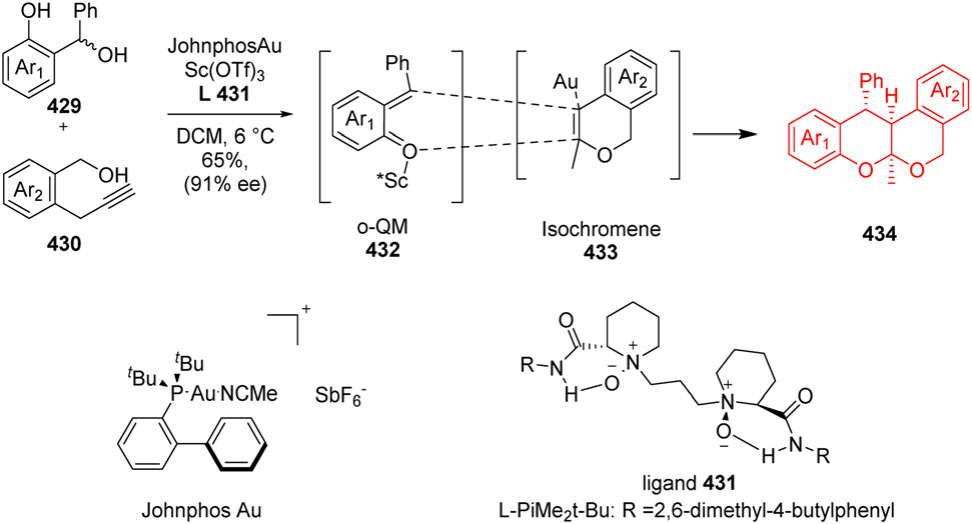
Construction of the isochroman motif 434.^114^

Herein (Scheme 55), Lin *et al.*^114^ synthesized polyketide oligomer using a combination of Au(i) catalyst and a chiral Sc(iii) Lewis acid catalyst. The isochromene 433 and *ortho*-quinonemethide 432 are *in situ* generated from readily available α-propargyl benzyl alcohols (430) and 2-(hydroxylmethyl) phenols (429) under mild conditions (Scheme 55). Isochromene 433 and *ortho*-quinonemethide 432 underwent oxo Diels–Alder reaction forming the tetracyclic isochroman 434.^114^

### 5.3. Divergent synthesis of chromenones by domino Knoevenagel-hetero-“Diels–Alder” (DKHDA) reaction

**Scheme 56:**
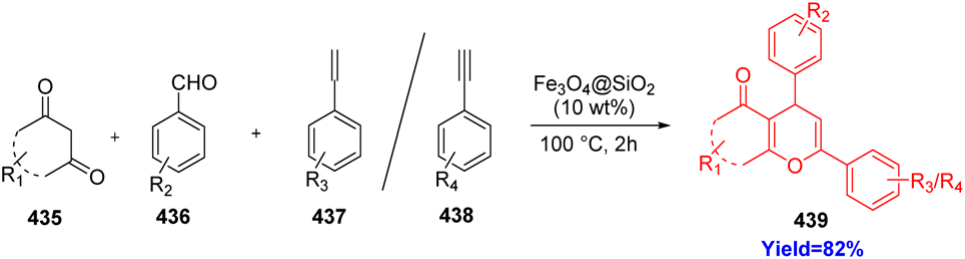
Synthesis of chromenons by solvent free, silica catalysed three component DKHDA reaction.^65^

Many natural products like flavonoids, alkaloids, anthrocyanins contain chromene core. The chromene derivatives exhibit anti-oxidant, anti-microbial, anti-cancer, molluscicidal properties.^115–118^ The DKHDA reaction has huge applications in the formation of synthetic drugs, natural products. Suri *et al.*^119^ synthesized chromenone derivatives 439 through a three-component reaction involving 1,3 dicarbonyl 435, aldehydes/ketones 436, and alkenes 437/alkynes 438 (Scheme 56).^119^ With no appreciable decrease in the catalytic activity, the catalyst could be employed for up to five consecutive catalytic cycles. Since, the reaction was performed under solvent free condition with a non-toxic, low cost, widely available silica catalyst, which was magnetically recoverable, reusable; this is considered as greener approach.^119^

### 5.4. Ball-milling synthesis of tetrahydroquinolines

**Scheme 57:**
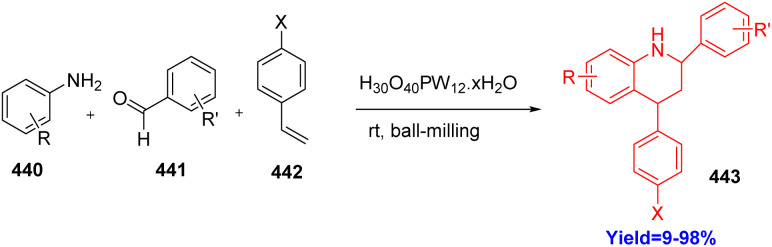
Ball-milling synthesis of tetrahydroquinolines.^120^

Tetrahydroquinolines are important scaffolds in various natural products. They show several pharmacological activities like excellent anti-thrombotic efficiencies in rabbit thrombosis. Though there are many ways to construct tetrahydroquinolines, Diels–Alder reaction remains the most efficient and successful route. Wang *et al.*^120^ have choosed phosphotungstic acid as a catalyst in this reaction because it can be used as a phase transfer catalyst as well as green catalyst. Moreover, they have taken various anilines 440, alkenes 442, benzadehydes 441 and performed solvent free (ball milling method) one pot three component Diels–Alder reaction at room temperature successfully to produce tetrahydroquinolines 443 with wide range of substrate scopes (Scheme 57).^120^

### 5.5. Total synthesis of stereochemically diverse isomers of spirooliganins

**Scheme 58:**
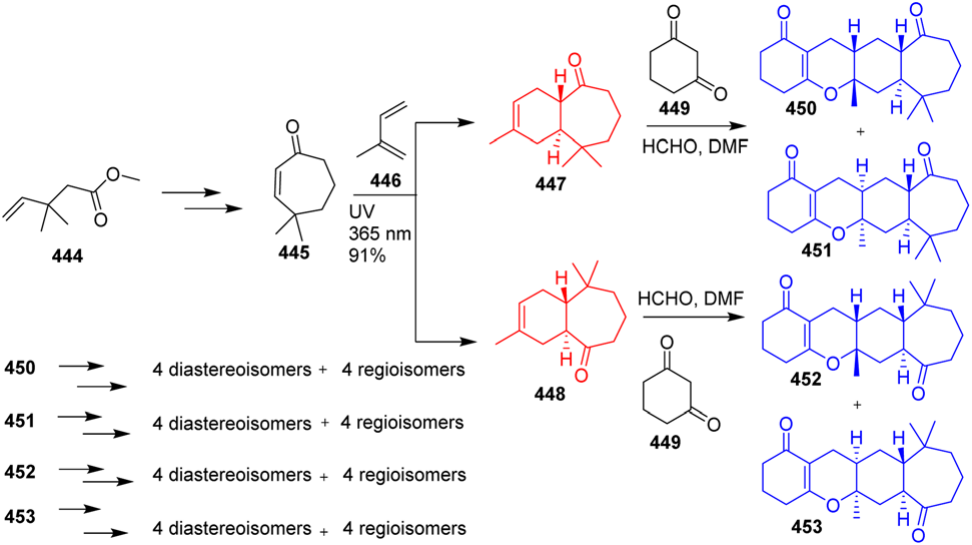
Total synthesis of stereochemically diverse isomers of spirooliganins.^121^

Spirooliganins, isolated from *Illicium oligandrum*, exhibit potent activity against coxsackievirus B3 (CVB3). Wang *et al.*^121^ have developed the total synthesis of a library of stereoisomers of spirooliganins *via* 17 steps reaction procedure. Commercially available methyl 3,3-dimethylpent-4-enoate 444 was the precursor of this total synthesis which was converted to 445 (Scheme 58). 445 and isoprene unit 446 was irradiated with 365 nm UV light which underwent Diels–Alder reaction to give a pairs of trans-fused regioisomers 447, 448. Both the regioisomers 447, 448 again underwent hetero-Diels–Alder cycloaddition with formaldehyde and 1,3-cyclohexanedione 449 to construct the cyclic core. The spirooliganins 450–453 have been produced from it through several conversions and finally Wang *et al.* constructed 16 diastereomers, 16 regioisomers of spirooliganin.^121^

### 5.6. Bioinspired total synthesis of pyritide A2

**Scheme 59:**
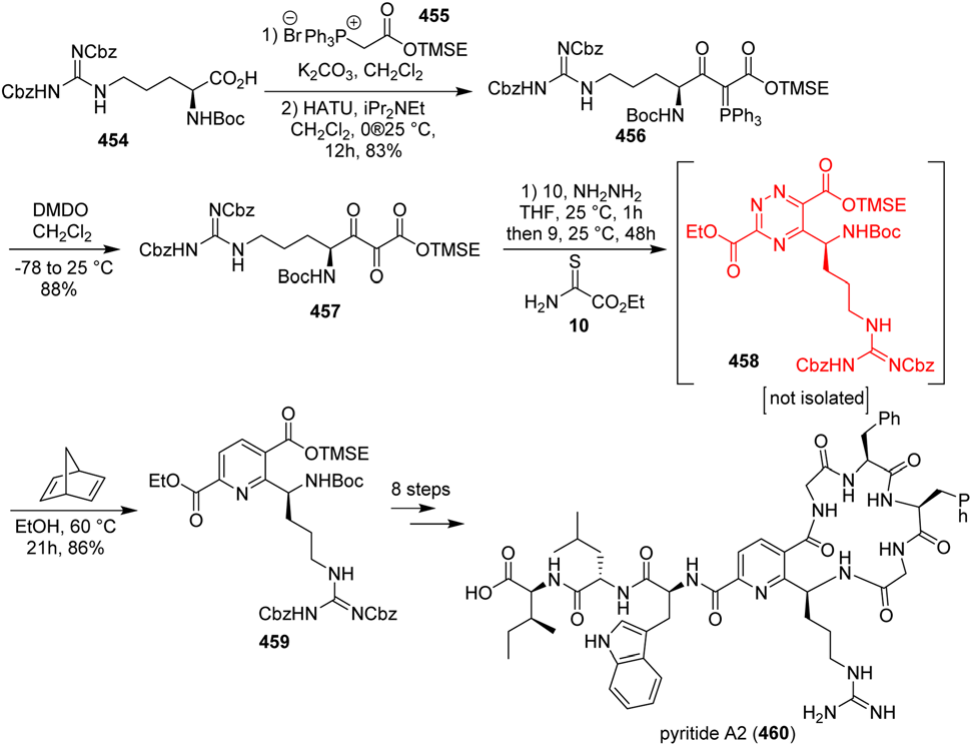
Bioinspired total synthesis of pyritide A2 (460).^122^

Natural product pyritide A2 belongs to the family of ribosomally synthesized and post-translationally modified peptides (RiPPs). This pyridine containing macrocyclic peptide pyritide A2 was identified from *Micromonospora rosaria* genome. Hooper *et al.*^122^ commenced the synthesis with the coupling of arginine derivative 454 and a phosphonium salt 455 followed by oxidative cleavage of C=P bond to form the tricarbonyl 457 (Scheme 59). The tricarbonyl was converted to the triazine 458 which on aza-Diels–Alder reaction by a solvent swap and addition of norbornadiene produced the pyridine core in 459 and at last pyritide A2 460.^122^

### 5.7. Bio-inspired formal total synthesis of bisabosqual A

**Scheme 60:**
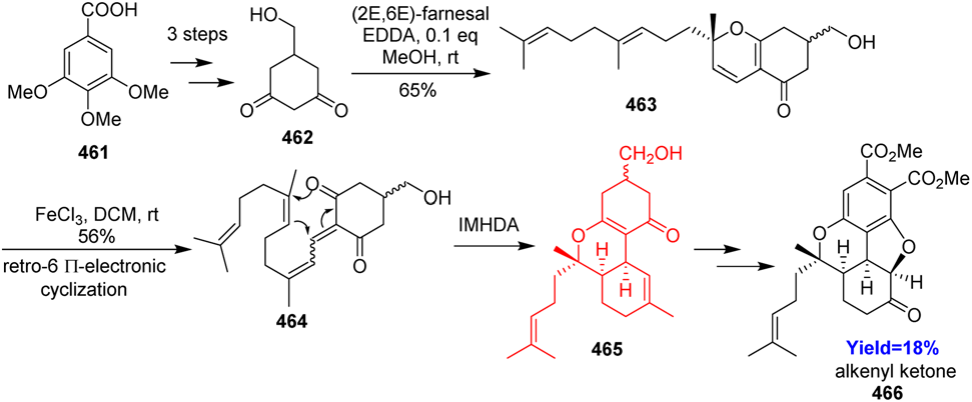
Bio-inspired formal total synthesis of bisabosqual A.^123,124^

Bisabosqual A was isolated from the culture broth of *Stachybotrys* sp. RF-7260 obtained from decaying tree leaves. A unique 6/6/5/6 ring system is the feature of bisabosqual A. Though there are several examples present to synthesize bisabosquals, there isn't any example of bio-inspired synthesis of bisabosquals. Xuanxuan Du and co-workers^123^ took this challenge and analysed bio-inspired retrosynthesis. Oxa [3+3] annulation started the synthesis which followed by retro-6π-electrocyclization and intramolecular hetero-Diels–Alder reaction in presence of Lewis acid (Scheme 60). The adduct 465 was converted to an alkenyl ketone 466 which could form bisabosqual A following the procedure of Parker's group.^123,124^

### 5.8. Total synthesis of penostatin A and C

**Scheme 61:**
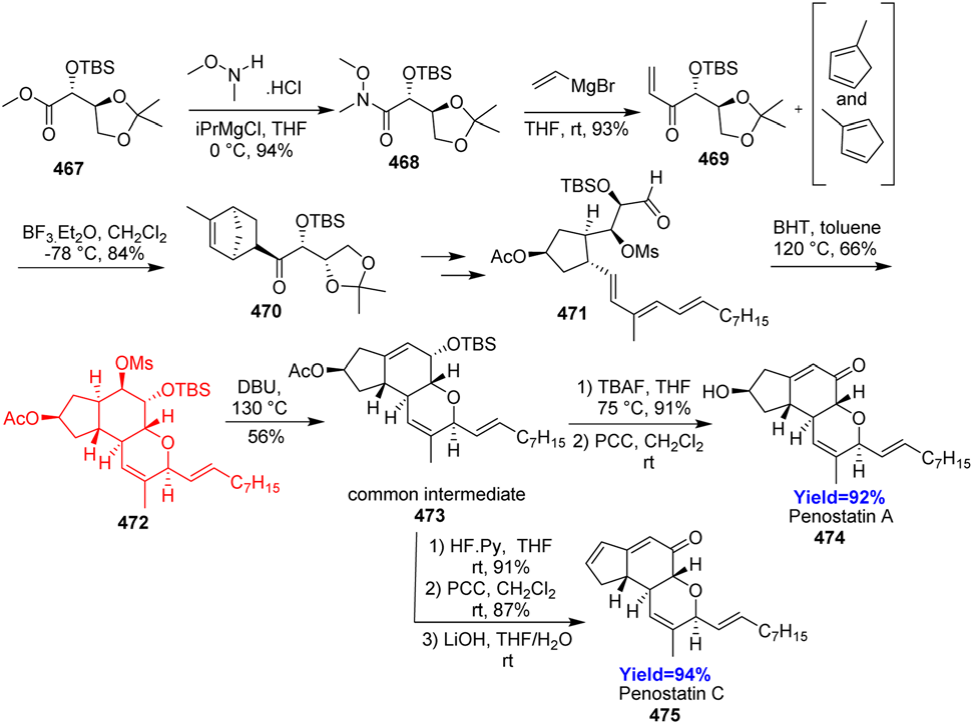
Total synthesis of penostatin A (474) and C (475).^125^

The penostatin family of natural products, isolated from *Penicillium* sp. OUPS-79 and *Penicillium* sp. CPCC 401423, exhibited cytotoxicity against cultured P388 cells. Penostatins A–C inhibited enzyme protein tyrosine phosphatase 1B (PTP1B), moreover penostatin C showed most inhibitory action and cytotoxicity against several other tumor cells. Jia *et al.*^125^ have taken an ester 467 prepared from l-ascorbic acid as substrate which transformed to a dienophile 469 in two steps (Scheme 61). The Diels–Alder adduct 470 formed with high chemo, regio and stereoselectivity was converted to another Diels–Alder precursor 471. A key intramolecular hetero-Diels–Alder reaction of the precursor 471 constructed the B and C ring of 472 which on elimination gave the common intermediate 473. One of its major advantage is in all these 19 and 20 steps synthesis, 13 steps could be conducted without column chromatography.^125^

### 5.9. Total syntheses of phleghenrines A and C

**Scheme 62:**
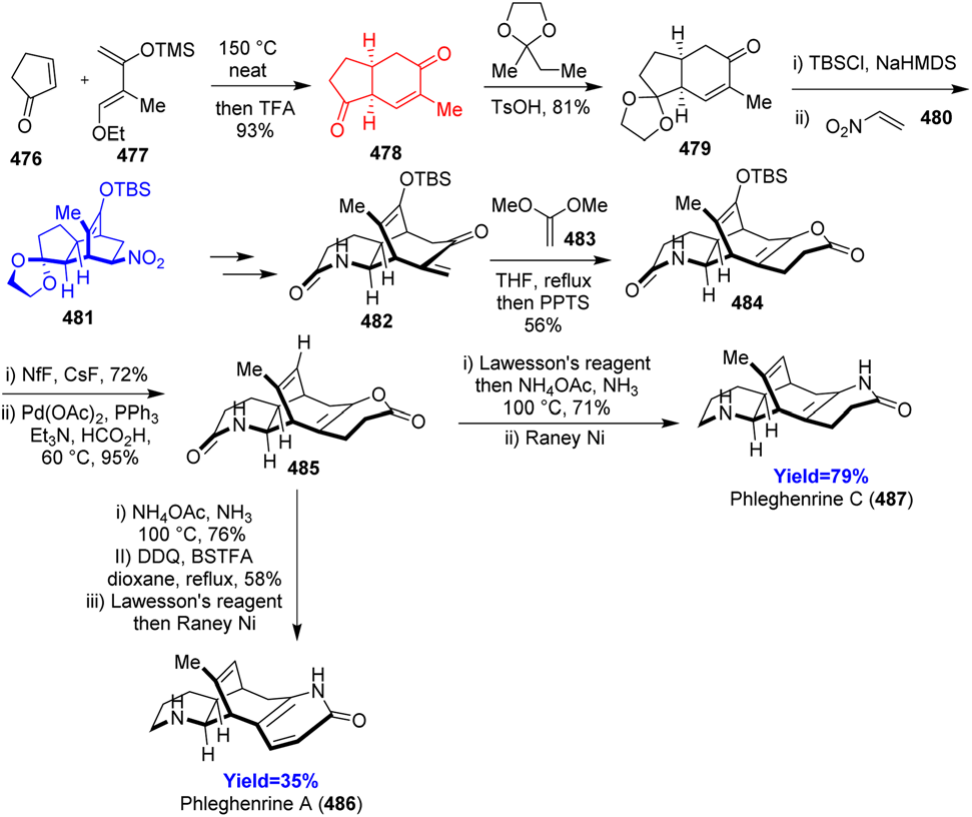
Total syntheses of phleghenrines A (486) and C (487).^126^

*Lycopodium* alkaloids phleghenrines A and C have same [6-6-7-6] tetracyclic scaffold where bicyclo[3.2.2]nonane being the central core and two aza heterocycles are fused in left and right side. Shi *et al.*^126^ utilized three Diels–Alder reactions in their 19 and 18 steps total synthesis of phleghenrines A and C respectively starting from cyclopentenone 476 and Danishefsky's diene 477 (Scheme 62). The Diels–Alder adduct 478 then chemoselectively protected and again underwent Diels–Alder reaction with dienophile nitro ethylene 480. The adduct 481 converted to last hetero-Diels–Alder precursor 482 and reacted with 1,1-dimethoxyethylene 483 which can further form the final phleghenrines 486, 487 in 3 to 4 steps.^126^

### 5.10. Synthesis of 6-azaindoles and total synthesis of marinoquinoline A

Marinoquinolines possess potent inhibitory effect on enzyme acetylcholine esterase and weak anti-bacterial, anti-fungal, anti-malarial activities and cytotoxic properties.^127^

**Scheme 63:**
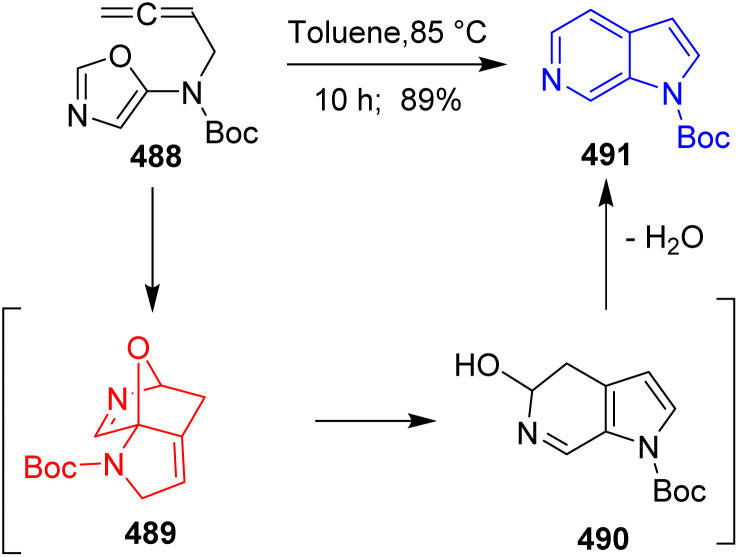
IMDAO reaction of allenyl oxazole (488) formed 6-azaindole (491).

Osano *et al.*^128^ developed intramolecular Diels–Alder oxazole (IMDAO) of allenyl oxazole 488 to form 6-azaindole 491 (Scheme 63), the core structure of marinoquinoline A.

If alkyne is present in place of allenes, the alkyne–allene isomerization take place to form the allene followed by IMDAO.

**Scheme 64:**
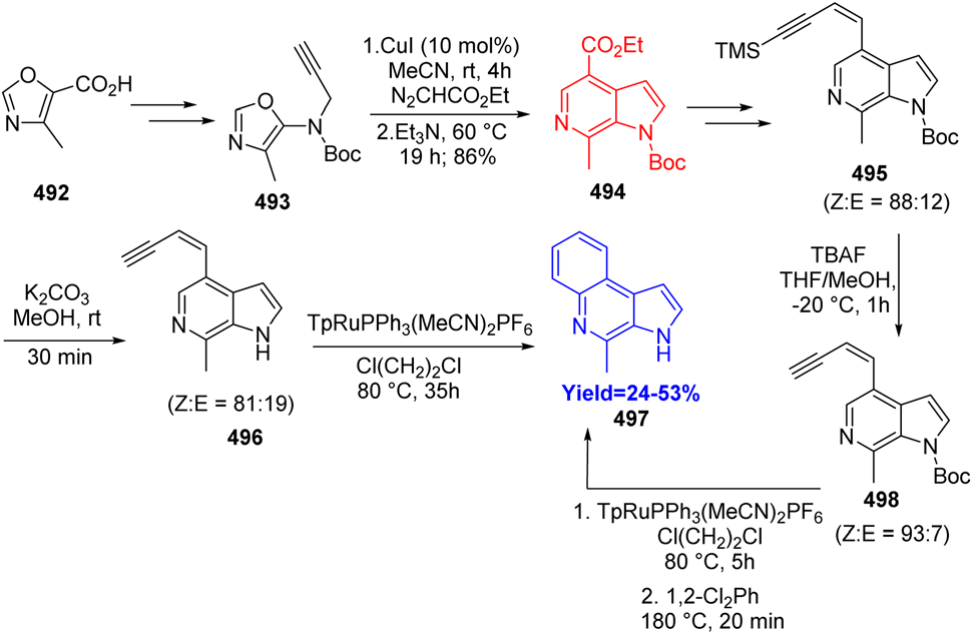
Total synthesis of marinoquinoline A (497).^128^

After forming the substituted aza-indole, marinoquinoline A 497 was synthesized by Osano *et al.* in the following pathway (Scheme 64). Starting from compound 492, intermediates 495, 496 were prepared by several transformations. Both from 495 and 496, pyrroloquinoline marinoquinoline A 497 was prepared however, the yields get doubled when the starting substrate was choosed 495.^128^

## 6. Photoenolization Diels–Alder (PEDA) reaction

### 6.1. Total synthesis of aglacins A, B, and E

**Scheme 65:**
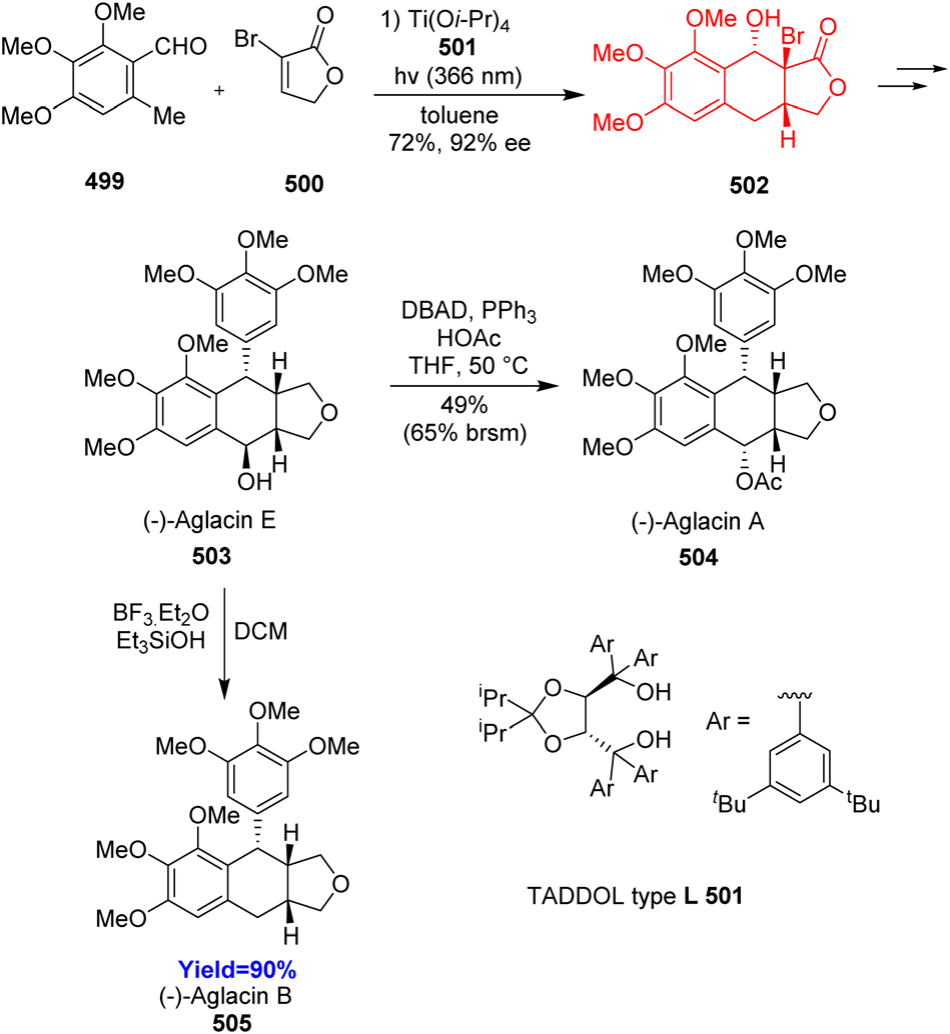
Total synthesis of aglacins A (504), B (505), and E (503).^132^

Podophyllotoxin exhibits different kinds of biological activities, such as anti-cancer, insecticidal, anti-fungal, anti-viral, anti-inflammatory, neurotoxic, immunosuppressive activities.^129–131^ Aglacin A, B, E are biologically related natural products to podophyllotoxin. These compounds belong to the aryltetralin cyclic ether lignans. They consist of tetracyclic fused ring containing aryltetralin ring (A–B), two oxygenated aromatic rings (A, D), γ-lactone ring (C), and consecutive four chiral centres in B ring. Xu *et al.*^132^ proposed two pathways to construct the core skeleton of lignans through PEDA reaction in the retrosynthetic analysis. When Xu and co-workers performed choosen two pathways, only the B pathway gave better results. Total synthesis commenced with asymmetric photoenolization Diels–Alder reaction between 499 and α-bromolactone 500. The cycloadduct 502 was converted to aglacin E 503 in 12 steps which then can be converted to aglacin A 504 by intermolecular Mitsunobu reaction and to aglacin B 505 through reductive dehydroxylation (Scheme 65).^132^

### 6.2. Synthesis of naphthol and naphthalene core

**Scheme 66:**
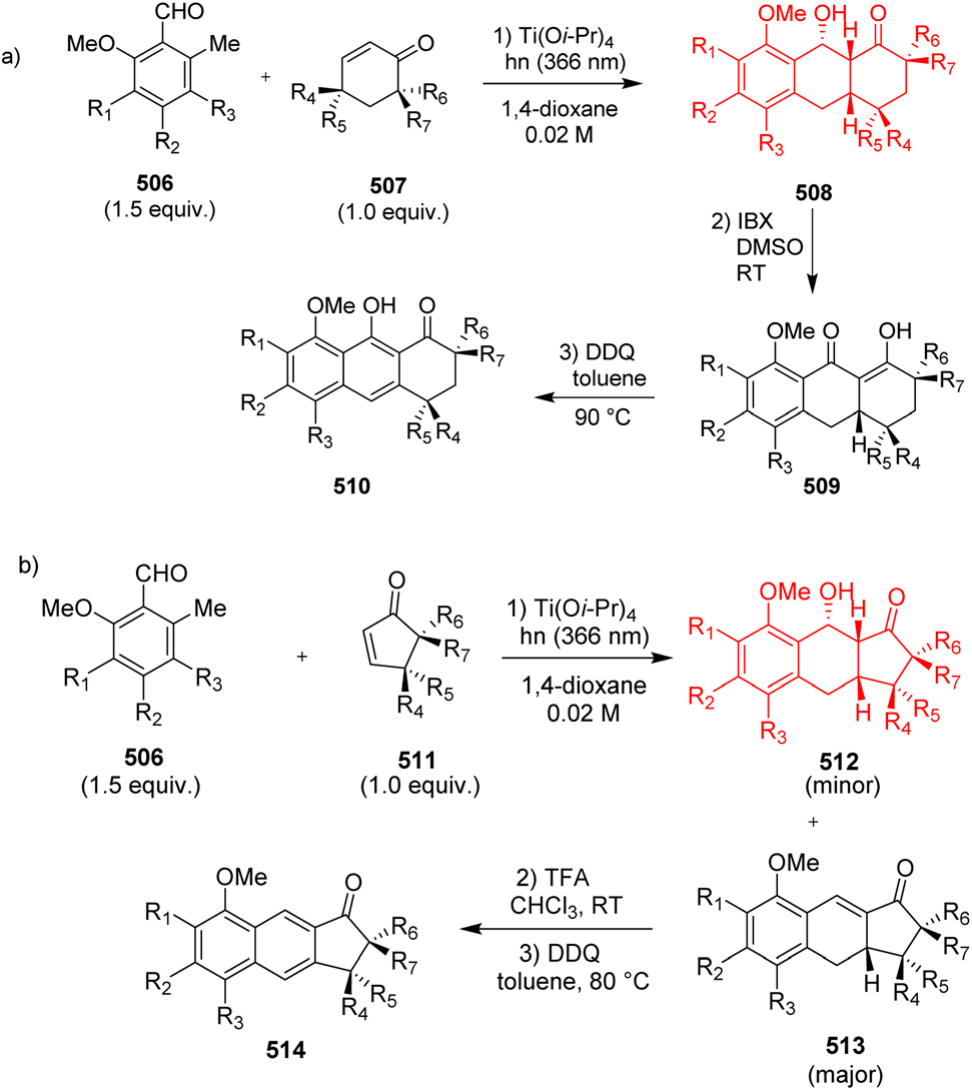
(a) PEDA reaction with cyclohexenone dienophile (507), (b) PEDA reaction with cyclopentenone dienophile (511).^133^

Many natural products such as garveatins A–D, exiguaquinol contain the naphthol and naphthalene core. These natural products show anti-microbial, anti-bacterial activities.^133^ Diels–Alder reactions or PEDA reactions play important role in constructing the naphthol and naphthalene scaffolds. Lu *et al.*^133^ have used sterically hindered dienophiles like α,α-disubstituted, γ,γ-disubstituted or α,α,γ,γ-tetrasubstituted cyclohexenones 507 and cyclopentenones 511 having a quaternary center adjacent to the enone. The product 510 synthesized from cyclohexenone dienophiles generated the core of garveatin A–D, whereas the product 514 synthesized from cyclopentenone dienophile formed the core of exiguaquinol (Scheme 66).^133^

### 6.3. Total synthesis of perovskones, hydrangenone, hydrangenone B

**Scheme 67:**
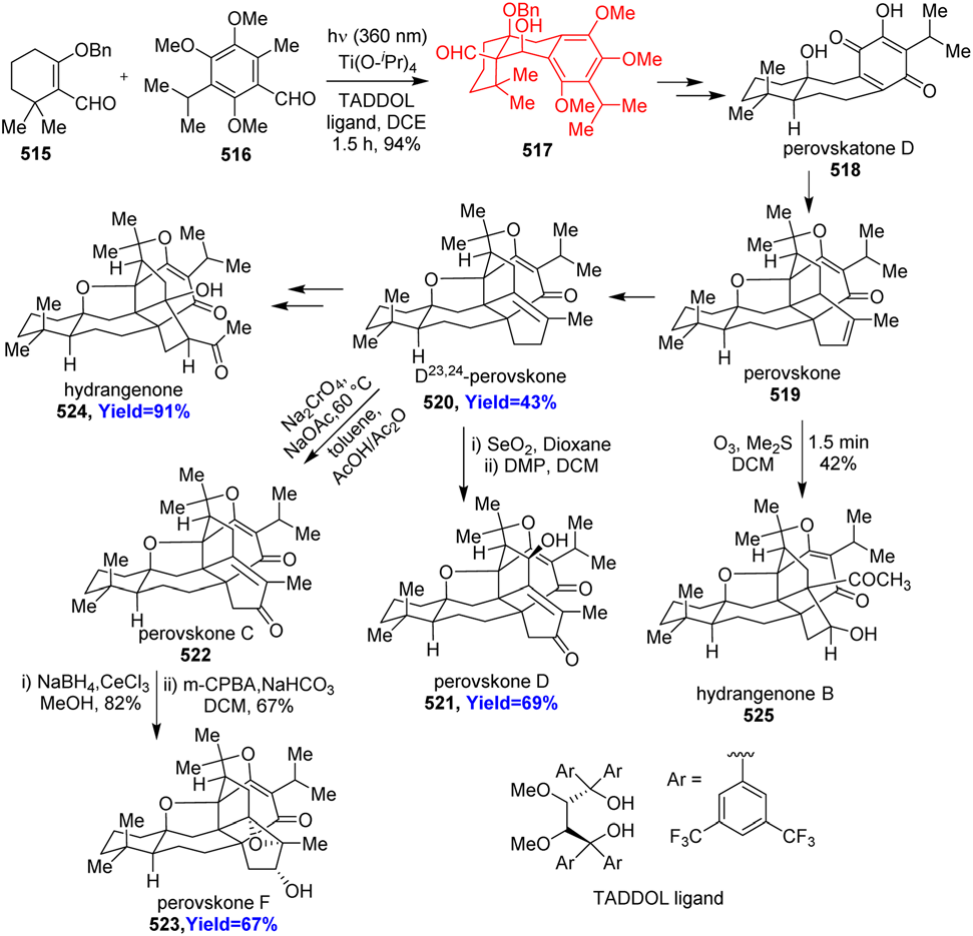
Total synthesis of perovskones (519–523), hydrangenone (524), hydrangenone B (525).^134^

Triterpenoids perovskones and hydrangenones were isolated from *Salvia* medicinal plants. Biological activities like anti-tumor, anti-plasmodial activities were shown by these complex natural products.^134^ Yang and co-workers^134^ commenced the total synthesis with asymmetric photoenolization/Diels–Alder reaction between 515 and 516 in the presence of a TADDOL type ligand and Ti(O-^i^Pr)_4_ (Scheme 67). The Diels–Alder adduct 517 was converted to perovskatone D (518), which is biosynthetic precursor of perovskones and hydrangenones. Perovskatone D 518 treated with *trans*-α-ocimene, Eu(fod)_3_ and the product underwent intramolecular Diels–Alder reaction. After some tandem process Δ^23,24^-perovskone 520 produced through perovskone 519. The latter led to the hydrangenone B 525 while the former led to other perovskones 521–523 and hydrangenone 524.^134^

## 7. Inverse electron demand Diels–Alder (IEDDA) reaction

### 7.1. Synthesis of chiral [2,3]-fused indolines

**Scheme 68:**
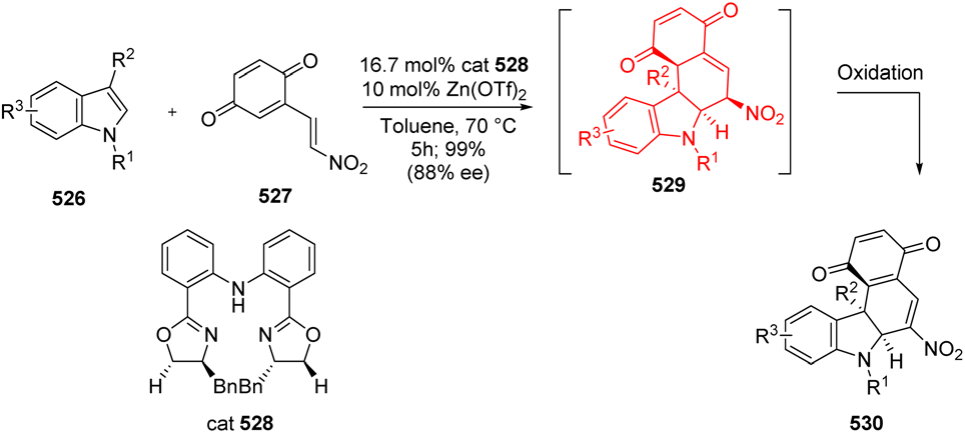
IEDDA reaction to form the [2,3]-fused indoline scaffold.^135^

[2,3]-Fused indolines are core structure of many naturally occurring alkaloids having medicinal properties. Zhao *et al.*^135^ developed chiral [2,3]-fused indoline derivatives 530 by an enantioselective dearomatization IEDDA reaction (Scheme 68).^135^ Zhao and co-workers^135^ started this chiral bisoxazoline/zinc-catalyst-complex mediated reaction with 2-(2-nitrovinyl)-1,4-benzoquinone 527 (diene) and a highly substituted indole 526.

### 7.2. Construction of *cis*-hydroindole scaffolds and total synthesis of minovincine

**Scheme 69:**
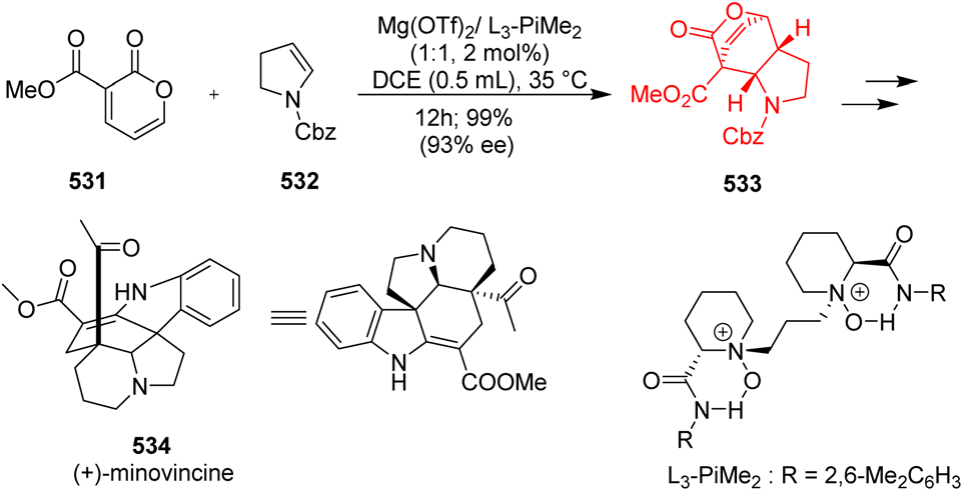
IEDDA reaction of 2-pyrones 531 with enamine 532 in total synthesis of (+)-minovincine (534).^138^

*cis*-Hydroindole scaffold is present in many natural products like minovincine, kopsinine, vindolinine, aeruginosin, pharmaceuticals like anti-hypertensive drug perindopril.^136,137^ Zhang *et al.*^138^ had started with 3-carbomethoxy-2-pyrone 531 and cyclic enamine 532 as substrates (Scheme 69). This reaction showed wide substrate scope, and also compatible with acyclic enamines. The chiral ligand coordinated with Mg(OTf)_2_ in a tetradentate manner to form a octahedral structure. 2-Pyrone coordinated with its two carbonyl groups of ester to the Lewis acid catalyst and lowered the LUMO level to accelerate the IEDDA reaction. The bulky amide group of the ligand causes steric hindrance, so cyclic enamine prefers to attack the Si-face of 2-pyrone to produce *endo*-adduct with excellent stereoselectivity. The DA adduct 533 was transformed to form the natural product (+)-minovincine 534.^138^

### 7.3. Total syntheses of cephanolides A and B

**Scheme 70:**
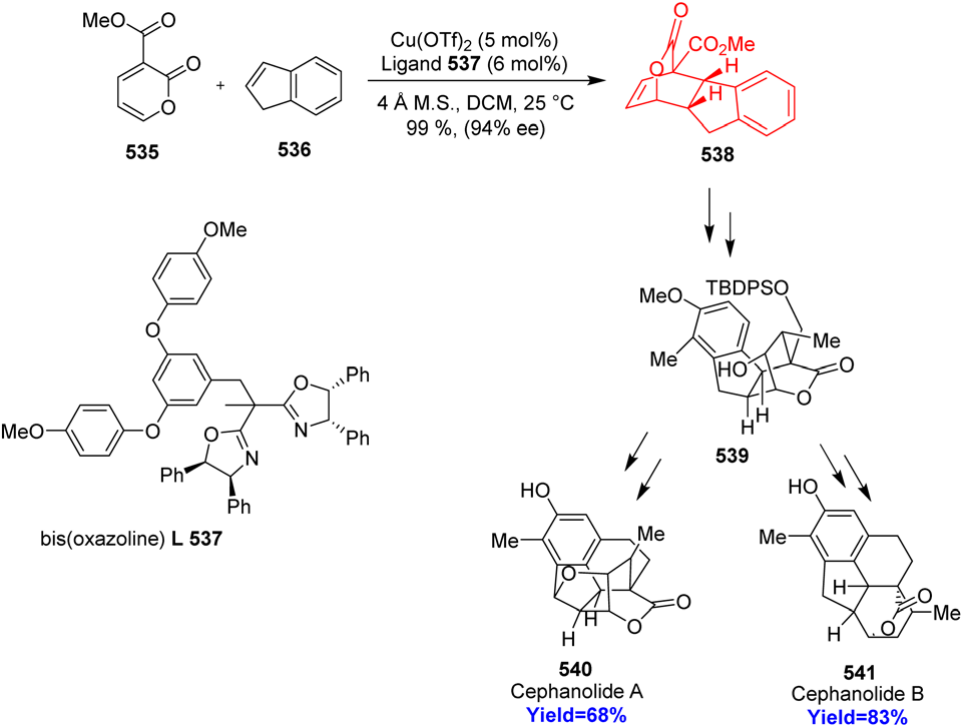
IEDDA reaction of 2-pyrones 535 with indane 536 in the total synthesis of cephanolide A (540), and cephanolide B (541).^140^

Cephanolides belong to the *Cephalotaxus* diterpenoids class of natural products, isolated from *Cephalotaxaceae* plants. These well-known natural products have important biological activities especially potent anti-tumor activity.^139^ Cephanolide B has same pentacyclic core like cephanolide A but there is an extra tetrahydrofuran ring in cephanolide A. Lu and co-workers^140^ disclosed a Cu catalyzed asymmetric IEDDA reaction with an electron deficient 2-pyrone 535 and indene 536 which produced hexahydrofluorenyl bridged-lactone scaffold 538 (Scheme 70). This scaffold 538 is common for the cephanolides A–D and could serve as an ideal precursor for natural product synthesis. 538 was converted to the common intermediate 539 and completed the total syntheses of cephanolides A 540 and B 541 in 13 and 14 steps more respectively.^140^

### 7.4. H-Bonding mediated intramolecular IEDHDA reaction to access tricyclic tetrahydropyran derivatives

**Scheme 71:**
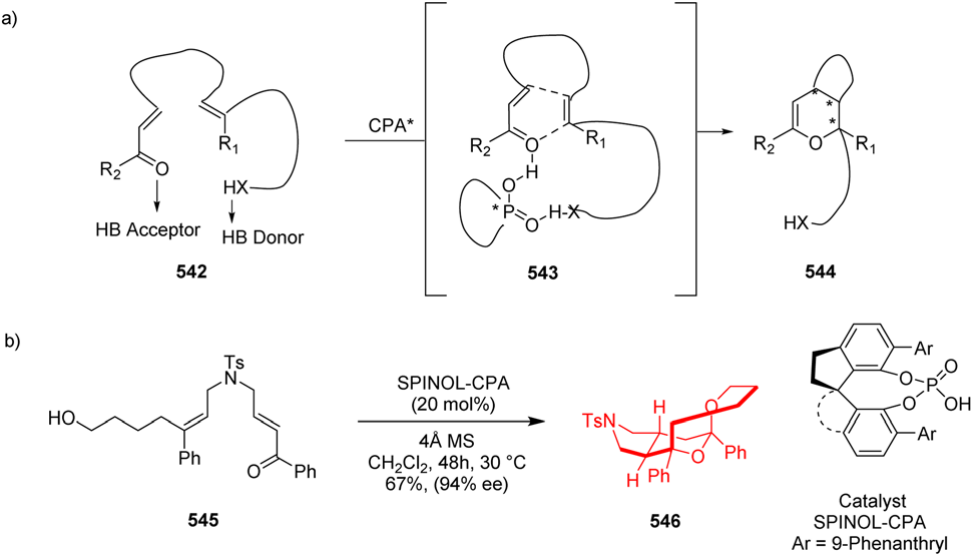
(a) IEDHDA reaction *via* dual hydrogen bonding interaction, (b) formation of tricyclic tetrahydropyran derivatives *via* intramolecular IEDHDA reaction of 545.^141^

Jin *et al.*^141^ had recently developed a method to produce tricyclic tetrahydropyran derivative by intramolecular IEDHDA reaction between neutral alkene and α,β-unsaturated ketones or aldehydes. It was the first example where a simple α,β-unsaturated ketone or aldehyde has been used with neutral alkene in intramolecular IEDHDA. The non-activated alkenes could be cyclized with α,β-unsaturated ketones or aldehydes with chiral phosphoric acid through double H-bonding (Scheme 71a). Substrate 545 had a hydroxyl hydrogen bonding donor group and an α,β-unsaturated arylketone as hydrogen bond acceptor. Catalyzed by SPINOL-CPA ligand, those two moieties in 545 came in close proximity and performed the cycloaddition reaction (Scheme 71b). *Z*-Configured substrate didn't perform in this reaction.^141^

### 7.5. Synthesis of *cis*-decalin derivatives and total synthesis of 4-aminophen-11-ol and *cis*-crotonin

**Scheme 72:**
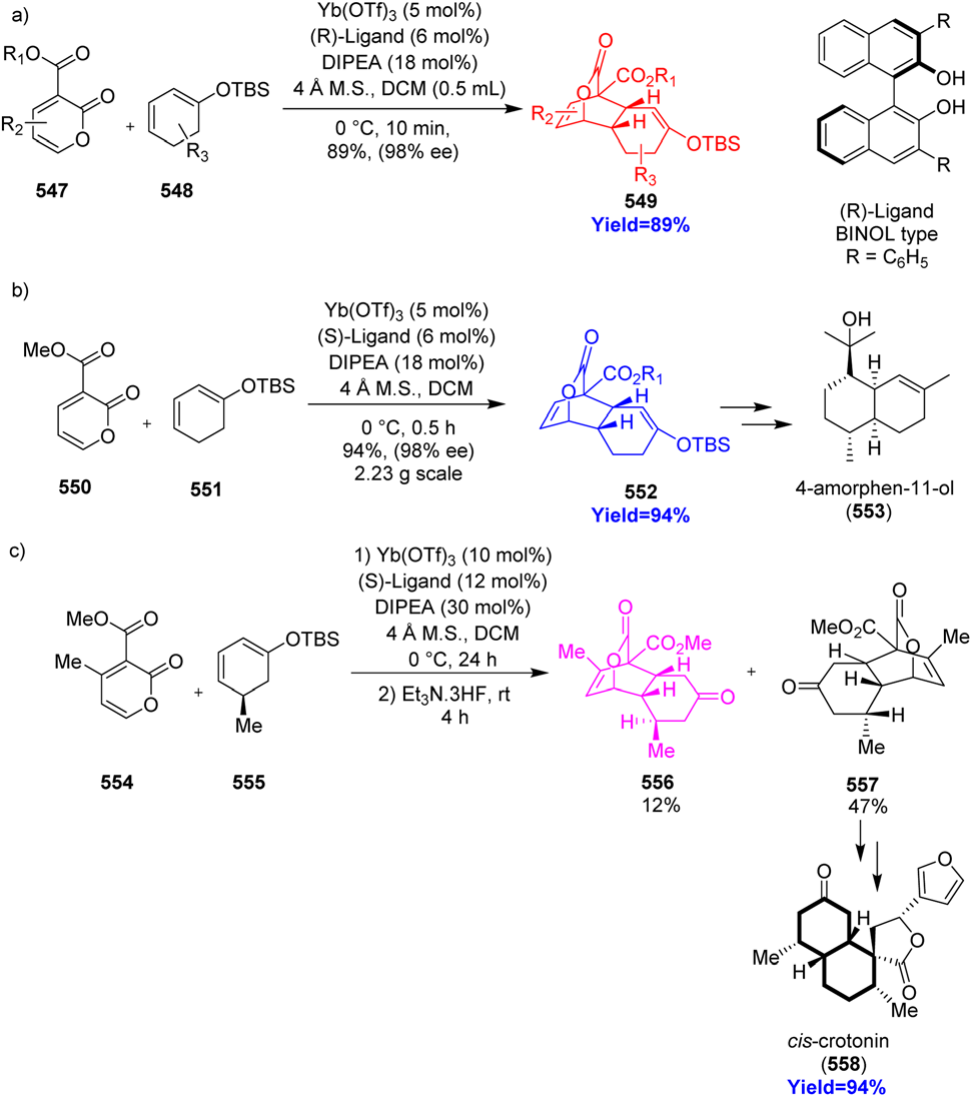
(a) IEDDA reaction to construct *cis*-decalin motif in 549, (b) total synthesis of 4-amorphen-11-ol (553), (c) total synthesis of *cis*-crotonin (558).^142^

*cis*-Decalin cores are present in various bioactive natural products and drugs. Si *et al.*^142^ have reported the formation of *cis*-decalin core by an ytterbium-catalyzed asymmetric IEDDA. They also showed the potential of their synthesis by providing enantioselective total synthesis of 4-amorphen-11-ol and *cis*-crotonin. 4-Amorphen-11-ol is one type of sesquiterpene natural products of the *Fabiana imbricate* medicinal plant and crotonins belong to the clerodane diterpenes. Si and co-workers performed inverse-electron-demand Diels–Alder reaction between 3-carboalkoxyl-2-pyrone 547 and silyl cyclohexadienol ether 548 in the presence of Yb(OTf)_3_/BINOL (Scheme 72a). Moreover, enantioselective total synthesis of 4-amorphen-11-ol 553 (Scheme 72b) and *cis*-crotonin 558 (Scheme 72c) was done *via* this reaction.^142^

### 7.6. Total synthesis of lissodendoric acid A

**Scheme 73:**
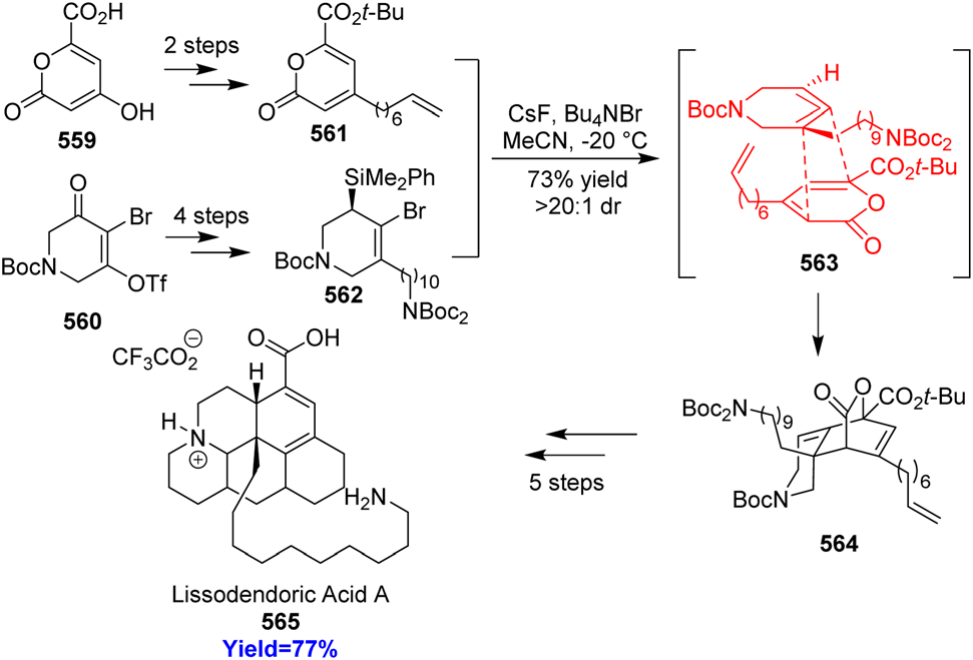
Total synthesis of lissodendoric acid A (565).^143^

Lissodendoric acid A, isolated from marine sponge *Lissodendoryx florida*, belongs to the manzamine family of alkaloids. The structural feature of lissodendoric acid A is it holds an azadecalin scaffold and 14-membered macrocycle. During the total synthesis, Ippoliti *et al.*^143^ had trapped a cyclic allene intermediate which is very rare also in a Diels–Alder reaction. The two Diels–Alder precursor pyrone 561 and silylbromide 562 (cyclic allene precursor) were synthesized from commercially available carboxylic acid 559 and bromotriflate 560 respectively (Scheme 73). The inverse electron demand Diels–Alder reaction was successfully done in the presence of CsF, Bu_4_NBr at −20 °C to construct the azadecalin core. The cycloadduct 564 completed the total synthesis of lissodendoric acid A 565 in 5 steps.^143^

### 7.7. Synthesis of *cis*-decalin motifs

**Scheme 74:**
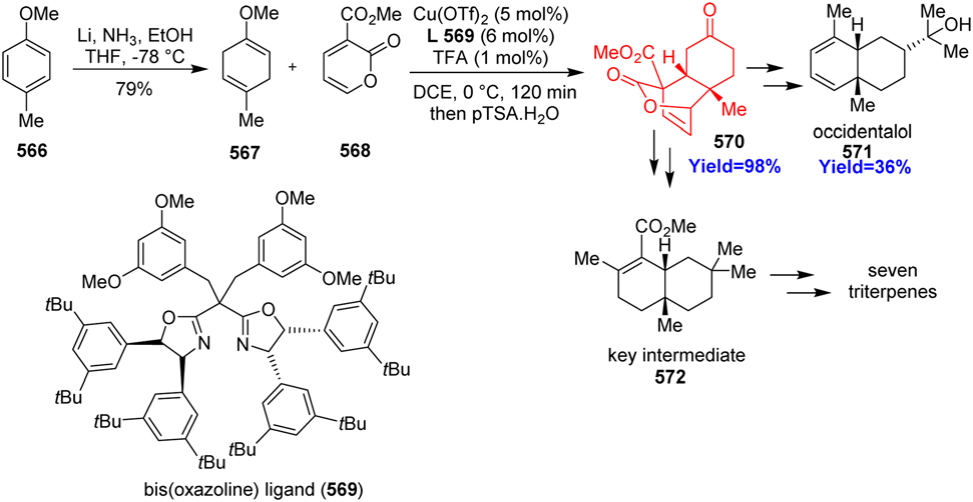
Synthesis of *cis*-decalin motifs.^144^

Since, *cis*-decalin motifs are hugely found in natural products and in bioactive compounds it gains much attention from chemists. Si and co-workers^144^ had developed a unique way to construct the *cis*-decalins through multi-step synthesis. Birch reduction of 566 to 567 merged with inverse-electron-demand Diels–Alder reaction between 567 and 568 in presence of Cu(OTf)_2_ catalyst, a bis(oxazoline) ligand provided the *cis*-decalins 570 (Scheme 74). This reaction produced a wide range of polysubstitued *cis*-decalins, key intermediate 572 to several triterpene and had led to the concise total synthesis of occidentalol (571).^144^

### 7.8. Concise total synthesis of lucidumone

**Scheme 75:**
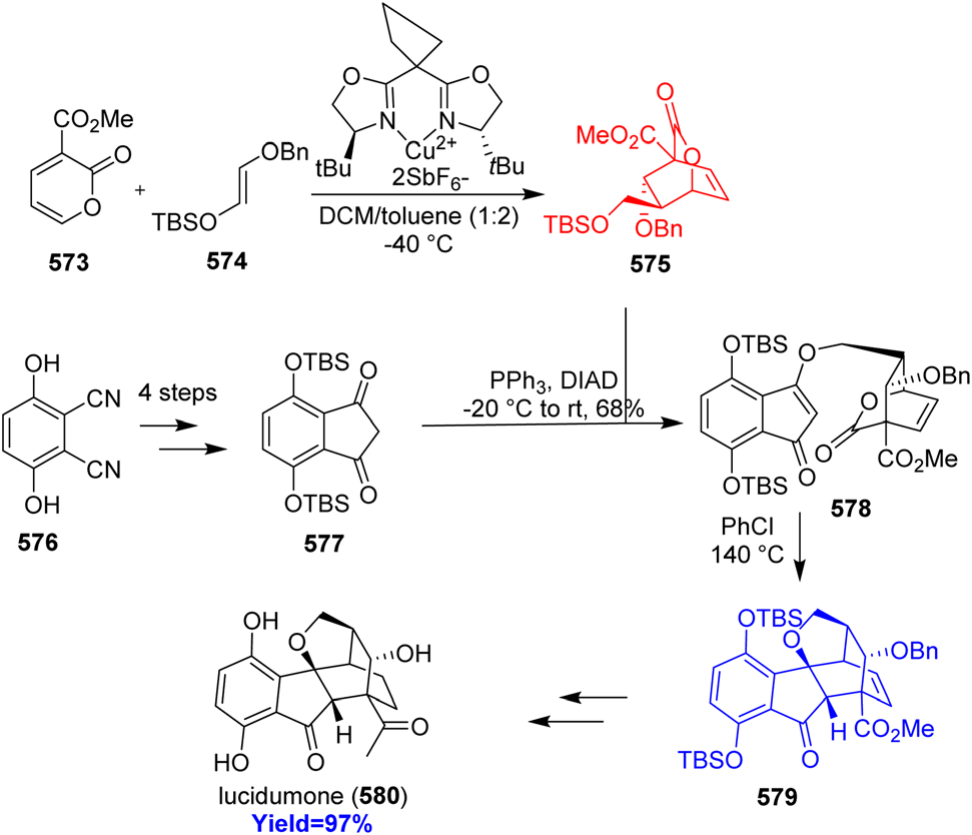
Concise total synthesis of lucidumone (580).^145^

Lucidumone, extracted from *Ganoderma lucidum*, inhibited COX-2, showed anti-inflammatory activity and has a very complex structure with bicyclo[2.2.2]scaffold. Huang and co-workers^145^ synthesized this complex lucidumone natural product through a retro-[4+2]/intramolecular Diels–Alder cascade. Inverse-electron-demand Diels–Alder reaction between 2-pyrone 573 and acyclic enol ether 574 formed a lactone 575 which then combined with indanedione 577 prepared from readily available dicyanodihydroquinone 576 (Scheme 75). The product 578 then underwent retro-[4+2]/IMDA cascade to form 579 and finally converted to lucidumone 580 in few steps.^145^

### 7.9. Total synthesis of alstonlarsine A

**Scheme 76:**
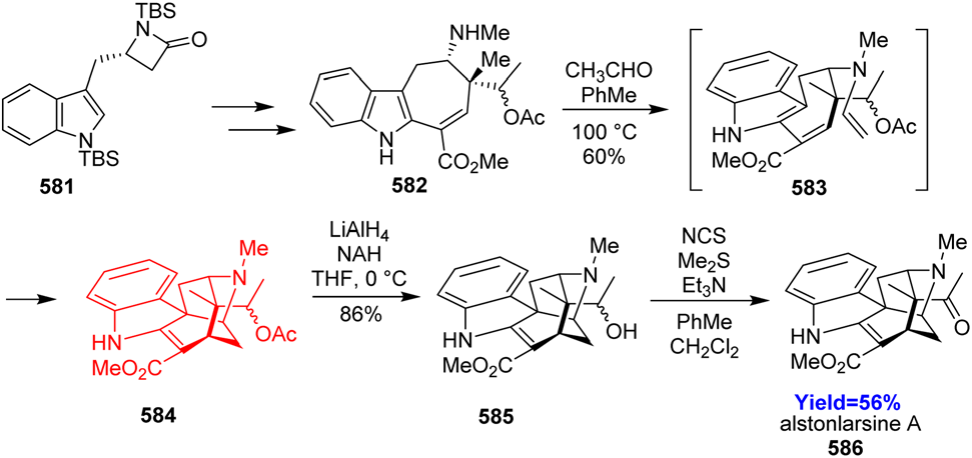
Total synthesis of alstonlarsine A (586).^147^

9-Azatricyclo[4.3.1.0^3,8^] decane motif fused with indoline ring is a unique feature of alstonlarsine A which was isolated from *Alstonia scholaris*. Alstonlarsine A is known for its Drak2 inhibitory activity which in turn a promising drug target for autoimmune diseases.^146^ Based on their retrosynthetic analysis, Ferjancic *et al.*^147^ commenced with homotryptophan β-lactam 581 (Scheme 76). The β-lactam 581 was converted to the key intermediate 582 through intramolecular Horner–Wadsworth–Emmons reaction to form the cycloheptane ring. Amine 582 formed enamine 583 when heated with acetaldehyde and the enamine 583 underwent inverse-electron-demand dearomative Diels–Alder reaction to form 584. This domino sequence followed by selective deprotection of acetate group and Corey–Kim oxidation completed the total synthesis of alstonlarsine A 586.^147^

### 7.10. Collective syntheses of illudalane sesquiterpenoids

**Scheme 77:**
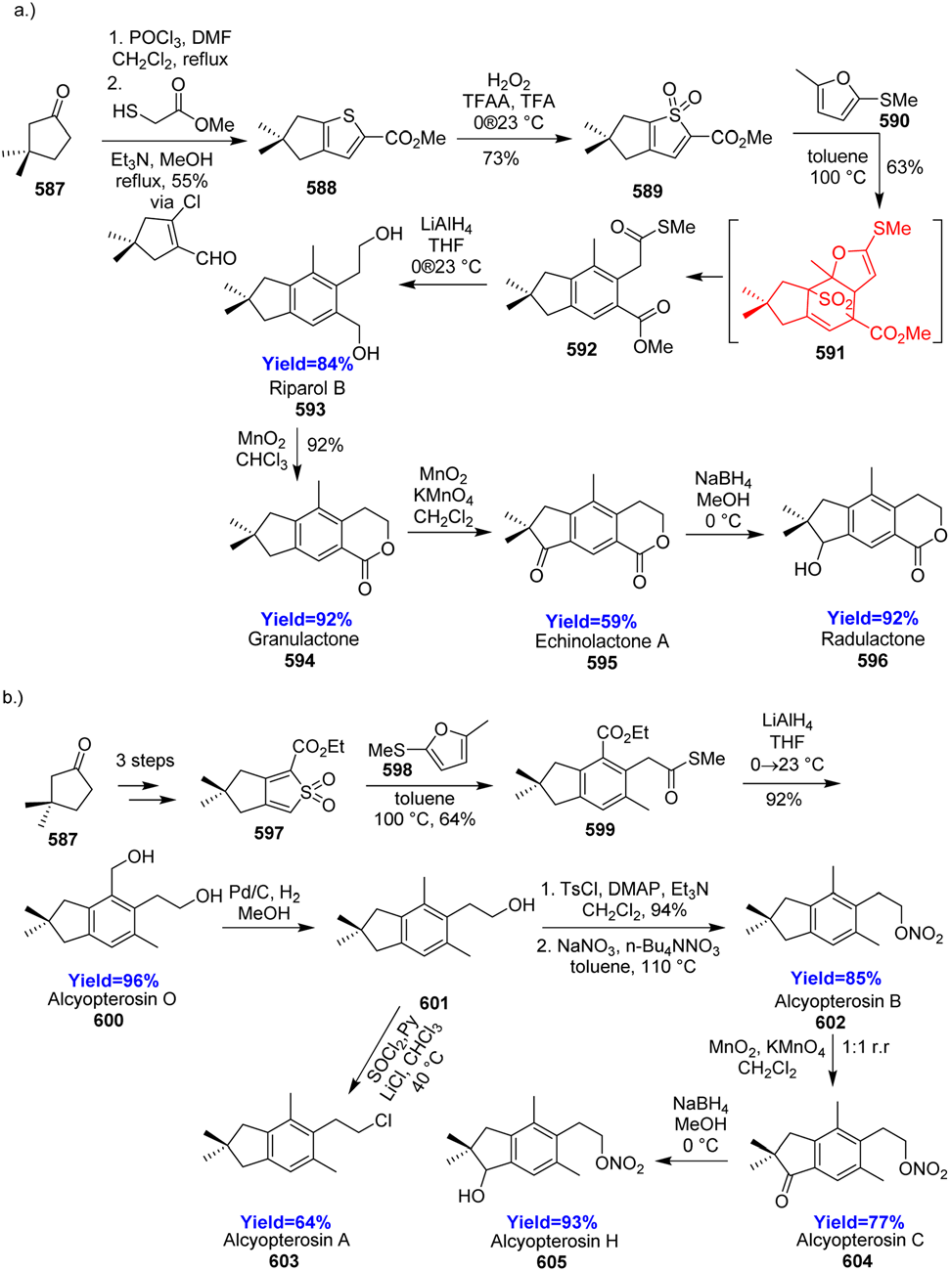
Collective syntheses of illudalane sesquiterpenoids (a) synthesis of riparol B, granulolactone, echinolactone A, radulactone (b) synthesis of alcyopterosins.^148^

Polysubstituted benzene rings are commonly found in illudalane sesquiterpenoids. Though there are several strategies to form benzene rings, Park and co-workers^148^ took a different approach through inverse-electron demand Diels–Alder cycloaddition/retro-cycloaddition with SO_2_ extrusion. The diene 2,3-fused bicyclic thiophene *S*,*S*-dioxide 589 was synthesized from 3,3-dimethylcyclopentanone 587 and underwent Diels–Alder reaction with dienophile furan 590 to construct the core of illudalane benzenoid 592 (Scheme 77a). This cycloadduct 592 then could be converted to few illudalane sesquiterpenoids 593–596 consecutively.^148^ Alcyopterosins O, A, B, C, H were also synthesized through similar kind of reactions only by changing the reaction components (Scheme 77b).^148^

### 7.11. Total syntheses of cephanolides and ceforalides

**Scheme 78:**
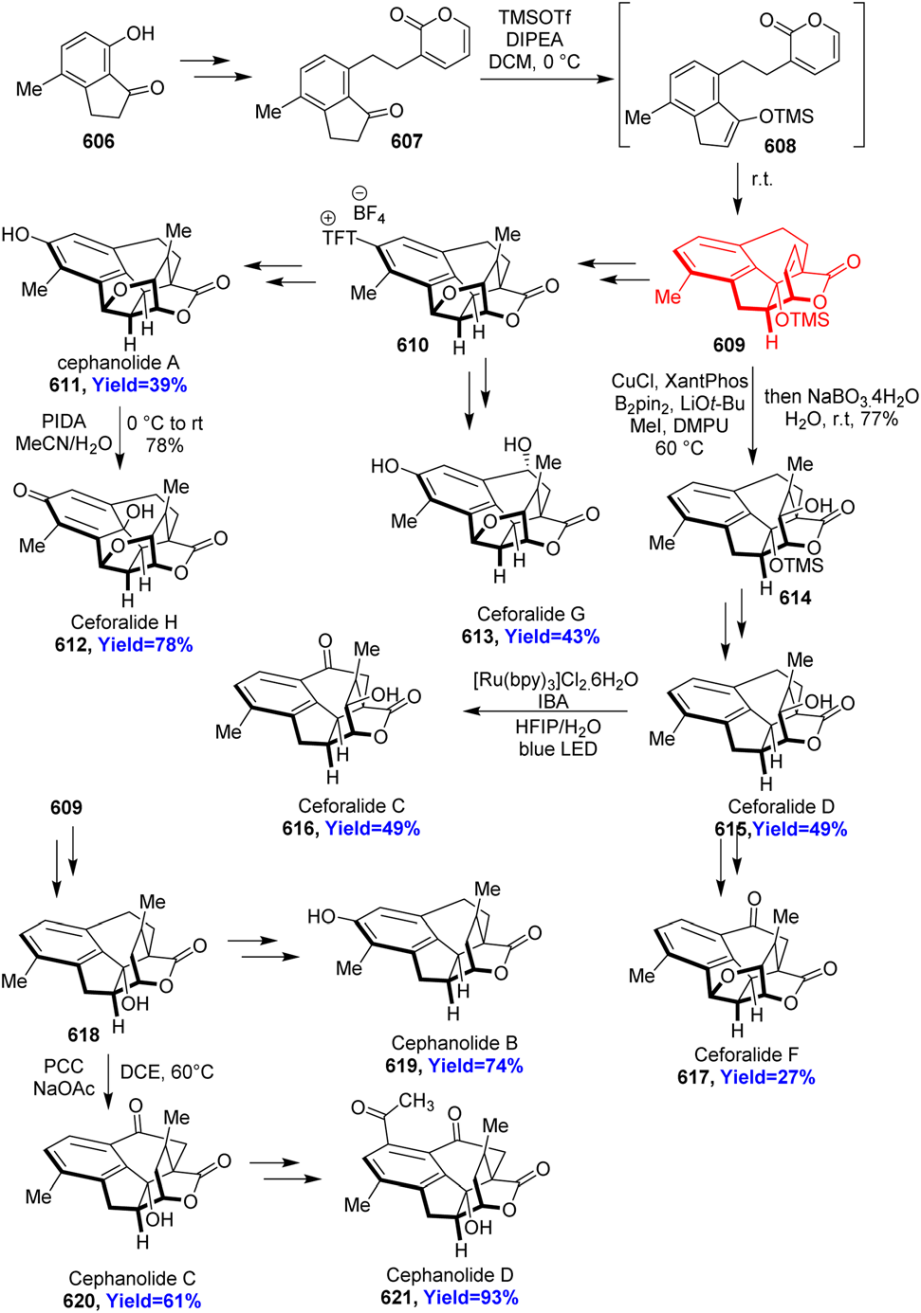
Total syntheses of cephanolides and ceforalides.^149^

Cephanolides and ceforalides, both belong to the *Cephalotaxus* diterpenoids, were isolated from *Cephalotaxus sinensis* and seeds of *Cephalotaxus fortune* respectively. Sennari and co-workers^149^ commenced the total synthesis with hydroxyindanone 606 and converted it to the Diels–Alder precursor 607 through repetitive cross-coupling (Scheme 78). The intramolecular inverse-electron demand Diels–Alder reaction produced the desired product 609 successfully with high yield and finally synthesized cephanolide A 611*via*610. 610 synthesized ceforalide G 613 while cephanolide A 611 formed ceforalide H 612. Moreover 609 was converted to 618 which formed cephanolides B–D 619–621 in few steps and to 614 which formed ceforalide D 615, C 616 and F 617.^149^

### 7.12. Total synthesis of beraprost

**Scheme 79:**
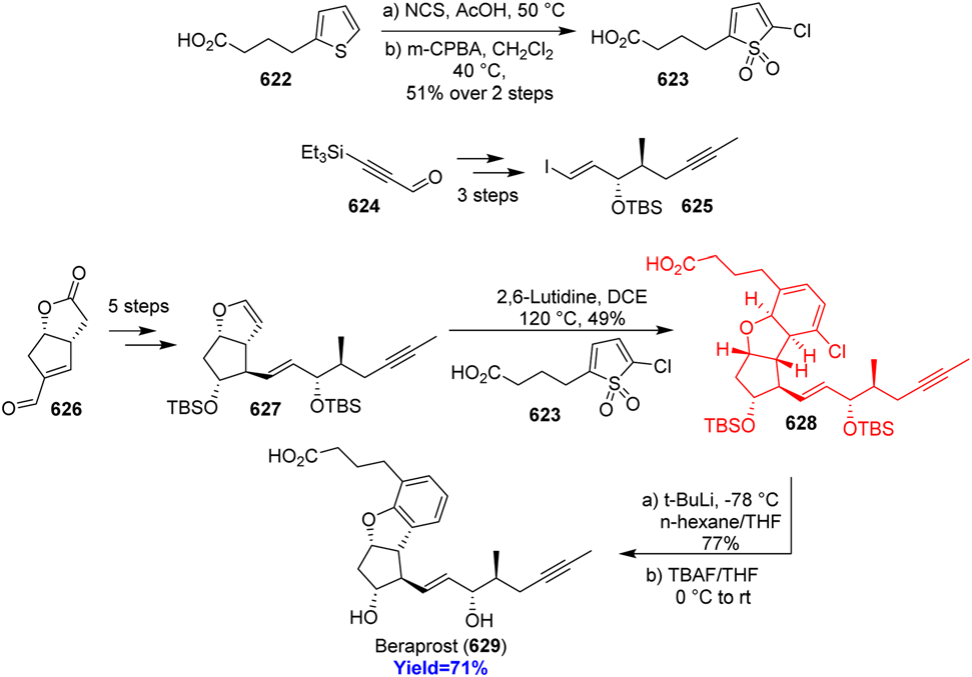
Total synthesis of beraprost (629).^150^

Antiplatelet drug beraprost, a less cytotoxic prostacyclin analogue, used for the treatment of pulmonary arterial hypertension (PAH) and can be taken orally. Wang *et al.*^150^ based on their retrosynthesis commenced the synthesis from commercially available thiophene 622 and ynal 624 to form the upper sidechain 623 and lower sidechain 625 of beraprost respectively (Scheme 79). After forming the sidechains, enal-lactone 626 was converted to Diels–Alder precursor 627 in 5 steps. The precursor 627 and the upper sidechain containing thiophene 1,1-dioxide 623 on inverse-electron demand Diels–Alder reaction produced chlorocyclohexadiene 628 which then aromatized with *t*-BuLi and converted to beraprost 629 on deprotection with TBAF.^150^

### 7.13. Total synthesis of pedrolide

**Scheme 80:**
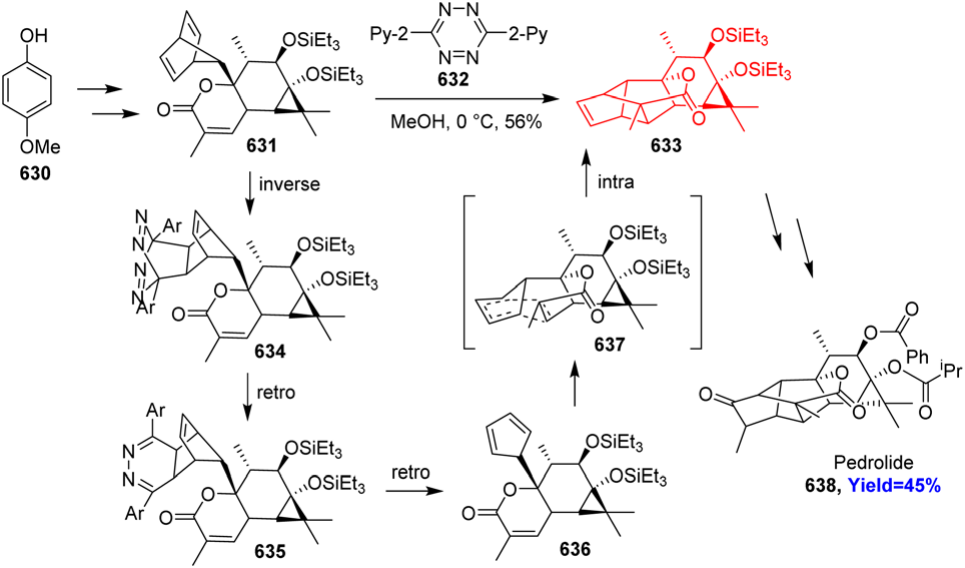
Total synthesis of pedrolide (638) through key Diels–Alder cascade.^151^

Pedrolide, a tigliane derived diterpenoid, isolated from *Euphorbia pedroi*. Highly oxidized carane fragment and a bicyclo[2.2.1]heptane (pedrolane scaffold) made it highly congested. Fadel and co-workers^151^ developed the first ever total synthesis of pedrolide with 4-methoxyphenol 630 as substrate (Scheme 80). A key Diels–Alder cascade was observed in constructing the bicycloheptane motif from 631 to 633. The cascade involves inverse-electron-demand Diels–Alder → retro-Diels–Alder → retro-Diels–Alder → intramolecular Diels–Alder reaction of 631 led to the bicycloheptane 633 successfully. Finally, the total synthesis of pedrolide 638 was completed in 20 steps with 45% yield.^151^

## 8. Inverse electron demand hetero-Diels–Alder reaction

### 8.1. Total synthesis of (−)-strictosidine

**Scheme 81:**
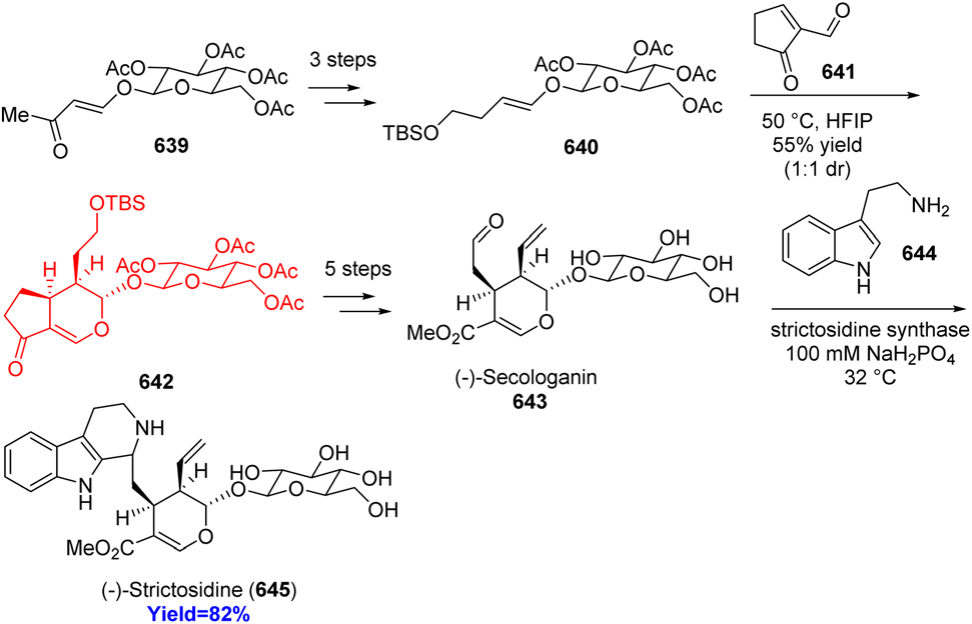
Total synthesis of (−)-strictosidine (645).^155^

MIAs (monoterpene indole alkaloids), are a large class of natural products possessing valuable pharmacological activities, derived from a single biosynthetic precursor strictosidine. Quinine, an anti-malarial drug; strychnine, a potent toxin; and vinblastine, a frontline anti-cancer drug are three most well-known members of MIA family.^152–154^ Anthony and co-workers^155^ synthesised strictosidine to access MIAs and their derivatives. They have taken a vinylogous ester 639 to produce enol ether 640 (Scheme 81). This enol ether 640 acted as a dienophile when reacted with a cyclopentenone (diene) 641 in hexafluoroisopropanol at 50 °C for 16 h. Among the four stereoisomeric transition states during this IEHDAR, the major product 642 was dictated by the orientation of the adjacent reactive olefin. The Diels–Alder adduct 642 was converted to secologanin 643 which produced strictosidine 645 using enzyme strictosidine synthase.^155^

### 8.2. Synthesis of tetrahydrochromeno[4,3-*b*]quinolines using Povarov reaction

**Scheme 82:**
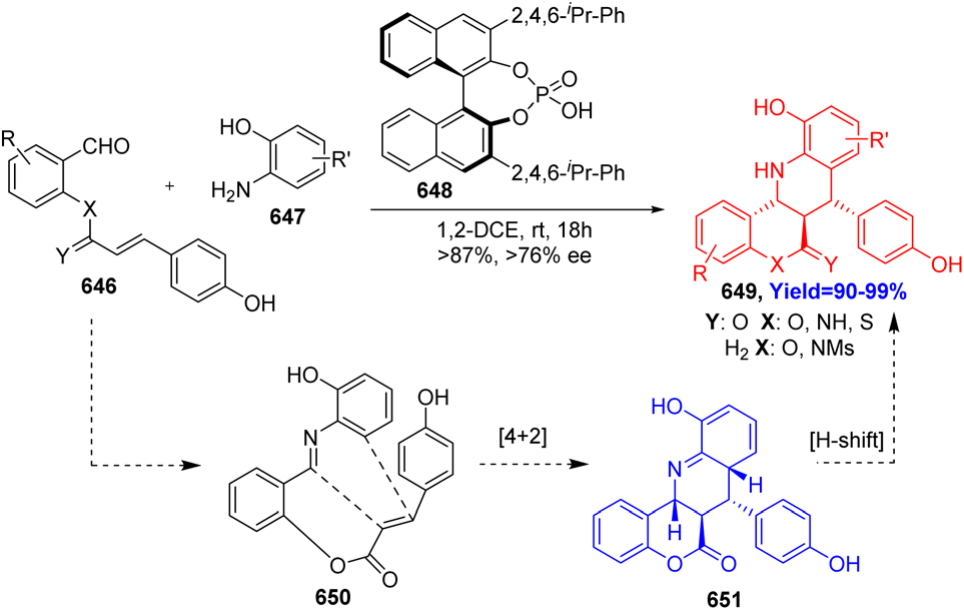
Synthesis of tetrahydrochromeno[4,3-*b*]quinolines using Povarov reaction.^159^

Tetrahydrochromeno[4,3-*b*]quinolines (THCQs) having tetrahydroquinoline scaffolds exhibit anti-bacterial, anti-cancer, anti-inflammatory properties including progesterone receptor modulation.^156–158^ Géraldine Masson^159^ and her colleagues reported that the Povarov reaction, specifically the intramolecular inverse-electron-demand aza-Diels–Alder (IEDADA) reaction, is one of the most straightforward methods to construct two fused rings in a single step. They have approached through 3-(4-hydroxyphenyl)acrylate 646 and primary anilines 647 formed the azadiene precursor 650. This precursor then underwent the Povarov reaction in the presence of a bulky chiral phosphoric acid 648, as illustrated in Scheme 82. Without any purification step diverse type of complex tetracyclic heterocycles 649 were produced with high yields and excellent diastereo, enantioelectivity. The two OH groups in the two substrates are crucial for the stereogenic outcome of the reaction.^159^

## 9. Conclusions

The review highlights and points out the key steps of the total synthesis involving DARs. The applications of these DARs lead to the formation of many natural product core skeleton and other motifs of bioactive compounds. Though Diels–Alder reaction was discovered in 1928, its developments and applications are still in the focus area of researches ongoing all over the world. Over the past few decades, the total synthesis of natural products has grown enormously and the role of Diels–Alder reactions in the total synthesis is unprecedented. The newer methodologies have demonstrated their high synthetic value by utilising many adaptations and variants as was highlighted by the numerous instances provided in this review.

Due to the effectiveness, adaptability, and scope of DAR, its use in the assembly of complex molecules is likely to be secured for a very long time. There are so many natural products which are undiscovered till now but will discover in the recent times. Those syntheses will probably require various types of DAR as a key step. So, the synthetic chemists must require this reaction in future as a tool for the total syntheses and will be keen to contribute to the longevity of DAR.

## Data availability

No data was used for the research described in the article.

## Author contributions

Anitesh Rana and Anupam Mishra contributed equally in this review article; Satish K. Awasthi: supervision editing and reviewing.

## Conflicts of interest

There are no conflicts to declare.
